# A Remote Secondary Binding Pocket Promotes Heteromultivalent Targeting of DC-SIGN

**DOI:** 10.1021/jacs.1c07235

**Published:** 2021-11-08

**Authors:** Robert Wawrzinek, Eike-Christian Wamhoff, Jonathan Lefebre, Mareike Rentzsch, Gunnar Bachem, Gary Domeniconi, Jessica Schulze, Felix F. Fuchsberger, Hengxi Zhang, Carlos Modenutti, Lennart Schnirch, Marcelo A. Marti, Oliver Schwardt, Maria Bräutigam, Mónica Guberman, Dirk Hauck, Peter H. Seeberger, Oliver Seitz, Alexander Titz, Beat Ernst, Christoph Rademacher

**Affiliations:** † Department of Biomolecular Systems, Max Planck Institute of Colloids and Interfaces, 14424 Potsdam, Germany; ‡ Department of Chemistry and Biochemistry, Freie University of Berlin, 14195 Berlin, Germany; § Department of Chemistry, Humboldt University of Berlin, 12489 Berlin, Germany; ∥ Departamento de Química Biológica e IQUIBICEN-CONICET, Universidad de Buenos Aires, C1428EHA Ciudad de Buenos Aires, Argentina; ⊥ Department of Pharmaceutical Sciences, University of Basel, 4056 Basel, Switzerland; # Chemical Biology of Carbohydrates, Helmholtz Institute for Pharmaceutical Research Saarland, Helmholtz Centre for Infection Research, 66123 Saarbrücken, Germany; ¶ German Centre for Infection Research, Campus Hannover-Braunschweig, 38124 Braunschweig, Germany; □ Department of Chemistry, Saarland University, 66123 Saarbrücken, Germany; ○ University of Vienna, Department of Pharmaceutical Sciences, Althanstrasse 14, 1090 Vienna, Austria; △ University of Vienna, Department of Microbiology, Immunology and Genetics, Max F. Perutz Laboratories, Biocenter 5, 1030 Vienna, Austria

## Abstract

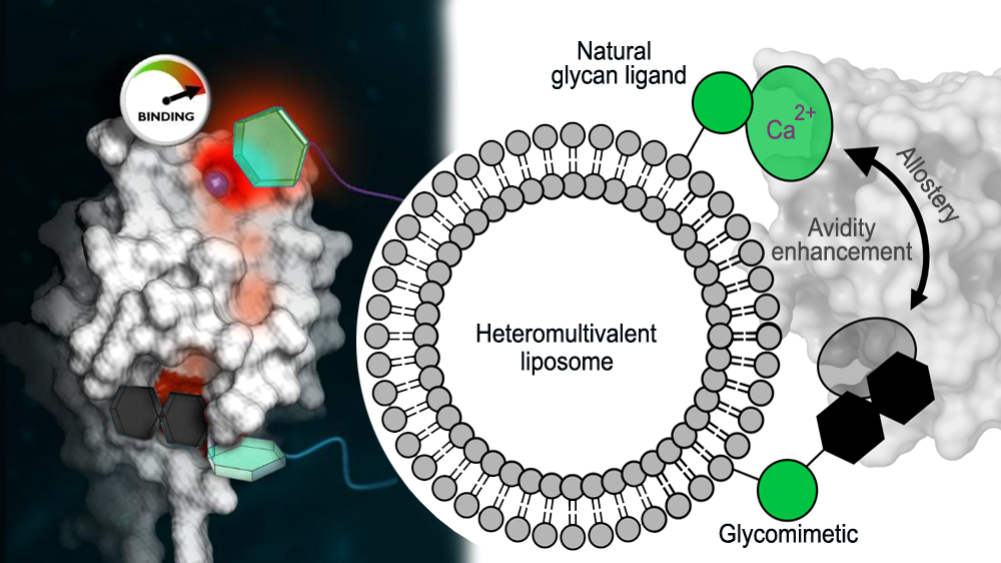

Dendritic cells (DC) are antigen-presenting cells coordinating the interplay of the innate and the adaptive immune response. The endocytic C-type lectin receptors DC-SIGN and Langerin display expression profiles restricted to distinct DC subtypes and have emerged as prime targets for next-generation immunotherapies and anti-infectives. Using heteromultivalent liposomes copresenting mannosides bearing aromatic aglycones with natural glycan ligands, we serendipitously discovered striking cooperativity effects for DC-SIGN^+^ but not for Langerin^+^ cell lines. Mechanistic investigations combining NMR spectroscopy with molecular docking and molecular dynamics simulations led to the identification of a secondary binding pocket for the glycomimetics. This pocket, located remotely of DC-SIGN’s carbohydrate bindings site, can be leveraged by heteromultivalent avidity enhancement. We further present preliminary evidence that the aglycone allosterically activates glycan recognition and thereby contributes to DC-SIGN-specific cell targeting. Our findings have important implications for both translational and basic glycoscience, showcasing heteromultivalent targeting of DCs to improve specificity and supporting potential allosteric regulation of DC-SIGN and CLRs in general.

## Introduction

Dendritic cells (DCs) constitute an integral part of the immune system, both in self- and in pathogen recognition. Working at the interface of innate and adaptive immunity, they can internalize viruses or bacteria and process exogenous antigens which they eventually present to CD4^+^ and CD8^+^ T cells. These characteristics render DCs attractive targets for antigen-specific immunotherapies, either to combat cancer or to develop prophylactic vaccines against viral and bacterial infections.^1−6^ A hallmark of DCs is the expression of several classes of endocytic receptors, including scavenger and chemokine receptors as well as C-type lectin receptors (CLRs).^7−9^ The latter are a class of pattern recognition receptors and of particular biomedical interest, as they display highly restricted expression profiles and also promote antigen cross-presentation.

Langerin (CD207) and DC-SIGN (CD209) represent two well-studied CLRs. The former is predominantly found on Langerhans cells, the major DC subset residing in the epidermis.^10−12^ As one of the first immune receptors to encounter pathogens entering the body via the skin, Langerin has been subject to targeting attempts employing either antibodies or glycomimetics.^4,13−17^ Found on both dermal DCs and macrophages, DC-SIGN shows a much broader expression profile.^18,19^ It also has been targeted, for instance, to develop cancer vaccines and antivirals, most prominently against HIV, which is known to hijack DC-SIGN to subsequently infect T cells.^18,20−22^

The challenging quest for glycomimetic targeting ligands has impeded clinical translation of DC-based immunotherapies: the shallow and highly solvent exposed carbohydrate binding site (CBS) of CLRs evolved to recognize hydrophilic glycans, found for example in the glycocalyx of pathogens. These features result in promiscuity and low intrinsic affinities, thereby complicating the identification of lead compounds in traditional drug discovery approaches.^23^ Nevertheless, several successful examples of glycomimetic ligand designs have been reported, many of which leverage the concept of fragment-based drug design with approaches reminiscent of fragment growing and linking.^13,24−26^

Under physiological conditions, glycan-receptor interactions take place in multivalent fashion, conveying avidity and selectivity to the otherwise transient and promiscuous nature of monovalent glycan recognition.^27,28^ Analogously, multivalency has been exploited to target CLRs or used in various assay systems to mimic and study these complex interactions.^29−33^ In addition to common homomultivalent targeting approaches, typically directed toward the primary CBS, simultaneously engaging secondary binding pockets represents a promising concept. The incentive here is to leverage avidity effects and to achieve higher receptor specificity, bypassing the overlapping glycan recognition profiles of lectins. Such approaches have been successfully implemented for other target classes but not for CLRs.^34,35^

For both Langerin and DC-SIGN, previous studies have revealed allosteric networks and potential secondary binding pockets.^13,17,36,37^ Moreover, allosteric inhibition was directly demonstrated for fragments binding to the murine ortholog of Langerin.^38^ For DC-SIGN, experimental evidence remains less conclusive with examples such as the identification of secondary binding pockets and proposed allosteric mechanisms for drug-like inhibitors discovered by Kiessling et al.^17,23,39^ Generally, the existence of such secondary binding pockets in CLRs not only warrants the reassessment of their druggability but could also lead to the design of innovative glycomimetic ligands. Notably, allosteric activation of CLRs, and lectins in general, has not been demonstrated to date.

In this study, we initially set out to discover glycomimetic ligands for Langerin. Here, we identified mannosides bearing an aromatic aglycone with micromolar affinity. However, when displayed multivalently on liposomes, we observed preferential binding to DC-SIGN^+^ cells. We further found striking cooperative effects for DC-SIGN when copresenting the glycomimetics with natural glycan ligands (i.e., mannose (**Man**), fucose (**Fuc**), and Lewis X (**LeX**)). Prompted by these findings, we investigated the underlying mechanisms by employing a combination of NMR spectroscopy, molecular docking, and molecular dynamics (MD) simulations. Two binding modes for the developed glycomimetics were discovered: a Ca^2+^-dependent interaction with DC-SIGN’s CBS as well as binding to a remote, secondary pocket. This leads to enhanced avidity for heteromultivalent liposomes, potentially due to chelate cooperativity. However, preliminary evidence also supports the allosteric activation of the DC-SIGN’s CBS via this pocket. In this context, our findings provide important impulses for studying glycan recognition and immune regulation. We further envision that specific, heteromultivalent delivery systems targeting DC-SIGN can be leveraged for next-generation immunotherapies.

## Results

### Discovery of Man-Based Glycomimetics as Langerin Ligands

**Figure 1:**
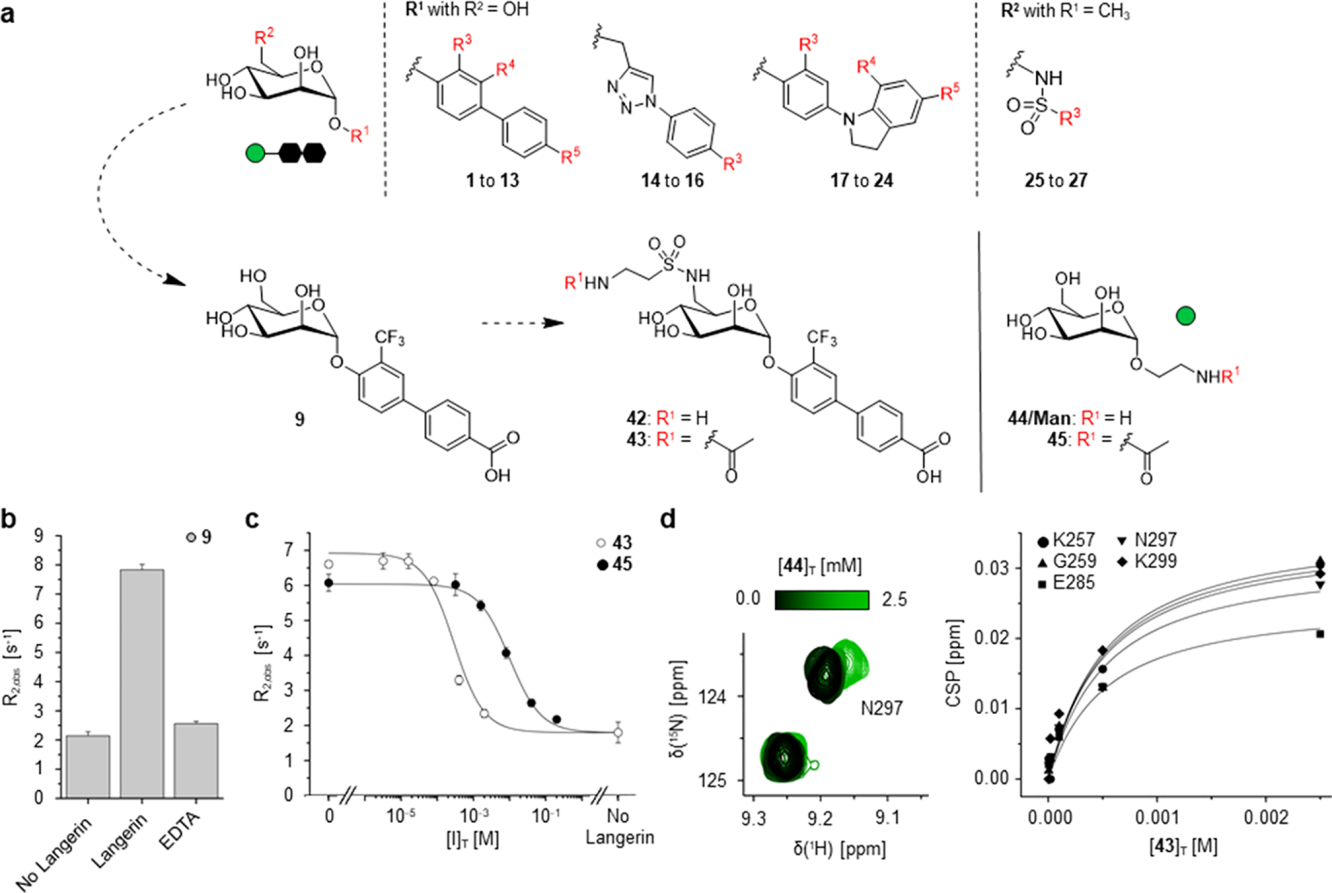
Discovery of Man-based glycomimetics as Langerin ligands. (a) A focused library of 27 mannosides was screened against Langerin in a ^19^F NMR RDA. The library was previously synthesized as FimH and LecB inhibitors for the developments of anti-infectives against *E. coli* and *P. aeruginosa*, respectively.^40−46^ The screening yielded biphenyl aglycone-bearing mannoside **9** as a promising hit that was subsequently modified to **42** to enable conjugation to liposomes via a sulfonamide linker. (b) The Ca^2+^-dependent interaction of **9** with the Langerin CBS was validated by the addition of EDTA in direct ^19^F R_2_-filtered NMR binding experiments using the trifluoro methyl group. (c) The *K*_I_ value determination for acetylated **42** (→ **43**, *K*_I_ = 0.25 ± 0.07 mM) via the ^19^F NMR RDA revealed a 40-fold affinity increase over the **Man** reference **45** (Table S4, *K*_I_ = 10 ± 1 mM). Data shown for **45** were previously published and the mannoside was prepared as previously described.^13^ (d) The affinity of **43** (*K*_D_ = 0.46 ± 0.09 mM) was validated via ^15^N HSQC NMR.

The initial aim of this study was to identify novel glycomimetic ligands for Langerin. To this end, we composed a focused library of mannosides derivatized either in α-orientation of C1 or in C6 (Figures 1a and S1, Tables S1 and S2). These glycomimetics were previously synthesized and originally developed as inhibitors targeting the bacterial lectins FimH and LecB, expressed by *E. coli* and *P. aeruginosa*, respectively.^40−46^ Derivatization in C1 included differentially substituted biphenyl, phenyl-indolinyl, and triazolyl-phenyl systems (**1** to **24**, Table S1) while substituents in C6 were comprised of aromatic and aliphatic sulfonamides (**25** to **27**, Table S2). Screening this focused library against Langerin in a ^19^F NMR reporter displacement assay (RDA) yielded ligands with micromolar affinities, in particular the biphenyl aglycone-bearing mannoside **9** (*K*_I_ = 0.23 ± 0.03 mM and a *K*_D_ = 0.5 ± 0.2 mM) (Figure 1b, Note S1, Figures S2 and S3, Table S3). The introduction of sulfonamides in C6 also resulted in moderate affinity increases over the methylated **Man** reference as observed for **25** (*K*_I_ = 3.0 ± 0.2 mM, *K*_D_ = 2.9 ± 0.4 mM) (Notes S2, Figures S2 and S3, Table S3). To test whether these contributions were additive, we synthesized **42** combining the most potent biphenyl system and a sulfonamide linker amenable for conjugation to liposomes and other nanoparticles (Figure 1a, Figures S4 and S5, Scheme S1). Comprehensive characterization of the interaction between acetylated **42** (i.e., **43**) and Langerin in both ligand- and receptor-observed NMR experiments confirmed a 40-fold affinity increase over **Man** reference **45** (*K*_I_ = 0.25 ± 0.07 mM, *K*_D_ = 0.46 ± 0.09 mM) (Figure 1c and d, Figure S6, Table S4), comparable to previously published heparin-derived targeting ligands.^13^ However, the structure–activity relationship for substituents in C1 and C6 was found to be nonadditive (Tables S3 and S4). Further details on the underlying binding mode of **43** are given in the Supporting Information (Note S3).

### Unexpected Specificity of Liposomes for DC-SIGN^+^ Cells

**Figure 2:**
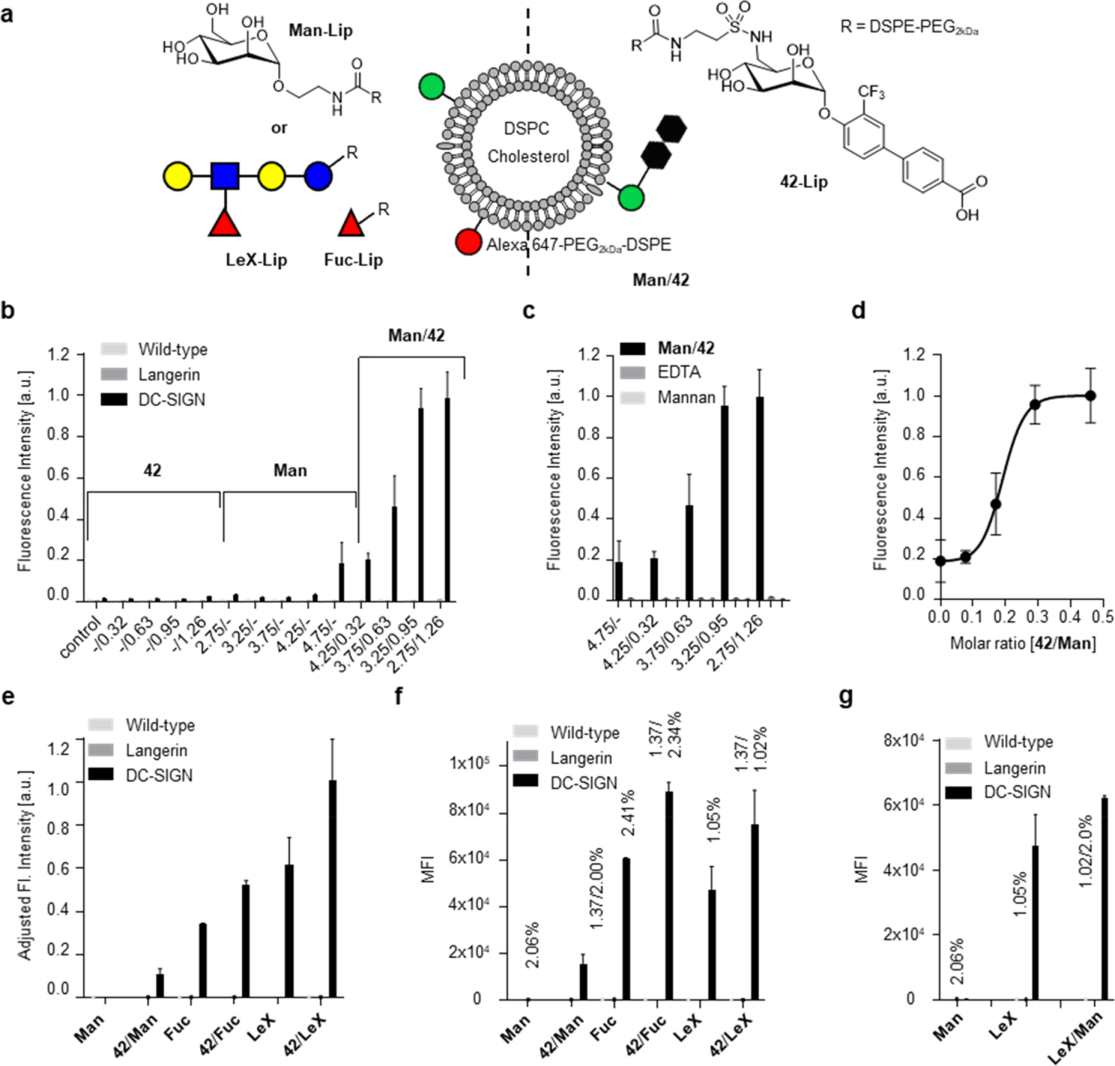
Heteromultivalent liposomes show enhanced binding to DC-SIGN^+^ cells. (a) Schematic depiction of heteromultivalent liposomes. Exemplarily for a composition comprised of **42-Lip** and **Man-Lip** ligands. (b) Binding of homo- and heteromultivalent **Man-Lip**/**42-Lip** liposomes to Raji cells: **42-Lip** up to 1.26% total lipid concentration does not show any binding. **Man-Lip** on its own starts showing binding at 4.75%. The combination of both ligands facilitates strong binding with increasing amounts of **42-Lip** and concurrently decreasing amounts of **Man-Lip**. (c) Binding of **Man-Lip**/**42-Lip** liposomes is inhibited by addition of EDTA or mannan suggesting involvements of Ca^2+^ mediated interaction at the primary binding site. (d) The cooperative binding effect saturates around a molar **42-Lip** /**Man-Lip** ratio of 0.5. (e) Heteromultivalent liposomes using **42-Lip** in conjunction with two other natural DC-SIGN ligands (**Fuc-Lip** and **LeX-Lip**): All plotted data were adjusted to the same natural ligand concentration (2.5%). Due to different coupling efficiencies, the molar ratios for **42-Lip** vary, albeit remaining within the effect saturation regime of above 0.5 (see Figure 2d). (f) Raw data of measurements prior to processing shown in (e). (g) Homo- and heteromultivalent liposomes comprising two natural ligands do not significantly change in their ability to bind DC-SIGN (exemplarily shown for **Man-Lip** and **LeX-Lip**).

Motivated by these findings, we conjugated **42** to DSPE-PEG_2 kDa_ lipids (**42-Lip**) for display on liposomes to explore binding to Langerin^+^ as well as DC-SIGN^+^ and Dectin-1^+^ model cells (Scheme S3). Presumably due to lipophilic interactions with the liposome membrane, at 3 mol % **42-Lip**, reduced liposome stability was observed, accompanied by unspecific binding. While liposomes were stable at 1 mol %, no cell binding was observed. We hypothesized that heteromultivalent liposomes containing **Man-Lip** as a hydrophilic, natural glycan ligand in addition to 1 mol % of **42-Lip** could restore binding to Langerin^+^ cells (Figure 2a and b). However, neither this approach nor the addition of *N*-acetyl-glucosamine (**GlcNAc-Lip**) or **Fuc** (**Fuc-Lip**) was successful (Figures S9 and S10). Co-formulations of **42**-**Lip** with the previously discovered heparin-derived targeting ligand **50-Lip**, by contrast, displayed specific binding to Langerin^+^ cells comparable to homomultivalent liposomes only containing **50-Lip** (Figure S11 and Scheme S4).^13^ These observations are consistent with the micromolar monovalent affinities determined by NMR for both glycomimetics. We conclude that the failure of **42-Lip** to efficiently target Langerin^+^ cells is likely due to its incompatibility with liposomal formulations beyond 1 mol % rather than NMR assay artifacts. Similar to previous reports, the millimolar *K*_D_ values of **GlcNAc-Lip** or **Fuc-Lip** (Scheme S4) are presumably not sufficient to restore liposomal binding.^4,13^

Although unspecific binding was still not fully suppressed at 2 mol % **42-Lip** concentration, we observed increased binding to DC-SIGN^+^ but not Langerin^+^ cells (Figure S12). This specificity for DC-SIGN was further increased for heteromultivalent liposomes containing **Man-Lip** in addition to 1 mol % of **42-Lip** (Figure 2a and b). Intrigued by these unexpected observations, we focused our investigations on the interaction between this class of glycomimetics and DC-SIGN.

### Cooperative Binding to DC-SIGN^+^ Cells upon Copresentation of Natural Glycan Ligands

The systematic analysis of varying ligand ratios confirmed the striking cooperativity for heteromultivalent formulations (Figure 2a). Whereas no binding of homomultivalent liposomes containing **Man-Lip** or **42-Lip** could be observed, binding of heteromultivalent liposomes containing both **42-Lip** and **Man-Lip** increased with the ratio of **42-Lip** over **Man-Lip** and saturated at equimolar amounts (Figure 2d). Furthermore, binding was fully inhibited by both EDTA and the polysaccharide mannan (Figure 2c), confirming the involvement of the Ca^2+^-dependent CBS of DC-SIGN.

These cooperative effects were reproduced with other natural glycan ligands of DC-SIGN, i.e. **Fuc-Lip and LeX-Lip**, albeit with a less pronounced relative increase in liposome binding (Figure 2e and f). To exclude the potential impact of altered spacing or reduced aggregation on the liposome surfaces, we replaced these ligands with **GlcNAc-Lip** serving as a non-DC-SIGN-binding control. This control experiment did not show any cooperativity (Figure S9). We also prepared heteromultivalent liposomes with combinations of the above natural glycan ligands (i.e., **Man-Lip**, **LeX-Lip**, **Fuc-Lip**), in which cooperativity was much less pronounced compared to liposomes containing **42-Lip** (Figure 2g and Figure S10).

Based on the above findings, we hypothesized that **42-Lip** might target a secondary binding pocket on the DC-SIGN surface, in addition to the CBS. The subsequent experiments testing this hypothesis were conducted with **48** (Figure S14, Schemes S2 and S4), a derivative where the sulfonamide linker of **42** was replaced by an amine to reduce synthetic efforts and molecular complexity. The properties of **48-Lip** in the corresponding flow cytometry experiments were found to be comparable to those of **42-Lip** (Figure S13).

Finally, to rule out the involvement of other receptors expressed by Raji cells, we conducted liposome-binding experiments using **48-Lip** on different cell lines (i.e., U937 and THP1 cells)—wild type, DC-SIGN^+^, or Langerin^+^. All cell lines displayed similar binding profiles excluding the possibility of Raji cell-specific phenomena and supporting our hypothesis that the observed cooperativity arises from the interaction of glycomimetics **48** or **42** with DC-SIGN (Figure S15).

### Identification of a Secondary DC-SIGN Binding Pocket

**Figure 3:**
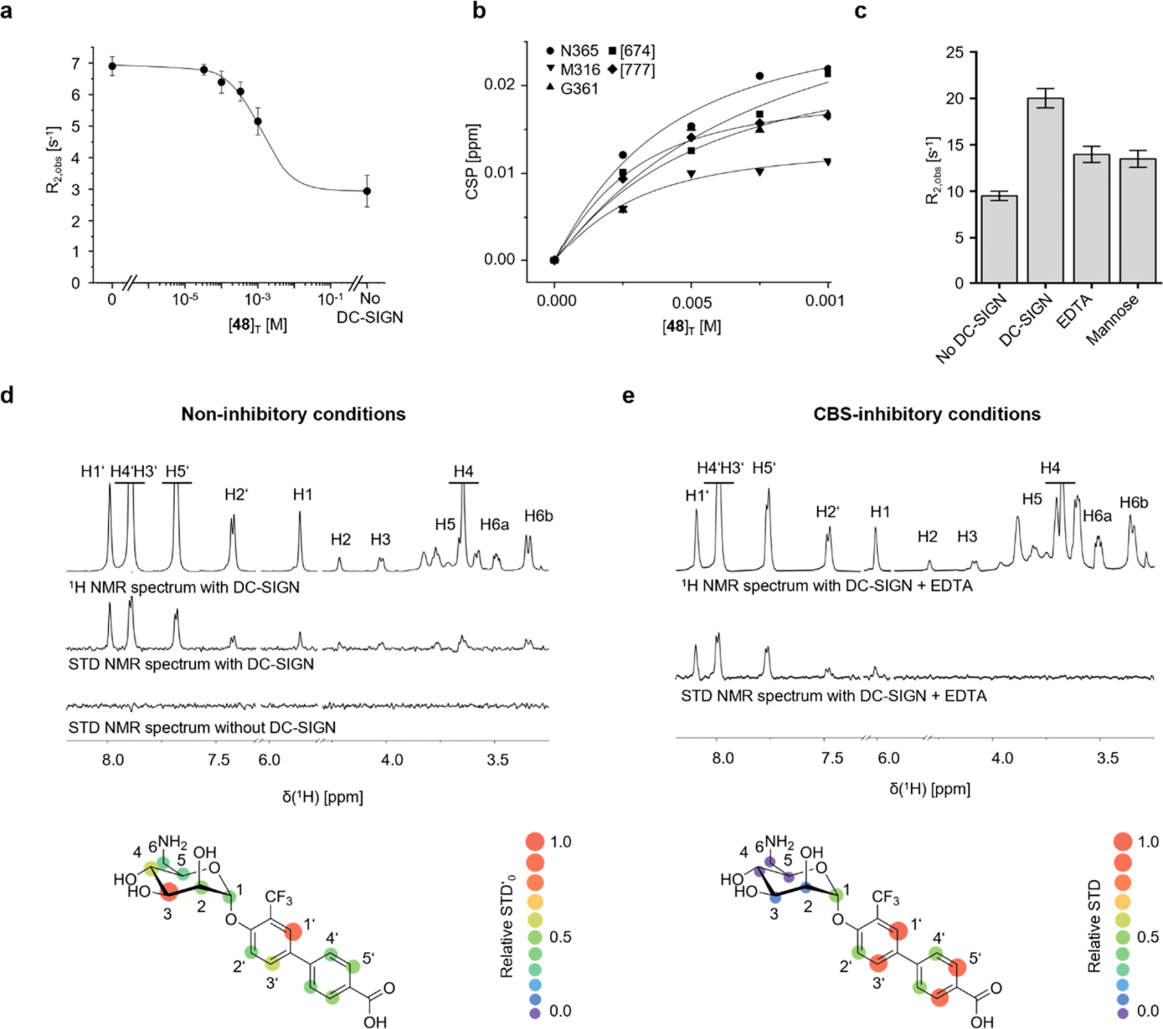
Ligand-observed binding mode analysis for DC-SIGN. (a) Titration experiments using the ^19^F NMR RDA indicated interaction of **48** with DC-SIGNs CBS with affinity in the low millimolar range (*K*_I_ = 1.15 ± 0.01 mM). (b) The *K*_D_ value determined in ^15^N HSQC NMR titrations indicated higher affinity than measured with the ^19^F NMR RDA (*K*_D_,^15^N HSQC = 0.46 ± 0.16 mM). (c) Direct ^19^F R_2_-filtered NMR binding experiments using the trifluoromethyl group of **48** show incomplete inhibition in the presence of 4 mM EDTA or 200 mM mannose, suggesting a secondary Ca^2+^-independent binding mode. (d) STD NMR experiments in the presence of Ca^2+^ served to determine the Ca^2+^-dependent binding mode of **48** with DC-SIGN ECD. Specific STD effects were only observed in the presence of DC-SIGN ECD, allowing for epitopes to be determined from normalized STD′_0_ values calculated from STD build-up curves at *t*_sat_ of 0.5, 1, 2, and 6 s (Figure S18). The STD NMR spectrum is magnified 4-fold. STD′_0_ values in H3 and H4 indicated a mannose-type interaction with DC-SIGN, while a high STD′_0_ in H1′ suggested the −CF_3_ on phenyl ring A to be involved in binding. (e) ^1^H STD NMR experiments in the presence of EDTA-*d*_11_ served to validate the Ca^2+^-independent binding mode with DC-SIGN ECD. Epitopes were obtained from normalized STD values calculated from STD spectra at *t*_sat_ of 2 s and displayed a shift from a mannose-dependent binding to an interaction dominated by the biphenyl-moiety in C1 in the absence of Ca^2+^. The STD spectrum is magnified 4-fold. CBS-inhibition using an excess of mannose revealed similar STD NMR epitopes (Figure S19).

To investigate whether our observations could be explained by the monovalent affinity of **48** or **42** for DC-SIGN, we transferred the ^19^F NMR RDA previously described for the monomeric carbohydrate recognition domain (CRD) of DC-SIGN to the tetrameric extracellular domain (ECD) (Figure S16, Table S5).^13,47^ Although receptor precipitation impeded complete inhibition of the reporter when exceeding 1 mM **48**, titration experiments still allowed the estimation of a millimolar *K*_I_ value (*K*_I_ = 1.15 ± 0.01 mM) supporting interactions with the CBS (Figure 3a). Orthogonal assays using direct quantification of ^19^F CSPs and relaxation rates R_2_ of **48** via its trifluoromethyl group, as well as a ^15^N HSQC NMR titration, revealed micromolar *K*_D_ values in the range determined for Langerin (*K*_D_,^19^F CSP = 0.37 ± 0.06 mM, *K*_D_,^19^F R_2_ filtered = 0.48 ± 0.06 mM, *K*_D_,^15^N HSQC = 0.46 ± 0.16 mM) (Figures 3b and S17, Table S6). To confirm the involvement of the Ca^2+^-dependent CBS, we then performed R_2_-filtered NMR experiments under inhibitory conditions and observed that neither high **Man** concentrations nor EDTA addition completely abrogated binding of **48** to DC-SIGN (Figure 3c). Notably, similar experiments with EDTA and Langerin resulted in complete inhibition of this interaction (Figure 1b and c). Taken together, these observations indicate Ca^2+^-independent, secondary interactions for **48** that are specific for DC-SIGN (Figure 1b and c).

Using STD NMR and ^15^N HSQC NMR experiments in conjunction with molecular docking studies, we explored the suspected dual binding mode in more detail: Initial STD NMR epitope mapping for **48** revealed STD effects for all ligand protons in the presence of DC-SIGN (Figures 3d and S18). This contrasts our findings for Langerin, where **42** showed a binding epitope dominated by saturation transfer to the biphenyl aglycone (Note S3). For DC-SIGN, the highest STD′_0_ values were determined for H3 with substantial STD effects for H4 (Figures 1 and 3d). These results indicate close contact of **48**’s **Man** scaffold in the Ca^2+^-dependent CBS with limited interactions by the biphenyl aglycone, similar to previous STD NMR studies of glycomimetics targeting DC-SIGN.^20,48,49^

In inhibition experiments with EDTA, STD effects were substantially reduced for all **Man** scaffold protons, while saturation transfer for the biphenyl aglycone was sustained (Figure 3e). The addition of deuterated **Man** (**Man-d**_**7**_) enhanced this effect for H1, H2, and H3, albeit residual signals from **Man-d**_**7**_ impeded the quantification of STD effects for H4, H5, and H6 (Figure S19). In conjunction with the ^19^F NMR experiments, these observations strongly support the existence of a secondary binding pocket for **48** where recognition is dominated by the biphenyl system.

**Figure 4:**
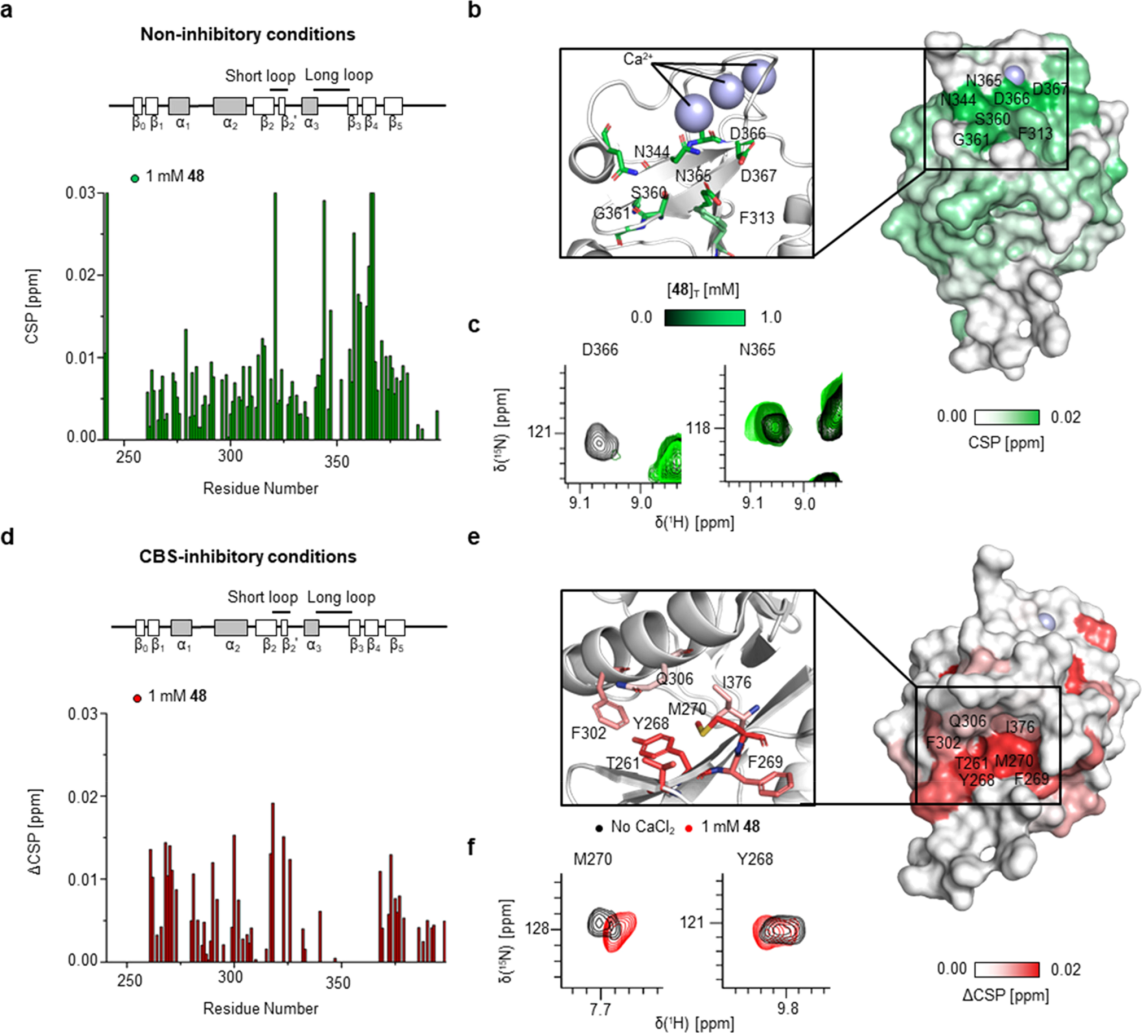
Interaction of **48** with a secondary DC-SIGN binding pocket. (a) CSPs observed in ^15^N HSQC NMR in the presence of Ca^2+^ confirm involvement of CBS residues in binding of **48**. (b) Mapping of CSPs on X-ray structure of DC-SIGN (PDB code: 1SL4) corroborates interaction with the CBS. (c) Examples of CBS residues showing fast exchanging resonances and reduced intensity upon titration (N365 and D366). (d and e) ΔCSPs were determined by subtracting CSPs observed under noninhibitory conditions from those observed under CBS-inhibitory conditions (Figure S25). The CSP map shows increases for residues of the secondary binding pocket as well as in the short and long loop regions in the absence of Ca^2+^. Mapping of ΔCSPs on the X-ray structure of DC-SIGN (PDB code: 1SL4) locates the secondary binding pocket between α helix 2 and β sheet 0 and 1. (f) Examples of residues in the secondary binding pocket showing increased CSPs upon **48** addition (M270, Y268).

In accordance with the results from STD NMR experiments as well as previous studies with **Man** scaffolds, ^15^N HSQC NMR spectra in the presence of Ca^2+^ revealed characteristic CSPs for residues in DC-SIGN’s CBS (N344, N365, E358, N366, N367, S360, and F313) (Figures 4a to c, S20, and S21).^50^ These CSP patterns and trajectories were also found for **Man** titration data (Figure S22). Additionally, **48** induced CSPs remote from the CBS, for residues that have been previously identified by Aretz et al. as part of a set of secondary binding pockets in DC-SIGN (Figure S21).^17^ To verify a direct interaction of **48** with this site, we carried out ^15^N HSQC NMR in Ca^2+^-free buffer and competition experiments with high **Man** concentrations. Under these conditions, CSPs of M270 and Y268 as well as neighboring residues increased significantly, further supporting our secondary binding pocket hypothesis and a shift toward this interaction (Figures 4b to d, S23, S24, and S25).

Interestingly, CSPs outside of this secondary binding pocket, including residues in the long and short loop of the CLR fold (for example K368, G323, T326) were also observed (Figure S25). As for the secondary pocket itself, these remote residues displayed increased CSPs under CBS-inhibitory conditions (Figures 4a and e and S25). We propose these CSP patterns to be the result of allosteric perturbations potentially affecting glycan or Ca^2+^ binding upon ligation at the secondary binding pocket (Figures 4f, S23, S24, and S25).^51,52^ Notably, CSPs for the CBS could not be determined in the absence of Ca^2+^ because the corresponding resonances were not detectable (Figures S23 and S25). We have made this observation previously for Langerin and attribute it to increased conformational dynamics.^36^

### Computational Analysis of Interaction with DC-SIGN’s Secondary Binding Pocket

**Figure 5:**
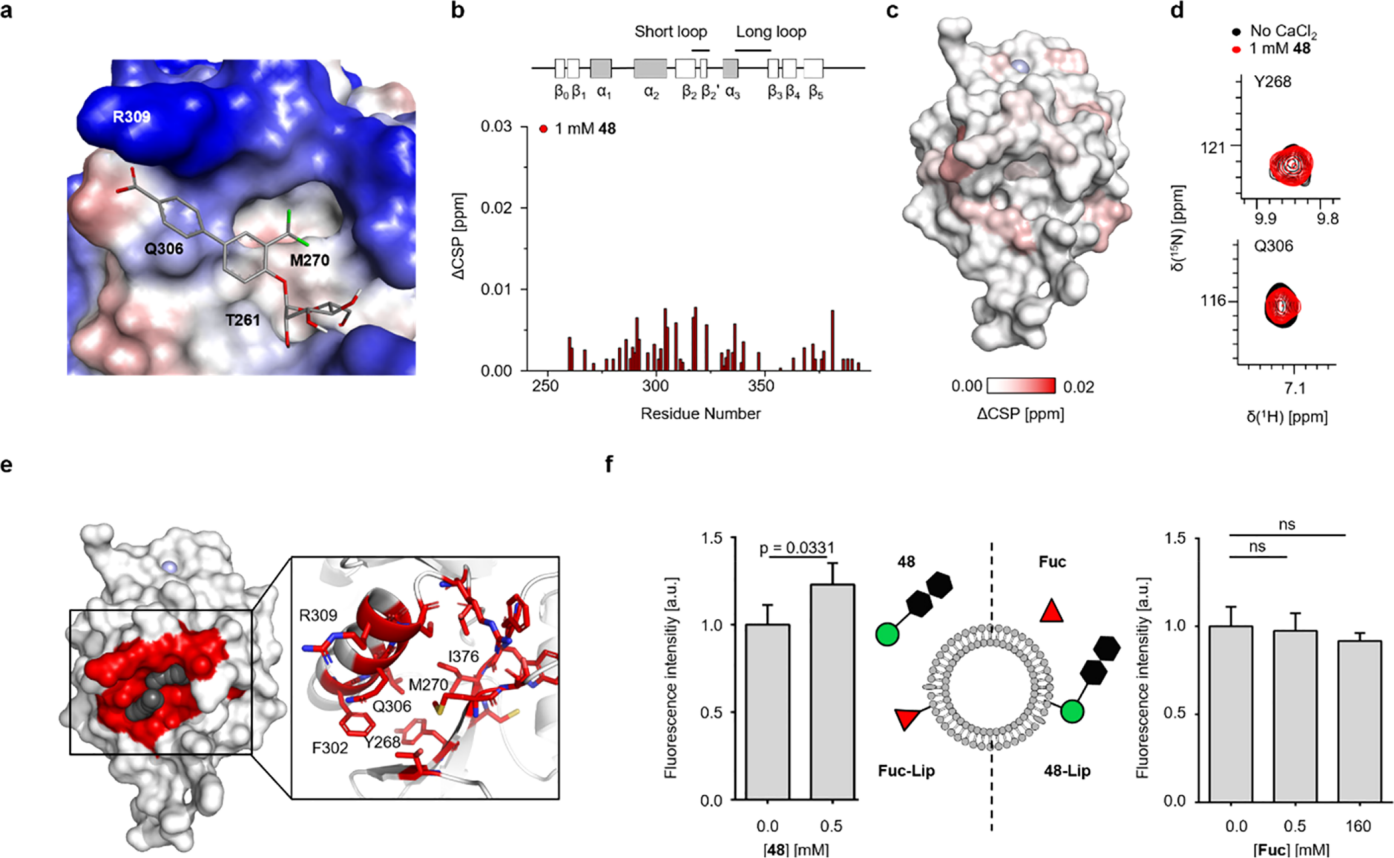
Binding of natural glycan ligands to DC-SIGN is positively modulated by binding of **48**. (a) The best-scored pose obtained from biased molecular docking simulations agrees with our experimental NMR-based binding mode analysis. The receptor surface is colored according to its hydrophobicity. (b and c) ^15^N HSQC NMR experiments with the M270F mutant and **48** demonstrate abrogation of **48**-binding in the absence of Ca^2+^. ΔCSPs were determined by subtracting CSPs observed under noninhibitory conditions from those observed under CBS-inhibitory conditions (Figures S29 and S30). Compared to wild-type DC-SIGN, mapping of ΔCSPs on the X-ray structure of DC-SIGN (PDB code: 1SL4) revealed no increase in CSPs for the secondary binding pocket under inhibitory conditions. (d) Exemplary resonances of residues in the secondary binding pocket (Y268 and Q306) demonstrate abrogation of **48**-binding. (e) The allosteric binding site predicted by AlloSite shares residues with the herein identified secondary binding pocket. A pseudoligand is depicted in black spheres. (f) DC-SIGN^+^ Raji cells were incubated with AF647-functionalized liposomes carrying either 2.5 mol % **Fuc-Lip** as a CBS ligand (left panel) or 1.26 mol % **48** (right panel) in conjunction with soluble **48** or **Fuc**, respectively. MFIs were normalized to samples containing no **48** or **Fuc** and then averaged over the results from four biological replicates, each conducted as two technical replicates. Soluble **48** significantly enhances binding of **Fuc-Lip** liposomes to Raji DC-SIGN^+^ cells (*p < 0.05; *n* = 4; two-tailed, unpaired Student’s *t* test). In contrast, soluble **Fuc** did not increase binding of **48** liposomes to DC-SIGN^+^ Raji cells (*p* > 0.05; *n* = 4; two-tailed, unpaired Student’s *t* test) (lipophilic, red; hydrophilic, blue).

To further analyze the interaction of **48** at the identified secondary binding pocket, we performed molecular docking simulations. Using the X-ray structure of DC-SIGN (PDB code: 1SL4), we leveraged the observed CSPs as spatial restraints for the conformational search.^53,54^ The best-scored docking pose shows hydrophobic interactions between the biphenyl system and M270 (Figure 5a). The model also predicts ionic interactions of the R309 side chain and the carboxylate of **48**. We then refined the model using molecular dynamics simulations revealing the complex to be stable during the 20 ns simulation time scale (Figure S26). This allowed for the identification of transient interactions between the hydroxyl groups of the **Man** scaffold of **48** and surrounding residues in the proposed secondary binding pocket.

Next, we set out to validate the proposed binding mode using site-directed mutagenesis. We selected M270 since it is the closest amino acid to the trifluoromethyl group and additionally shows the highest CSP in the secondary binding pocket. The M270F mutant of DC-SIGN was selected, since it shows the lowest impact on global protein stability based on the FoldX score while also enabling steric exclusion of the aglycone (Figure 5a, Table S9).^55^ The mutant was expressed, and ^15^N HSQC NMR experiments served to confirm folding and monodispersity (Figures S27 and S28). In analogy to the wild-type protein, we conducted binding experiments with **48** under noninhibitory and CBS-inhibitory conditions. While binding of **48** to the CBS was retained under noninhibitory conditions, the absence of Ca^2+^ led to a global decrease in ΔCSPs (Figures 5b and c, S29, and S30). In particular, CSPs for residues that are part of the secondary binding pocket such as Y268 and Q306 were fully abrogated (Figure 5d). In summary, we conclude that **48** interacts with DC-SIGN both via the CBS and a remote, secondary binding pocket (Figure 4e). This newly discovered pocket is mainly formed by residues Y268, M270, F302, Q306, R309, and I376. Based on both NMR experiments and computational studies, we propose a binding mode that is dominated by the biphenyl aglycone (Figure 5a). The existence of additional binding pockets for the aglycone results in chelation-mediated avidity enhancement and likely contributes to the cooperative binding of heteromultivalent liposomes to DC-SIGN^+^ cells.

### Potential Allosteric Activation of Glycan Recognition by DC-SIGN

We then raised the question whether allostery, beyond avidity enhancement due to additional binding pockets for the aglycone, might also contribute to the striking cooperativity observed with **48**-containing heteromultivalent liposomes when targeting DC-SIGN^+^ cells. This hypothesis was initially supported by ^15^N HSQC NMR CSPs induced remotely of the secondary binding pocket, even under CBS-inhibitory conditions, as discussed above (Figures 4d and S25). To substantiate these observations, we employed AllositePro, a computational tool predicting allosteric pockets based on general structural features of allosteric proteins and probable dynamic changes upon ligand binding.^56,57^ Using the X-ray structure of DC-SIGN complexed with a **Man**-type oligosaccharide (PDB code: 1SL4), two candidate regions were suggested, one of which shared residues with the secondary binding pocket of **48**. This binding pocket was also predicted to have a significant impact on the protein structure upon ligation, indicative of an allosteric site (Figures 5e and S31, Table S6). To test whether these predictions would translate to improved multivalent targeting, we coincubated DC-SIGN^+^ cells with soluble biphenyl mannoside **48** and homomultivalent liposomes containing **Fuc-Lip** or with soluble **Fuc** and homomultivalent liposomes containing **48-Lip**. In accordance with our hypothesis, the presence of 0.5 mM **48** significantly increased binding of **Fuc-Lip** liposomes, suggesting allosteric activation of the CBS (Figure 5f). Conversely, we did not observe enhanced targeting of **48**-presenting liposomes in the presence of **Fuc**, even at high concentrations of the latter. Notably, these conditions did not result in significant inhibition of binding either.

In summary, we conclude that the designed targeting ligand **48** displays promiscuous binding, interacting with both the CBS and a remote, secondary binding pocket. This secondary binding pocket (Figure 4e) mainly binds the biphenyl aglycone with minor involvement of the **Man** moiety (Figure 5a). Consequently, this newly discovered pocket would allow for enhanced chelation of individual DC-SIGN tetramers by both homo- and heteromultivalent liposomes. This results in an increased avidity, hence explaining the selective targeting of DC-SIGN^+^ cells over Langerin^+^ cells. Moreover, our investigations provide evidence for an additional allosteric contribution that directly, or indirectly, enhances glycan recognition by DC-SIGN.

## Discussion

Fueled by advances in glycobiology, CLRs have emerged as therapeutically relevant targets over the past decade.^23,58−64^ Especially glycomimetics selectively binding Langerin and DC-SIGN offer promising opportunities for novel DC immunotherapies and anti-infectives.^20^ In this study, we started with a screening campaign to discover ligands for Langerin by employing a focused library of **Man** derivatives, previously synthesized as inhibitors of the bacterial lectins FimH and LecB.^40−46^ Here, we identified mannoside **42** bearing a biphenyl aglycone with micromolar affinity for Langerin, comparable to previously developed targeting ligands.^13^**42** was conjugated to lipids allowing for its multivalent presentation on liposomes. Using this approach proved essential for validating potential lead molecules in a physiologically relevant context. Although ligand-functionalized liposomes are typically stable up to at least 5 mol % DSPE-PEG_2 kDa_ lipids, we observed destabilization at 2 mol % of **42-Lip**, likely due to the ligand’s hydrophobicity.^65^ Hence, the glycomimetic failed to provide efficient and specific targeting of Langerin^+^ cells.

While troubleshooting liposomal formulations with **42-Lip**, we were intrigued by another rather unexpected finding: In DC-SIGN^+^ control cells, stable liposomes comprised of 3.75 mol % **Man-Lip** and only 0.36 mol % **42-Lip** showed strong and highly selective binding. Those heteromultivalent liposomes were superior to homomultivalent liposomes containing 4.75 mol % **Man-Lip**, a known ligand for DC-SIGN. This cooperative effect was reproduced by liposomes containing **42-Lip** and other natural DC-SIGN ligands (i.e., **Fuc-Lip** and **LeX-Lip**) but was absent for the nonbinding control with **GlcNAc-Lip**. We can thus exclude this phenomenon to originate simply from an increased lipid bilayer stabilization or preventing **42-Lip** aggregation.

The millimolar affinity of the proxy ligand **48** for DC-SIGN as determined by the ^19^F NMR RDA is comparable to **LeX**.^50,66^ Thus, monovalent affinities for the CBS do not explain the effective avidity increase observed in cell-binding experiments with heteromultivalent liposomes presenting a combination of these ligands. Further affinity characterization for **48** in several direct-binding NMR assays also afforded micromolar apparent *K*_D_ values, about 3-fold lower than the determined *K*_I_ and comparable to the affinity for Langerin. One possible explanation for these discrepancies is the existence of a potential secondary binding pocket. NMR experiments under inhibitory conditions with respect to the CBS revealed that binding to DC-SIGN was not fully abrogated, neither in the absence of Ca^2+^ nor in the presence of **Man**. This observation was accompanied by a shift in the STD NMR epitope, now being dominated by the biphenyl aglycone of **48** with only minor contributions the **Man** scaffold. Additionally, CSPs in residues remote from the CBS were enhanced under these conditions. We mapped out the location of a secondary pocket close to residues M270 and Y268 and further characterized the corresponding binding mode of **48** using a combination of site-directed mutagenesis, ^15^N HSQC NMR experiments, and molecular modeling (Figures 4e and 5a). This analysis confirmed the essential contributions of the aglycone to binding. Notably, the involved residues were also identified as part of a potential secondary, Ca^2+^-independent binding pocket as the result of a previously reported fragment screen.^17^ The validated fragment hit from this screen shares a comparable scaffold geometry with the aglycone of **48**, albeit displaying a different electronic structure.

The lower affinity limit of **48** for the secondary pocket can be estimated by adjusting the binding site concentrations in the fitting procedures for *K*_D_ value determination (i.e., accounting for four CBS and four secondary pockets per tetrameric DC-SIGN ECD). Even under this assumption, apparent *K*_D_ values remain lower than the millimolar *K*_I_ (data not shown). In contrast to experiments with Langerin, we observed substantial precipitation of DC-SIGN at **48** concentrations of more than 1 mM, coinciding with the determined affinity for the CBS, likely originating from cross-linking of CRDs or ECDs. Similar phenomena have been observed for glycomimetics designed by Fieschi et al.^67^

Based on the above considerations, we conclude that heteromultivalent liposomes simultaneously present **Man-Lip**, or **Fuc-Lip** and **LeX-Lip**, to the CBS of DC-SIGN and **48-Lip** to the secondary binding pocket, substantially enhancing the avidity compared to homomultivalent formulations and thereby contributing to the efficient targeting of DC-SIGN^+^ cells. The seemingly contradictory inhibition of binding by EDTA can be explained by an avidity threshold, which we have observed before for Langerin with **Man-Lip**-containing liposomes at around 4.5 mol %.^13^ Throughout our investigations, multiple observations further suggested the additional involvement of allostery in heteromultivalent liposome binding: (1) the striking extent of cooperativity at relatively low monovalent affinities of **48** for both the CBS and the secondary pocket; (2) structural rearrangements in the short and long loop regions adjacent to the CBS (for example, K368, G323, T326), upon **48** binding to the secondary pocket in NMR experiments; (3) the computational characterization of the secondary binding pocket as an allosteric site with a high probability for structural rearrangements upon engagement; and, most importantly, (4) the increased binding of homomultivalent liposomes presenting **Fuc-Lip** in the presence of soluble **48**.

**Figure 6:**
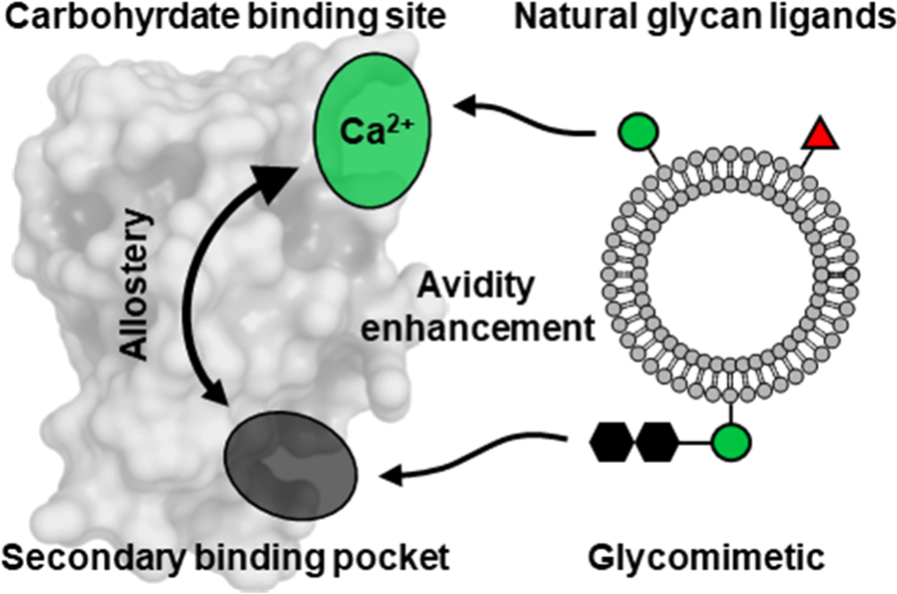
Proposed mechanism of heteromultivalent avidity enhancement for DC-SIGN. Binding of a secondary binding pocket-ligand (i.e., **42**/**48**) causes structural rearrangements in the DC-SIGN CRD affecting the carbohydrate binding site. This allosteric activation might increase Ca^2+^ complexation or directly natural glycan ligand binding, resulting in cooperative avidity enhancement for the heteromultivalent liposome beyond the targeting of two independent binding sites.

Hence, we propose a mechanism where allosteric activation increases the affinity of DC-SIGN for its natural glycan ligands, triggered by **48**-binding to a secondary, hydrophobic pocket located around M270 and adjacent residues (Figure 6). This is supported by several studies observing allostery in CLRs:^68^ (1) the characterization of a Ca^2+^-dependent allosteric network in Langerin,^36,37^ (2) the discovery of allosteric inhibitors for the murine ortholog of Langerin,^38^ (3) the identification of secondary binding pockets for DC-SIGN,^17^ and (4) cumulative evidence from various RDA-based fragment screens against both Langerin and DC-SIGN indicated enhanced binding to the glycan-based reporter in the presence of fragments or fragment mixtures.^17,31,47,69^ We further conclude that the cooperative effects in cell-binding experiments observed for heteromultivalent liposomes might be driven by a combination of both chelation-derived avidity enhancement and allosteric activation of the CBS.

In summary, our study provides the first mechanistic evidence for allosteric activation of DC-SIGN and lectins in general. Yet, several aspects of the interaction with the discovered glycomimetics warrant further investigation. Prominently, when considering the reciprocal nature of allostery, glycan binding should in turn increase the affinity of **48** at the secondary pocket.^70−72^ We were not able to demonstrate functional reciprocity for **48-Lip**-displaying liposomes in the presence of soluble **Fuc** (Figure 5f). Structurally, the differential binding epitope of **48** at the secondary binding pocket, depending on the mode of inhibition (i.e., with **Man** versus Ca^2+^ depletion), could indicate allosteric communication induced at the accessory Ca^2+^ sites (Figures 3e and S19). Notwithstanding, reductionist experiments using biphenyl-type, nonglycan ligands are required to independently determine affinities at CBS and the secondary binding pocket, thereby quantifying the proposed allosteric activation of DC-SIGN. This will further aid the elucidation of the relative contributions of allostery and avidity enhancement to heteromultivalent liposome targeting.

The substantiation of the proposed mechanism would have broad impact on the field of glycoscience. Allosteric activators could be exploited to strengthen CLR-mediated cell–matrix or cell–cell interactions with implications for cancer cell metastasis, immune cell extravasation, and pathogen recognition. We thus envision this concept to be a valuable addition to the toolbox of chemical glycobiology. Finally, our results also highlight the vast potential of fragment-based drug design—especially for targeting secondary binding pockets with nanoparticle-based delivery systems and on “undruggable” receptors such as CLRs, as explored before.^17,38,39,69^ The approach not only increases the effective number of available binding sites but also statistically favors specificity—both paramount to overcoming low affinity, promiscuous glycan recognition.

## Supporting Information Available

Supporting Notes S1–S3, Figures S1–S31, Tables S1–S10, Schemes S1–S4, and detailed description of experimental methods (PDF)
