# Supplementary material for: A Remote Secondary Binding Pocket Promotes Heteromultivalent Targeting of DC-SIGN
Source: J Am Chem Soc. 2021 Nov 8;143(45):18977–88. doi: 10.1021/jacs.1c07235 (PMC8603350; doi:10.1021/jacs.1c07235)
Supplement: Supplementary file 1 — ja1c07235_si_001.pdf [file ja1c07235_si_001.pdf]

Supplementary Information

A Remote Secondary Binding Pocket Promotes Heteromultivalent Targeting of DC-SIGN

Robert Wawrzinek[§1], Eike-Christian Wamhoff[§1,2], Jonathan Lefebre[§1,2], Mareike Rentzsch[1,2], Gunnar Bachem[3], Gary Domeniconi[3], Jessica Schulze[1,2], Felix F. Fuchsberger[1,2], Hengxi Zhang[1,2], Carlos Modenutti[4], Lennart Schnirch[1,2], Marcelo A. Marti[4], Oliver Schwardt[5], Maria Bräutigam[1], Mónica Guberman[1], Dirk Hauck[6,7], Peter H. Seeberger[1,2], Oliver Seitz[3], Alexander Titz[6,7,8], Beat Ernst[5], Christoph Rademacher[1,2,9,10]*

[1]Department of Biomolecular Systems, Max Planck Institute of Colloids and Interfaces, 14424 Potsdam, Germany.

[2]Department of Chemistry and Biochemistry, Freie University of Berlin, 14195 Berlin, Germany

[3]Department of Chemistry, Humboldt University of Berlin, 12489 Berlin, Germany

[4]Departamento de Química Biológica e IQUIBICEN-CONICET, Universidad de Buenos Aires, C1428EHA Ciudad de Buenos Aires, Argentina

[5]Department of Pharmaceutical Sciences, University of Basel, 4056 Basel, Switzerland

[6]Chemical Biology of Carbohydrates, Helmholtz Institute for Pharmaceutical Research Saarland, Helmholtz Centre for Infection Research, 66123 Saarbrücken, Germany

[7]German Centre for Infection Research, Campus Hannover-Braunschweig, 38124 Braunschweig, Germany

[8]Department of Chemistry, Saarland University, 66123 Saarbrücken, Germany

[9]University of Vienna, Department of Pharmaceutical Sciences, Althanstrasse 14, 1090 Vienna, Austria;

[10]University of Vienna, Department of Microbiology, Immunology and Genetics, Max F. Perutz Labs, Biocenter 5, 1030 Vienna, Austria;

[§]These authors contributed equally to this work

**Email: christoph.rademacher@univie.ac.at*

Keywords: C-type lectins, allostery, targeted delivery, glycans, multivalency

Contents

Supporting Notes 3

Note S1 – Structure activity relationship in C1 – Langerin 3

Note S2 – Structure activity relationship in C6 – Langerin 4

Supporting Figures 5

Supporting Tables 30

Supporting Schemes 40

Methods 43

Synthetic chemistry - procedures 43

Synthetic chemistry – ^{1}H and ^{13}C NMR Spectra 57

Liposome preparation 71

Cell culture 72

Liposome binding assay – flow cytometry 72

Receptor expression and purification 73

NMR binding experiments – Langerin 75

^{19}F R_2-filtered NMR 75

STD NMR 75

^{15}N HSQC NMR 76

NMR binding experiments – DC-SIGN 78

General Remarks 78

^{19}F R_2-filtered NMR 78

K_D determination from ^{19}F NMR CSPs 79

STD NMR 80

^{15}N HSQC NMR 80

Molecular docking 81

MD simulations 82

Webserver-based allosteric site prediction 83

Supporting References 84

Supporting Notes

Note S1 – Structure activity relationship in C1 – Langerin

Overall, two subsets of mannosides bearing substituents in C1 displayed promising SARs (Table S1). On the one hand, the previously reported affinity increase for biphenyl substituents was validated using the ^{19}F NMR RDA.[1] On the other hand, phenyl-indolinyl substituents displayed comparable estimated K_I values.

The evaluation of the SAR obtained for the biphenyl subset of the mannoside library revealed an unexpected trend. **9** ($K_{I,est}$ = 0.2 mM) displayed the highest affinity within this subset. This derivative bears a trifluoromethyl group in ortho position of the proximal phenyl ring and a carboxyl group in para position of the distal phenyl ring. Strikingly, the introduction of the trifluoromethyl group accounted for a 45-fold affinity increase over reference molecule **1** ($K_{I,est}$ = 9 mM). A comparable affinity was obtained for **3** ($K_{I,est}$ = 0.3 mM) for which the trifluoromethyl group is substituted by a chloride group. This affinity increase was neither observed for **2** ($K_{I,est}$ = 8 mM) which bears the chloride group in meta position of the proximal phenyl nor for other substituents in ortho position. Moreover, carboxyl ester or carboxamide formation for the carboxyl group resulted in decreased affinities as exemplified by **4** ($K_{I,est}$ = 8 mM) and **10** ($K_{I,est}$ = 2 mM). Interestingly, the comparison of estimated K_I values for **1**, **3**, **5** ($K_{I,est}$ = 2 mM) and **6** ($K_{I,est}$ = 4 mM) indicated that the contributions of substituents in ortho position of the proximal phenyl ring and in para position of the distal phenyl ring are not independent of each other. Substitutions in both positions have been proposed to influence the dihedral angles determining the relative orientation of the two phenyl rings and the **Man** scaffold, resulting in a non-additive SAR.[2] Hence, the optimal combination of substituents is likely required to optimally orient the two phenyl rings for the formation of favorable interactions with the Langerin surface. Notably, conformational entropy and solvation effects might also contribute to the observed SAR. The latter have been reported to be of particular importance for carbohydrate-protein interactions.[3] Yet, both contributions are difficult to quantify experimentally.

As the phenyl-indolinyl subset of the focused library comprised fewer mannosides, the obtained SAR information was limited. Yet, the evaluation of estimated K_I values revealed an additional class of potent glycomimetic Langerin ligands, complementing the findings for the biphenyl subset. In similarity to the latter, all derivatives displaying submillimolar affinities are substituted with a chloride group in the ortho position of the proximal phenyl ring. Within this subset, **17** ($K_{I,est}$ = 0.4 mM), **20** ($K_{I,est}$ = 0.3 mM) and **22** ($K_{I,est}$ = 0.3 mM) displayed the lowest estimated K_I values. The indolinyl ring of these glycomimetics is substituted with methyl and chloride groups in different positions. Interestingly, polar substituents on the indolinyl ring did not seem to be required as exemplified by the decreased affinities of **19** ($K_{I,est}$ = 0.7 mM) and **21** ($K_{I,est}$ = 1 mM). This suggests the formation of interactions which differ from those observed for the biphenyl subset. Alternatively, the recognition of both subsets might be dominated by

interactions formed by the aromatic substituents *e.g. via* a potential cation-π bond with K313.

The estimated K_I values for selected mannosides were subsequently reproduced in ^{19}F R_2-filtered and ^{15}N HSQC NMR titration experiments (Figure S2 and S3, Table S3). Overall, the determined affinities were consistent with the screening results and validate glycomimetics either biphenyl or phenyl-indolinyl substituents in C1 of the **Man** scaffold as potent Langerin ligands.

Note S2 – Structure activity relationship in C6 – Langerin

The ^{19}F NMR RDA also served to estimate K_I values for mannosides bearing sulfonamide substituents in C6 (Table S2). Here, the SAR is dominated by the affinity decrease associated with mannoside formation as exemplified by **46** (K_I = 13±3 mM) (Figures S2, Table S3). Compared to this reference molecule, all three screened derivatives displayed a 6.0-fold affinity increase. While sulfonamide groups appear to represent suitable linkers in C6, no affinity increase was observed upon the introduction of phenyl rings for **26** ($K_{I,est}$ = 2 mM) and **27** ($K_{I,est}$ = 2 mM). Finally, the estimated K_I value for **25** was subsequently validated in ^{19}F R_2-filtered and ^{15}N HSQC NMR titration experiments (Figure S2 and S3, Table S3).

Note S3 – Binding mode analysis for mannoside 43 – Langerin

We evaluated the binding mode of **43**: 15N HSQC NMR confirmed interactions with the CBS and revealed unique chemical shift perturbations (CSPs) for D312 compared to the CSP pattern previously reported for **Man** reference **45**, indicating favorable interactions by the biphenyl system (Figure S1b).[4] These findings were corroborated by STD NMR experiments yielding uniformly strong STD effects for the substituent in C1 and suggesting a solvent exposed orientation of the sulfonamide linker (Figures S1c, S7 and S8). These observations are in accordance with binding modes obtained from tethered molecular docking, predicting the formation of a hydrogen bond between the carboxyl group of **43** and N292 (Figure S1d). Additionally, the distal phenyl ring is located near to P310 and the trifluoromethyl group forms van der Waals interactions with A289. Both residues could not be assigned due the structural flexibility of the long loop but are found in proximity of D312. Finally, the sulfonamide linker is oriented towards G284, compatible with conjugation of **43** to liposomes.

Supporting Figures

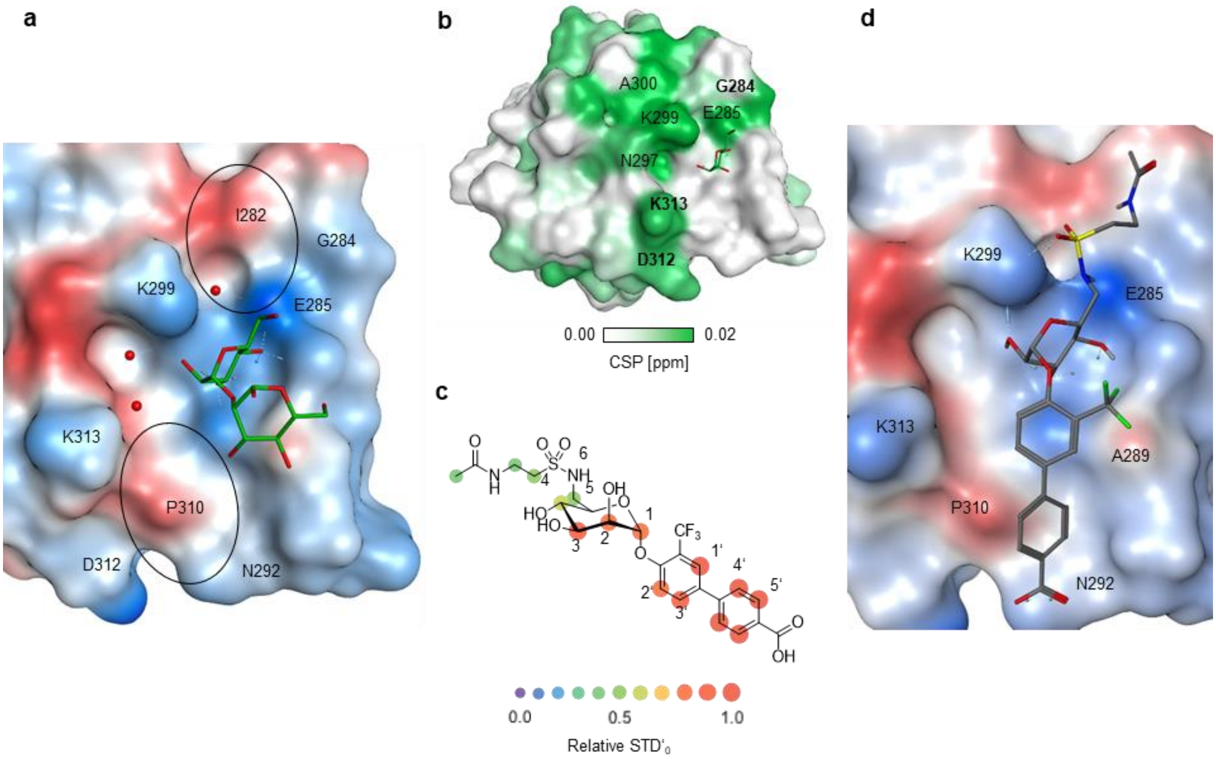

Figure S1: Binding more analysis for mannoside 43 with Langerin. (a). *The binding mode of* ***Man*** *(PDB code: 3P5F) suggests a region of the receptor surface in proximity to K313 potentially targeted by derivatives* ***1*** *to* ***24*** *and a region in proximity to K299 potentially targeted by derivatives* ***25*** *to* ***27****.* ***(b)*** *The mapping of CSPs on the Langerin surface further confirmed interactions of* ***43*** *with the CBS, highlighted by the CSPs observed for E285, N297 and K299. Shown is the X-ray structure of Langerin in complex with* ***Man*** *(PDB code: 3p5f). Compared to reference* ***45****,* ***43*** *induced unique CSPs for D312 and G284, likely due to interactions formed by the substituents in C1 and C6.* ***(c)*** *The STD NMR epitope of* ***43*** *is dominated by strong STD effects for the biphenyl system, indicating the formation of favorable interactions. By contrast, the sulfonamide linker appears to be oriented toward the solvent.* ***(d)*** *Poses obtained from tethered molecular docking studies agree with the NMR-based binding mode analysis and support the formation of a hydrogen bond between the carboxyl group of* ***43*** *and N292, which is in proximity to D312. Additional hydrophobic interactions were predicted between the outer phenyl ring and P310 and the trifluoromethyl group and A289. The sulfonamide linker displays high solvent exposure. The receptor surface is colored according to its lipophilicity (lipophilic: red, hydrophilic: blue).*

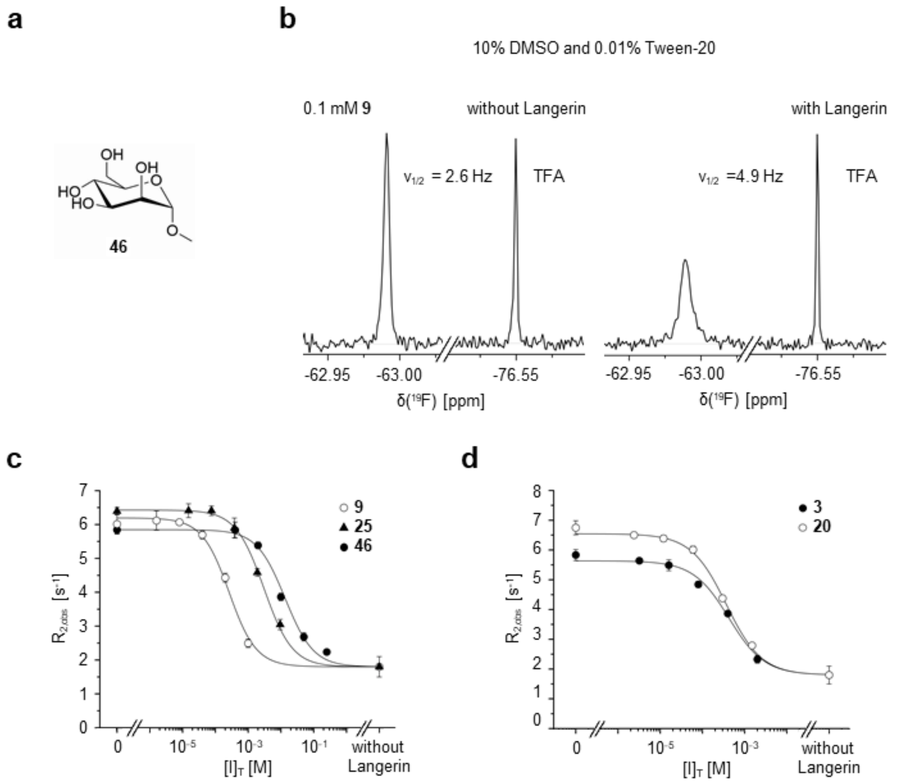

Figure S2: ^{19}F R2-filtered NMR affinity validation for mannosides 3, 9, 20 and 25: (a) *The structure of* ***Man*** *reference* ***46*** *is depicted.* ***(b)*** *^{19}F NMR spectra of* ***9*** *with the additives 10% DMSO and 0.01% Tween-20 are shown. The trifluoromethyl of the ligand displayed line broadening $\Delta\nu_{0.5}$ in presence of the Langerin ECD supporting a specific, aggregation-independent interaction.* ***(c) and (d)*** *Competitive binding experiments in presence of 10% DMSO were conducted to validate the estimated KI values obtained from the ^{19}F R2-filtered NMR screening.* ***46*** *(K_I = 13±3 mM) served as the reference molecule to quantify the affinity increase observed for glycomimetic Langerin ligands.* ***9*** *(K_I = 0.23±0.03 mM) displayed the highest affinity increase, followed by* ***20*** *(K_I = 0.33±0.02) and* ***3*** *(K_I = 0.39±0.05 mM). While these mannosides bear biphenyl or phenyl-indolinyl aglycones, the estimated affinity increase for* ***25*** *(K_I = 3.0±0.2 mM) bearing a sulfonamide substituent in C6 could be validated as well.*

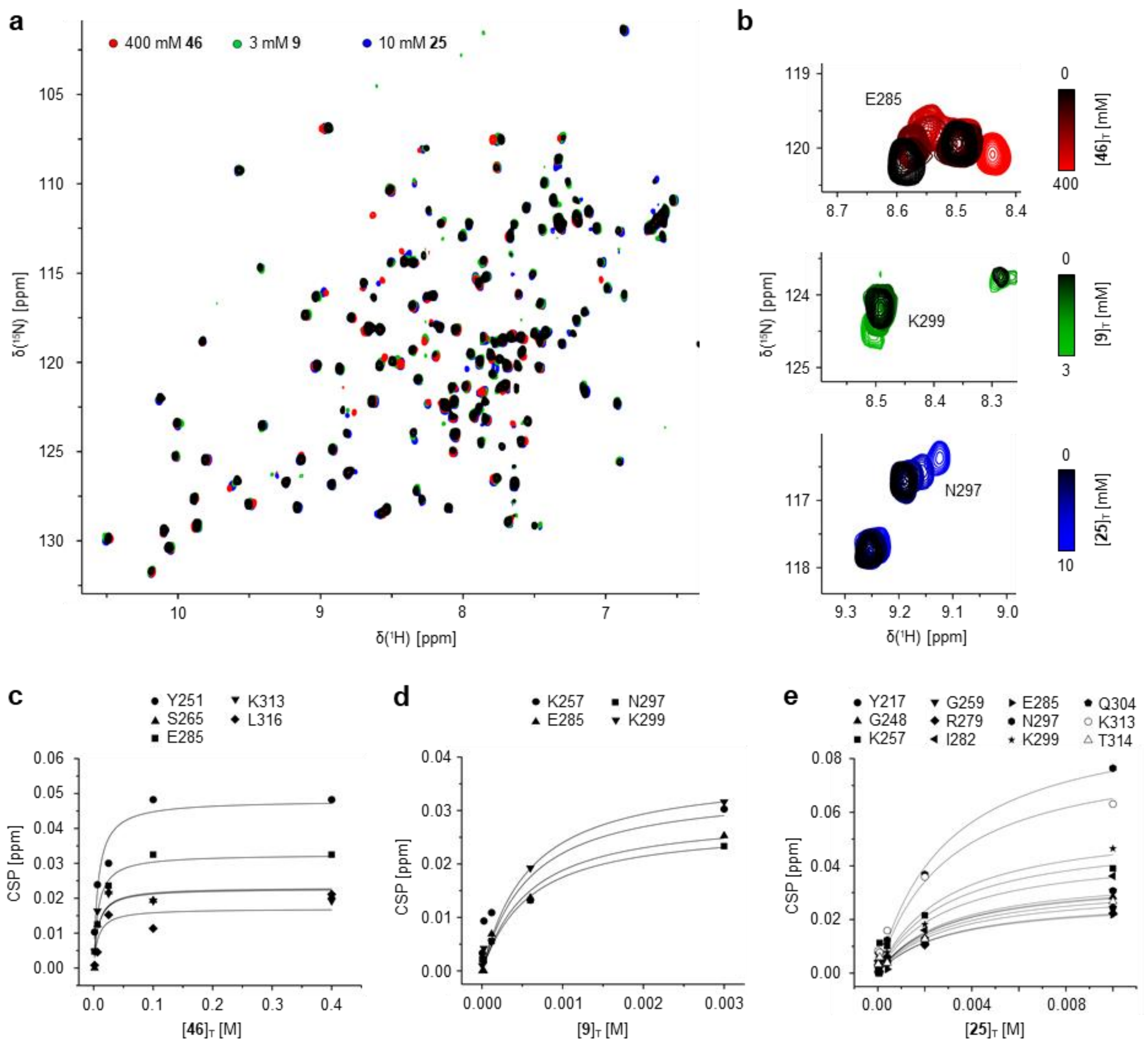

Figure S3: K_D determination for mannosides 9, 25 and 47*: *(a) *Affinities determined for **9**, **25** and **47** utilizing the ^{19}F R_2-filtered NMR assay were validated via ^{15}N HSQC NMR titration experiments with the Langerin CRD in presence of 10% DMSO. (**b)** Assigned resonances displaying fast chemical exchange and CSPs larger than 0.02 ppm were selected for the determination of K_D values. Representative CSP trajectories are depicted. (**c) to (e).** Aside from minor deviations, the K_D values obtained for **47** (K_D = 7±2 mM), **9** (K_D = 0.5±0.2 mM) and **25** (K_D = 2.9±0.4 mM) are consistent with the results from the ^{19}F R_2-filtered NMR assay and validate **9** as a potent glycomimetic Langerin ligand.*

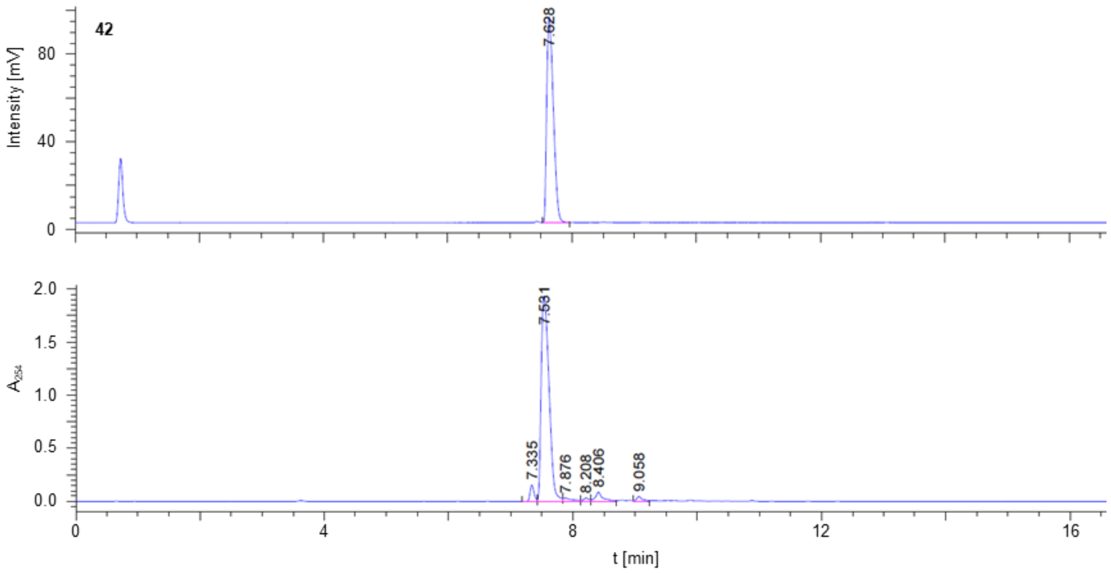

Figure S4: Analytical HPLC trace for mannoside 42. *The purity of* ***42*** *was determined to be >95% via analytical reversed-phase HPLC utilizing an H_2O: acetonitrile gradient (5% in acetonitrile for 10 min and 5% to 95% in acetonitrile in 15 min). Both solvents contained 0.01% TFA. The analysis was conducted on an Atlantis T3 column (Waters) at a flow rate of 0.5 mL·min^{-1} and the elution of the derivative was detected via ELSD or absorbance A_{254} measurements at 254 nm.*

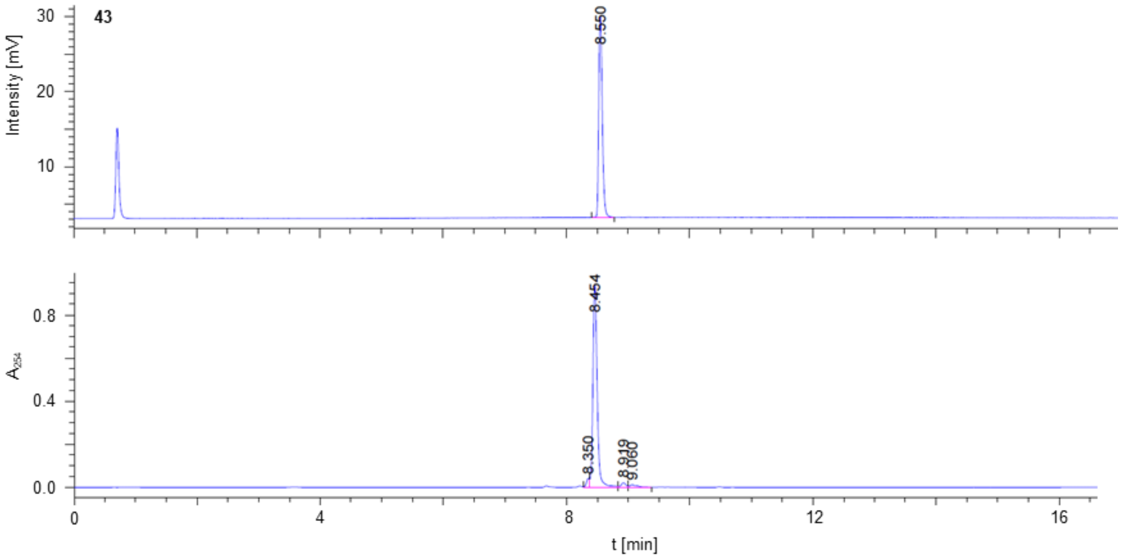

Figure S5: Analytical HPLC trace for mannoside 43. *The purity* ***43*** *was determined to be >95% via analytical reversed-phase HPLC utilizing an H_2O: acetonitrile gradient (5% in acetonitrile for 10 min and 5% to 95% in acetonitrile in 15 min). Both solvents contained 0.01% TFA. The analysis was conducted on an Atlantis T3 column (Waters) at a flow rate of 0.5 mL·min^{-1} and the elution of the derivative was detected via ELSD or absorbance A_{254} measurements at 254 nm.*

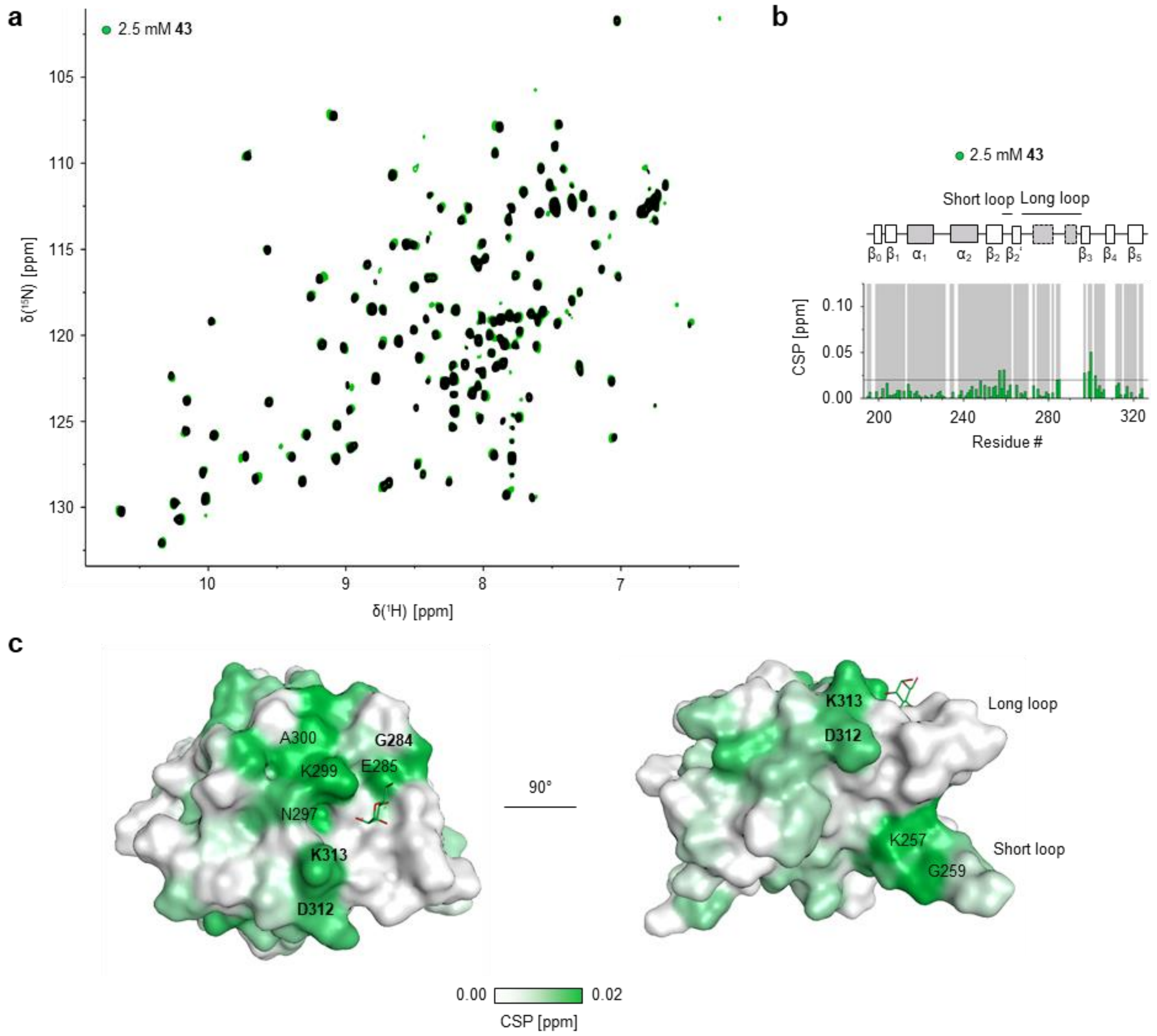

Figure S6: Supplementary ^{15}N HSQC NMR data for interaction between mannoside 43 and Langerin. (a and b) *^{15}N HSQC NMR spectra with the Langerin CRD reveal the CSP fingerprints for* ***43****. Assigned resonances detected in the reference spectrum are highlighted (grey). Observed CSP values for* ***43*** *are likely reduced compared to interaction with previously analyzed monosaccharide or glycomimetic ligands due to the presence of 10% DMSO and intermediate and slow exchange phenomena.*[4] ***(c)*** *Mapping the CSPs on the X-ray structure of the Langerin CRD (PDB code: 3P5F) validates a Ca^{2+}-dependent binding mode as indicated by CSPs observed for E285 and K299.*[5] *Additionally, CSPs are observed for N297, K313 and A300, residues also affected upon recognition of* ***Man*** *and N-acetyl-mannosamine.*[6] *Two distinct features are observed for* ***43*** *compared to the recognition of natural glycan ligands. Here, prominent effects observed for D312 might be indicative for an interaction with the second phenyl ring or the carboxyl group while the CSP for G284 might be inducted by either the sulfonamide group or the acetylated ethylamino linker. These findings suggest that the binding mode of the* ***Man*** *scaffold is maintained. CSPs are also observed in remote regions of the CTL domain fold, particularly for K257 and G259 in the short loop region. This indicates a modulation of the previously reported allosteric network.*[7]

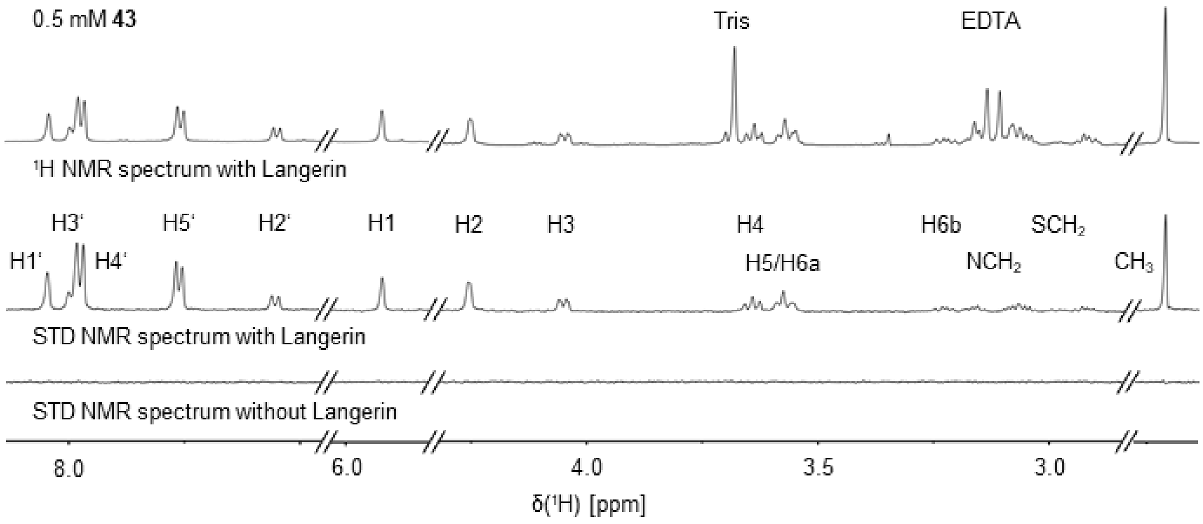

Figure S7: Supplementary STD NMR data for interaction between mannoside 43 and Langerin. *STD NMR experiments were conducted with the Langerin ECD. STD NMR spectra were recorded at saturation times t_{sat} of 0.4 s and are magnified 64-fold.*

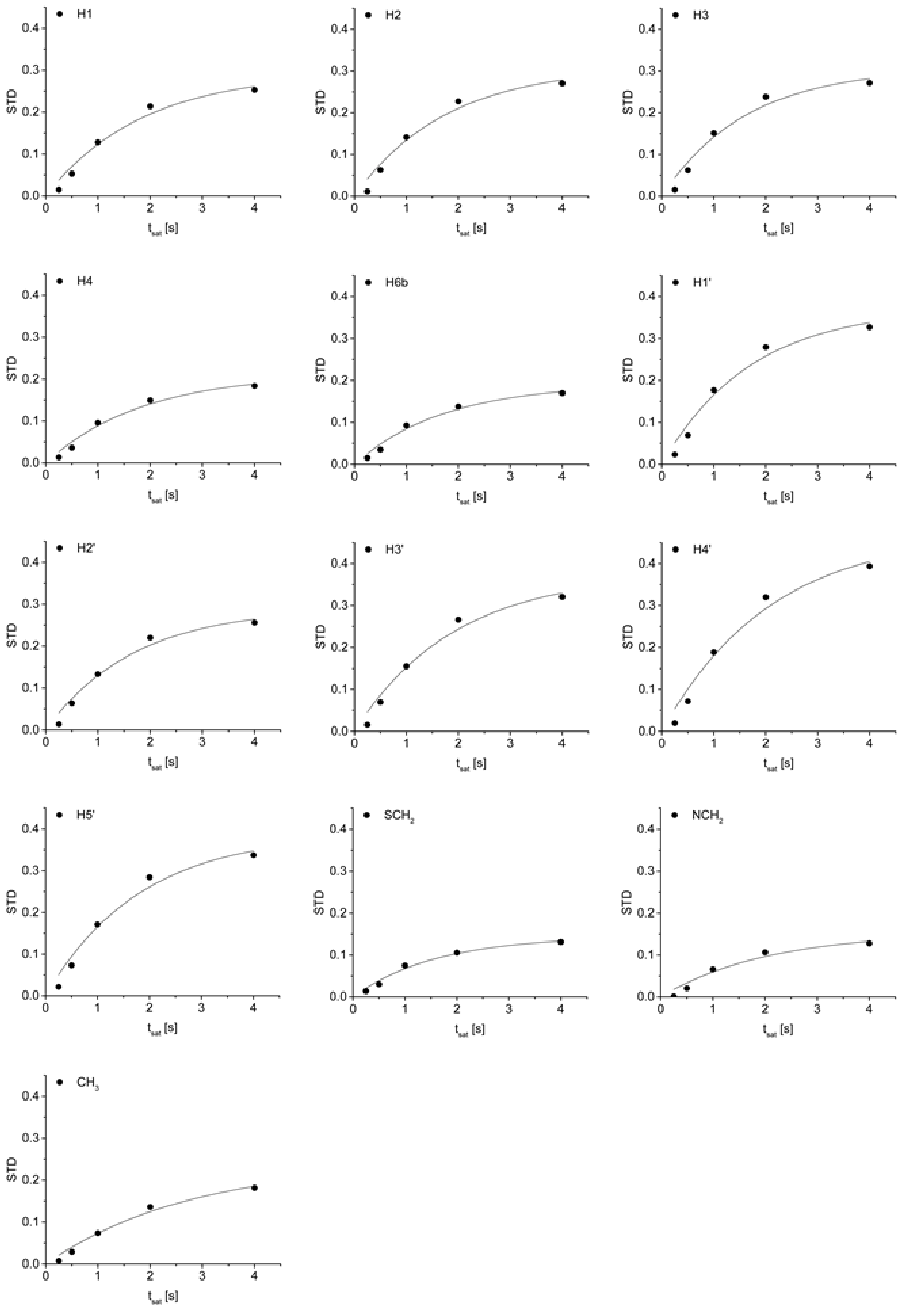

***Figure S8: STD NMR build-up curves for mannoside 43 with Langerin Equation 2** was fitted to STD values to calculate STD_0' values for the determination of the binding epitope of **43**.*

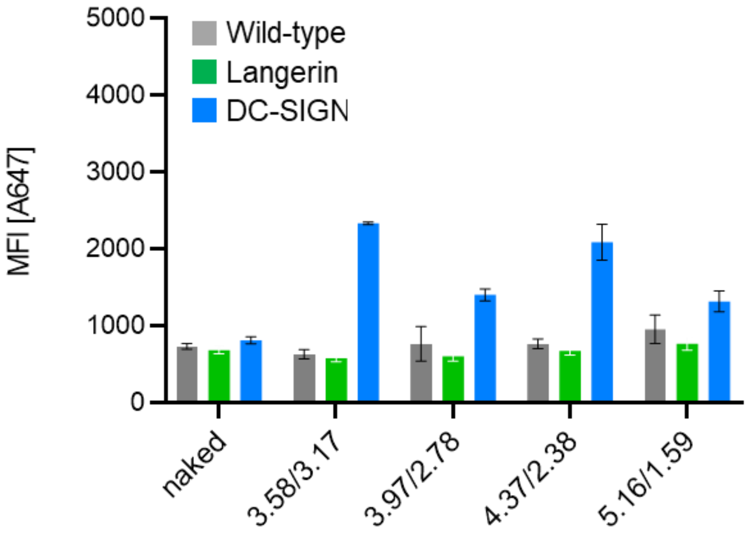

***Figure S9: Heteromultivalent GlcNAc/42 liposomes neither show cooperativity effects nor specific binding towards DC-SIGN**. Binding of heteromultivalent liposomes to CLR-expressing Raji cells was measured in flow cytometry experiments using **42** in conjunction with GlcNAc to control for the dependency of the observed cooperativity and specificity on natural DC-SIGN ligands.*

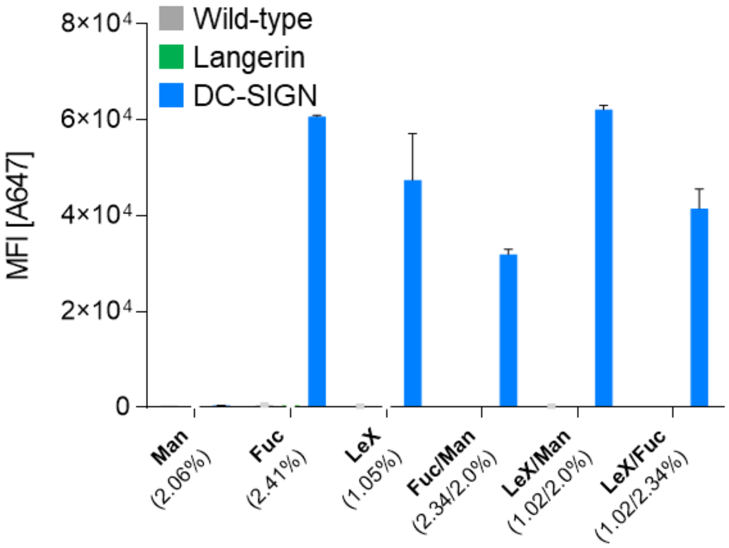

***Figure S10: Heteromultivalent liposomes of natural ligands do not show cooperativity effects in DC-SIGN binding**. Binding of heteromultivalent liposomes to CLR-expressing Raji cells was measured in flow cytometry experiments using natural glycan ligands of DC-SIGN to control for the dependency of the observed cooperativity and specificity on **42**.*

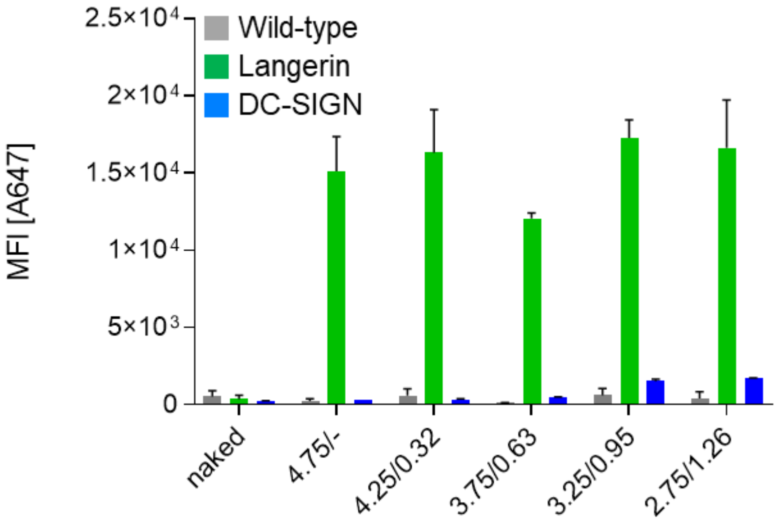

Figure S11: Heteromultivalent 50/48 liposomes bind to Langerin-expressing Raji cells. *Selective binding to Langerin-expressing Raji cells is maintained upon substitution of the previously reported targeting ligand* ***50*** *with mannoside* ***48*** *in flow cytometry experiments* ***(Scheme S4)****.*[4] *This observation is consistent with the comparable K_I and K_D values determined for these glycomimetics by NMR.*

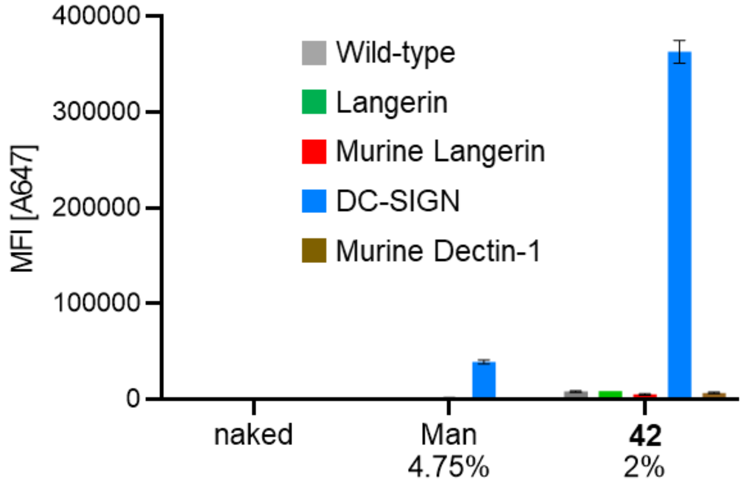

Figure S12: Homomultivalent high-content 42 liposomes interaction with different CLRs. *Homomultivalent liposomes decorated with 2 mol%* ***42*** *to CLR-expressing Raji cells show binding preference towards DC-SIGN in flow cytometry experiments. Binding to other CLRs (Langerin, murine Langerin, and murine Dectin-1) was negligible. Notably, utilized liposomal formulations were not long-term stable and displayed unspecific binding.*

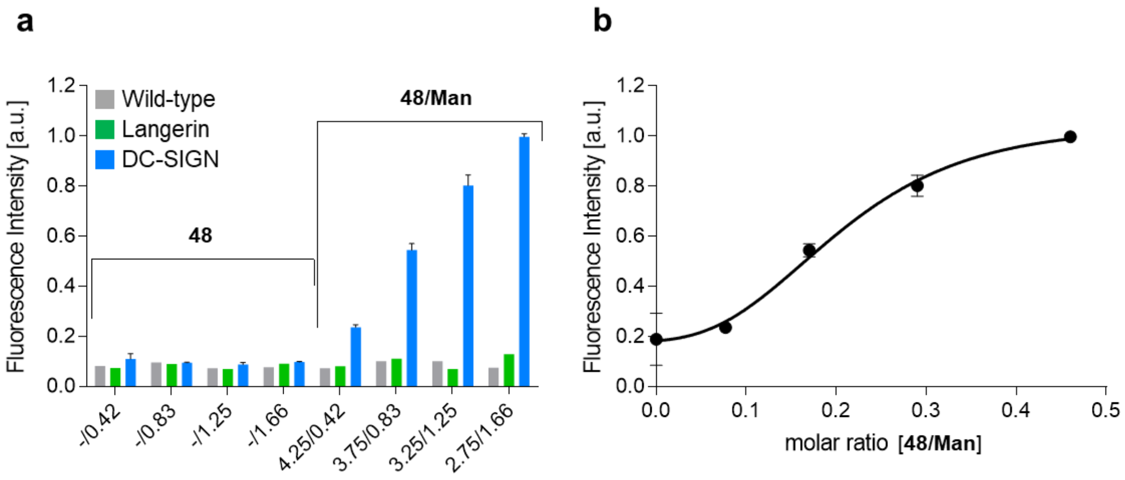

***Figure S13: 48 and 42 are similarly behaving ligands** (compare with Figure 2b). Binding of heteromultivalent liposomes to CLR-expressing Raji cells was measured in flow cytometry experiments using mannoside **48** in conjunction with **Man**. **(a) 48** up to 1.66% total lipid concentration does not show any binding. In conjunction with **Man**, strong binding was observed with increasing amounts of **48** and concurrently decreasing amounts of **Man**. **(b)** The cooperative binding effect saturates around a molar **48**/**Man** ratio of 0.5.*

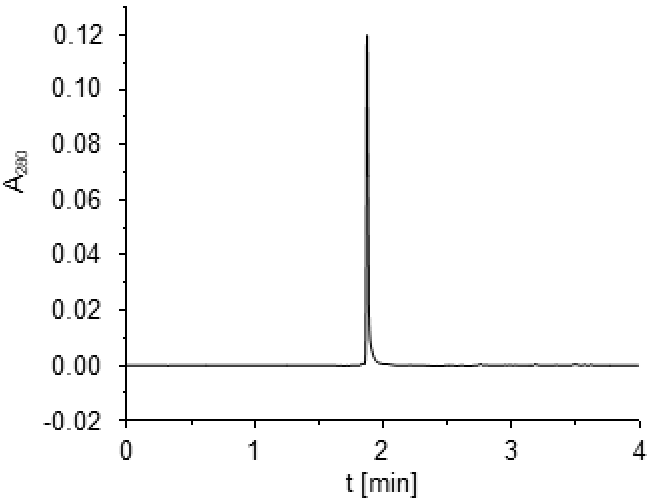

***Figure S14: Analytical HPLC trace for mannoside 48.** The purity of **48** was determined to be >95% via analytical reversed-phase HPLC utilizing an H_2O: acetonitrile gradient (3% to 90% acetonitrile in 4 min). The analysis was conducted on an BEH C18 column (Waters) at a flow rate of 0.5 mL·min^{-1} and the elution of the derivantive was detected via absorbance A_{254} measurements at 280 nm.*

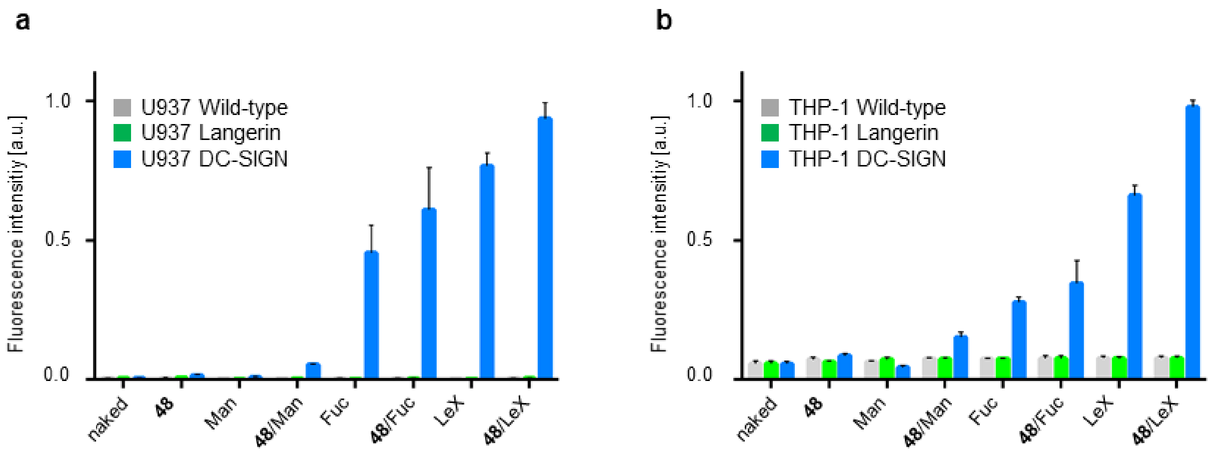

Figure S15: U937 and THP-1 cells overexpressing DC-SIGN or Langerin show the same liposome binding behavior as Raji cells. *Binding of heteromultivalent liposomes in flow cytometry experiments using* ***48*** *in conjunction with natural DC-SIGN ligands (****Man****,* ***Fuc*** *and* ***LeX****) to CLR⁺ U937 and THP-1 cells. All plotted data were adjusted to the same natural ligand concentration (2.5 mol%). Due to different coupling efficiencies the molar ratios for* ***48*** *vary, albeit remaining within the effect saturation regime of above 0.5 (see Figure 2b)****.*** *Similar to DC-SIGN-expressing Raji cells, U937* ***(a)*** *and THP-1* ***(b)*** *DC-SIGN-expressing cells show specific interaction with heteromultivalent liposomes in flow cytometry experiments.*

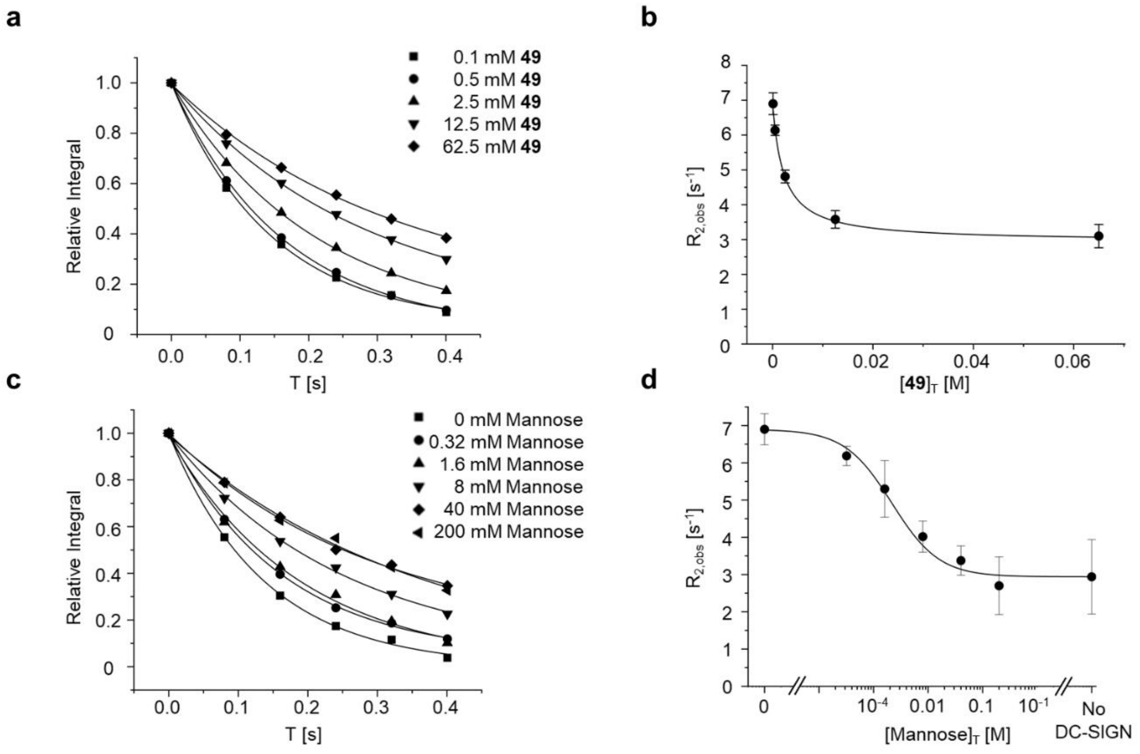

Figure S16: ¹⁹F NMR reporter displacement assay transfer to DC-SIGN ECD. *The interaction between the reporter molecule* ***49*** *and DC-SIGN ECD can be quantified using a CPMG*[5,6] *pulse sequence via the transversal relaxation rate* $R_{2,obs}$ *of the* CF_3 *group. Resulting decay curves for titration points are shown in* ***(a)****.* ***(b)*** *The fitting procedure yields a* K_D *of 2.1 ± 0.2 mM.* ***c)*** *and* ***d)*** *Competitive binding titrations with* ***Man*** *yielded* K_i *values that are in line with literature values (* K_I *= 2.4 ± 0.8mM) .*[6,8,9] *Determined parameters used for the fitting procedure are listed in* ***Table S5.***

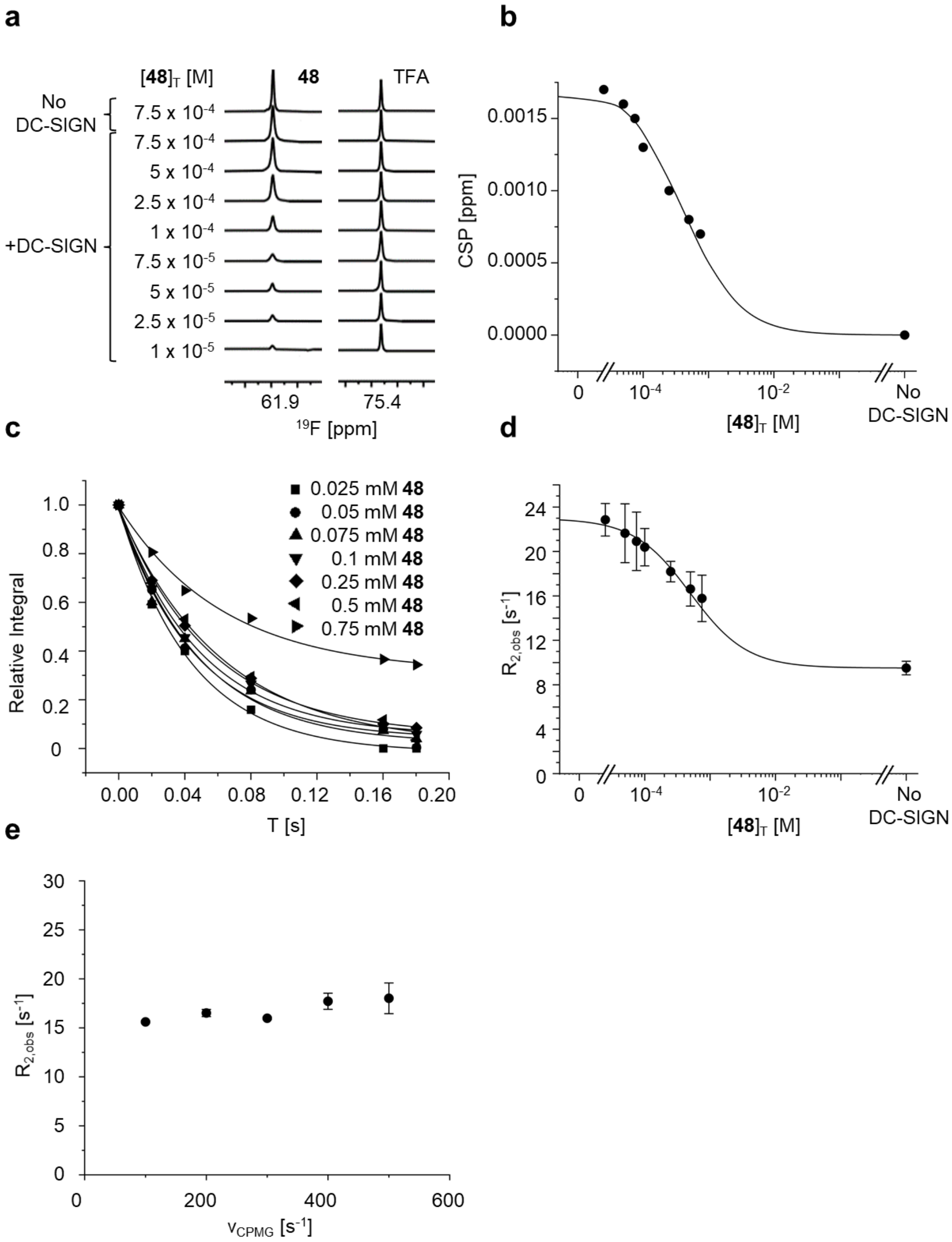

Figure S17: Orthogonal ***^{19}F NMR affinity assays via direct observation of the CF_3 resonance of 48. (a)*** *The ^{19}F NMR resonance of* ***48*** *shifts in the presence of DC-SIGN ECD in a concentration-dependent manner.* ***(b)*** *Fitting of CSPs calculated from spectra shown in* ***(a)*** *yield a K_D of 0.5 ± 0.2 mM.* ***(c)*** *The interaction between* ***48*** *and DC-SIGN ECD can be quantified using the CPMG pulse sequence via the transversal relaxation rate $R_{2,obs}$ of the -CF_3 group (**Figure S16**). Resulting decay curves for titration points are shown in* ***(c)****.* ***(d)*** *The fitting procedure yields a K_D of 0.5 ± 0.1 mM. Determined parameters used for the fitting procedure are listed in* ***Table S5.***

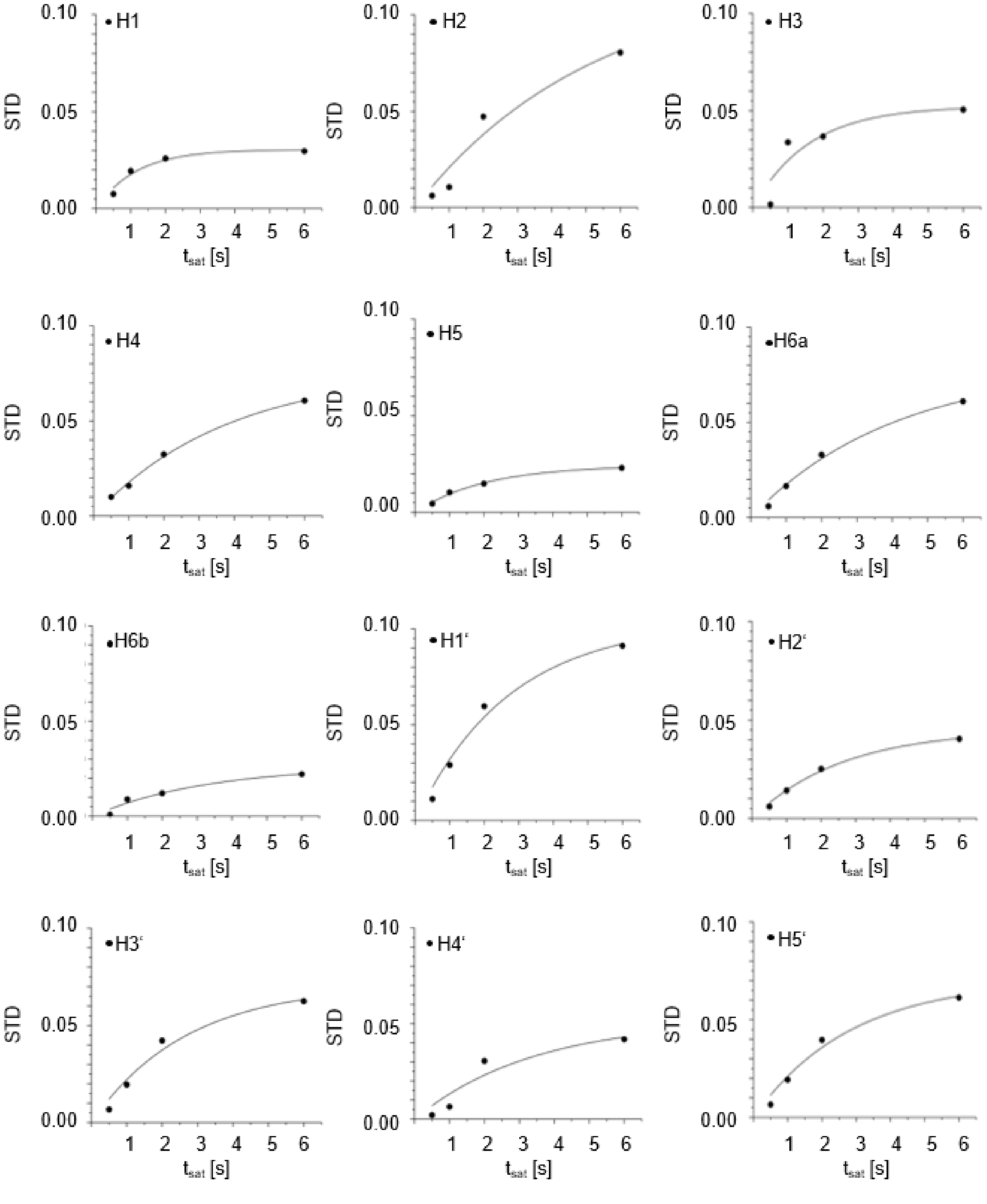

Figure S18: STD NMR build-up curves for 48 without Man or EDTA. Equation 2 *was fitted to STD values allowing determination of STD₀' values. Normalized STD₀' values were used to derive the binding epitope of* ***48*** *(****Figure 3d****).*

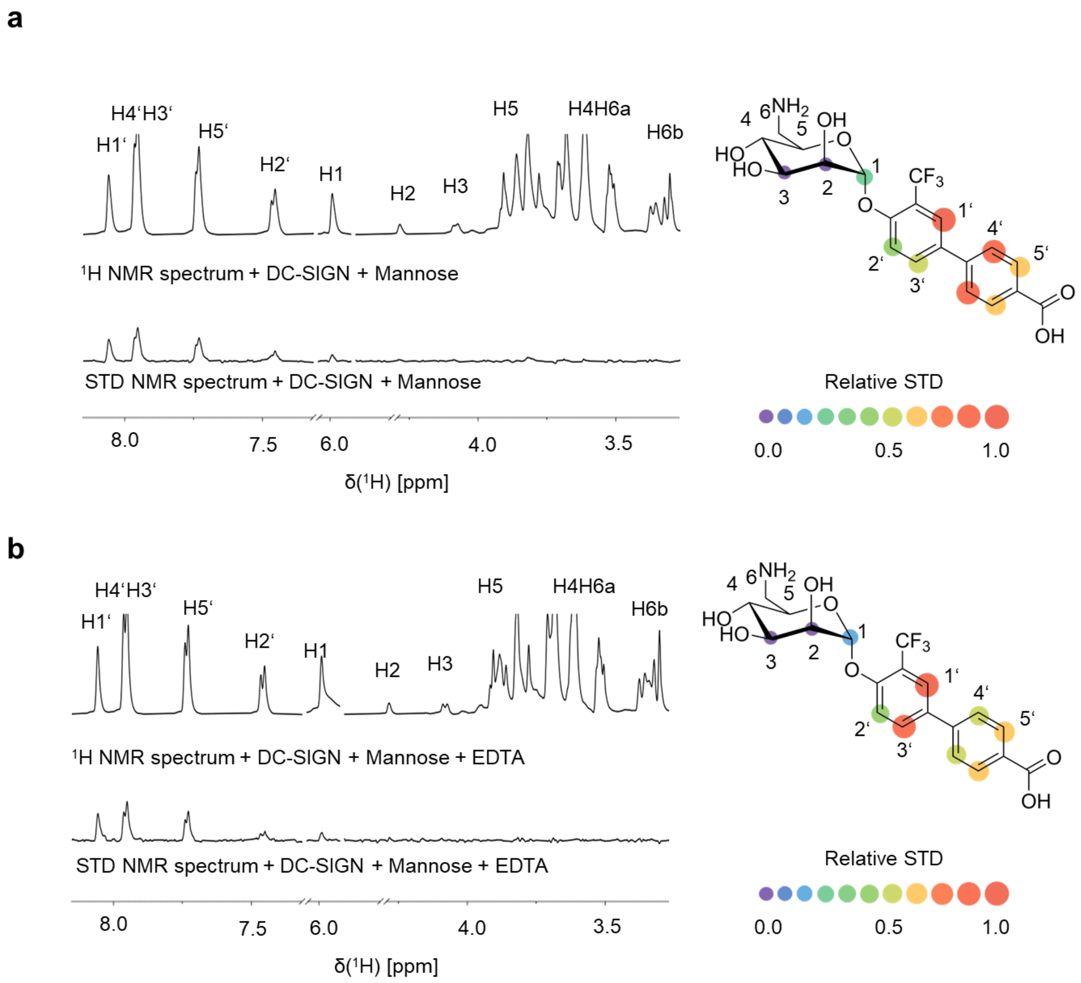

Figure S19: STD NMR epitope mapping for 48 under inhibitory conditions. *STD NMR experiments at t_{sat} = 2s in the presence of 50 mM* ***Man-d₇ (a)*** *or 50 mM* ***Man-d₇*** *and 4 mM EDTA-d₁₂* ***(b)*** *show major involvement of the biphenyl substituent in the interaction with DC-SIGN ECD, as observed for samples containing only EDTA-d₁₂. The STD spectra were magnified 4-fold.*

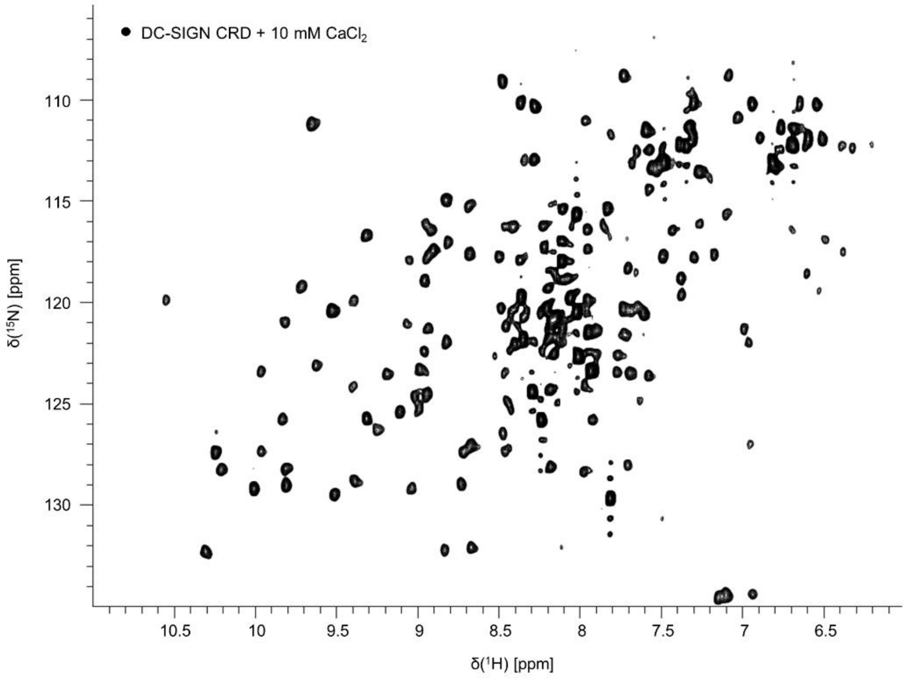

Figure S20. ***^{15}N HSQC NMR spectrum of DC-SIGN CRD in the presence of 10 mM $CaCl_2$ in MES buffer at pH 6.***

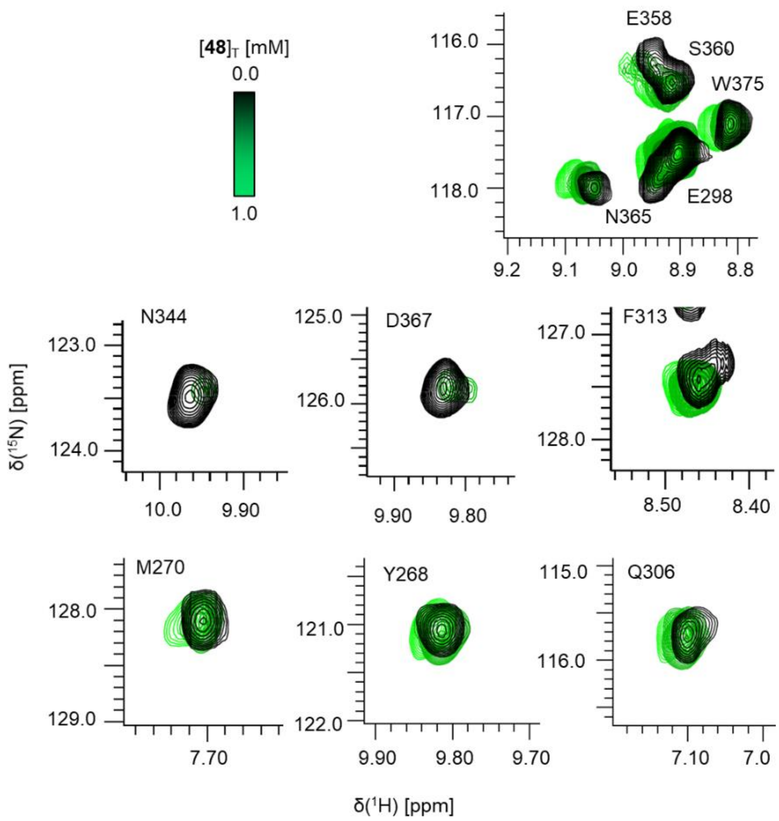

Figure S21: CSPs in ^{15}N HSQC titration of 48 with DC-SIGN CRD (compare with Figure 4 a-c). *CSPs of residues located in the CBS of DC-SIGN CRD (E358, S360, N365, N344, N367, F313) and a distal secondary site (M270, Y268, Q306) upon titration in the presence of Ca^{2+} indicate a dual binding mode of* ***48****.*

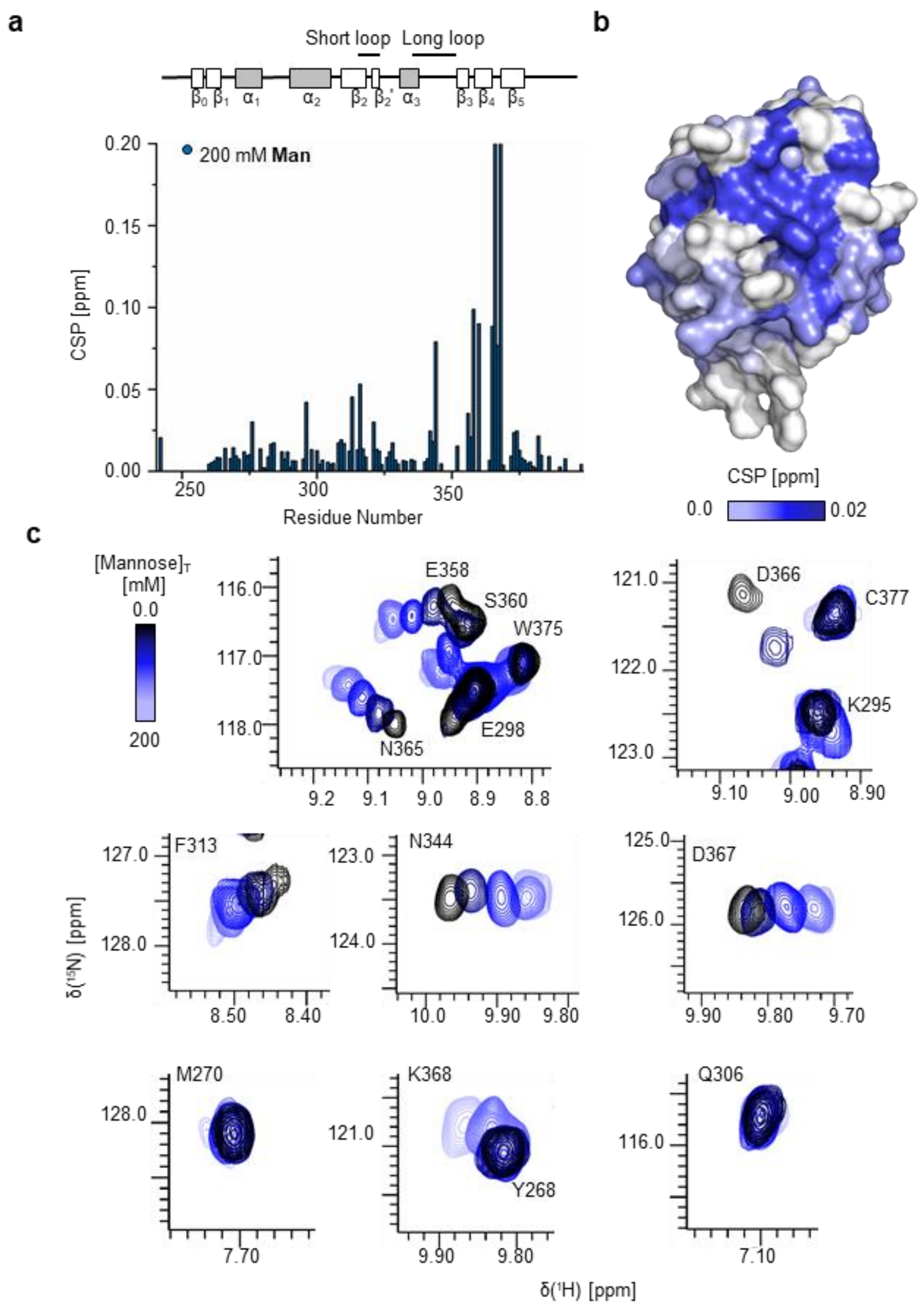

Figure S22: ^{1}H-^{15}N HSQC CSP fingerprint of Man-DC-SIGN CRD interaction. (a) *CSPs were derived from ^{15}N HSQC titration experiments with* ***Man*** *and DC-SIGN CRD.* ***(b)*** *CSPs mapped on a crystal structure of DC-SIGN (PDB code: 1SL4).* ***(c)*** *Shifting resonances observed for the* ***Man*** *titration in the CBS (E358, S360, N365, N366, N344, N367, F313) overlap with those observed in the* ***48*** *titrations (compare with Figure S21).*

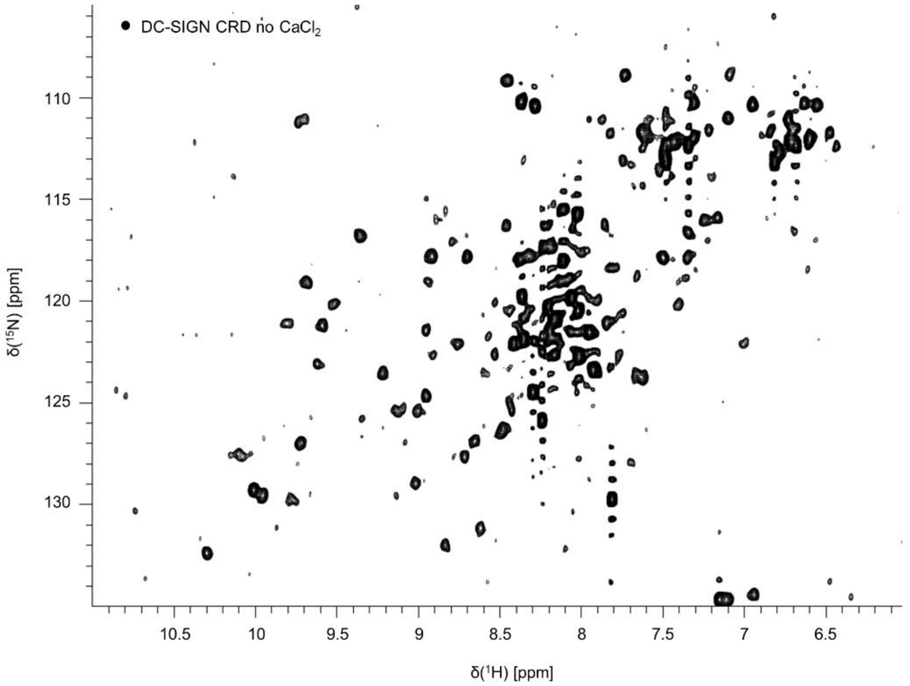

Figure S23. ***^{15}N HSQC NMR spectrum of DC-SIGN CRD in the absence of $CaCl_2$ in MES buffer at pH 6.***

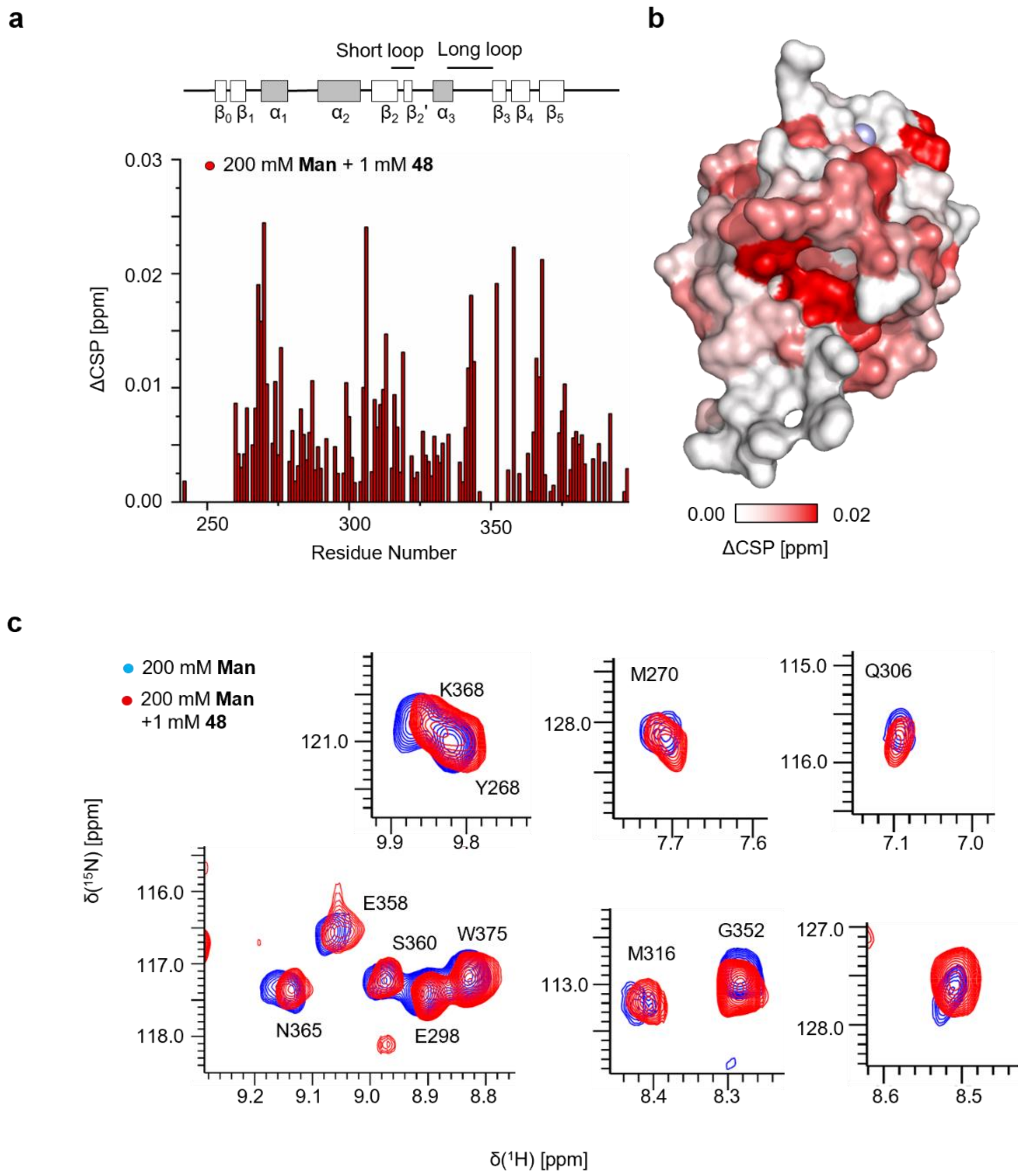

***Figure S24: ^{1}H-^{15}N HSQC CSP fingerprint of 48-DC-SIGN CRD interaction under Man competition. (a)** and **(b)** Similar to measurements in the absence of Ca^{2+}, the ΔCSP map displayed CSP increase for residues in the secondary binding pocket under CBS competition with 200 mM **Man** as well as in regions outside of the secondary binding pocket. **(c)** Examples of residues in the secondary binding pocket (Y268, M270, Q306) and outside of the secondary binding pocket (E358, N365, M316, G352, F313) showing increased CSPs under competitive conditions.*

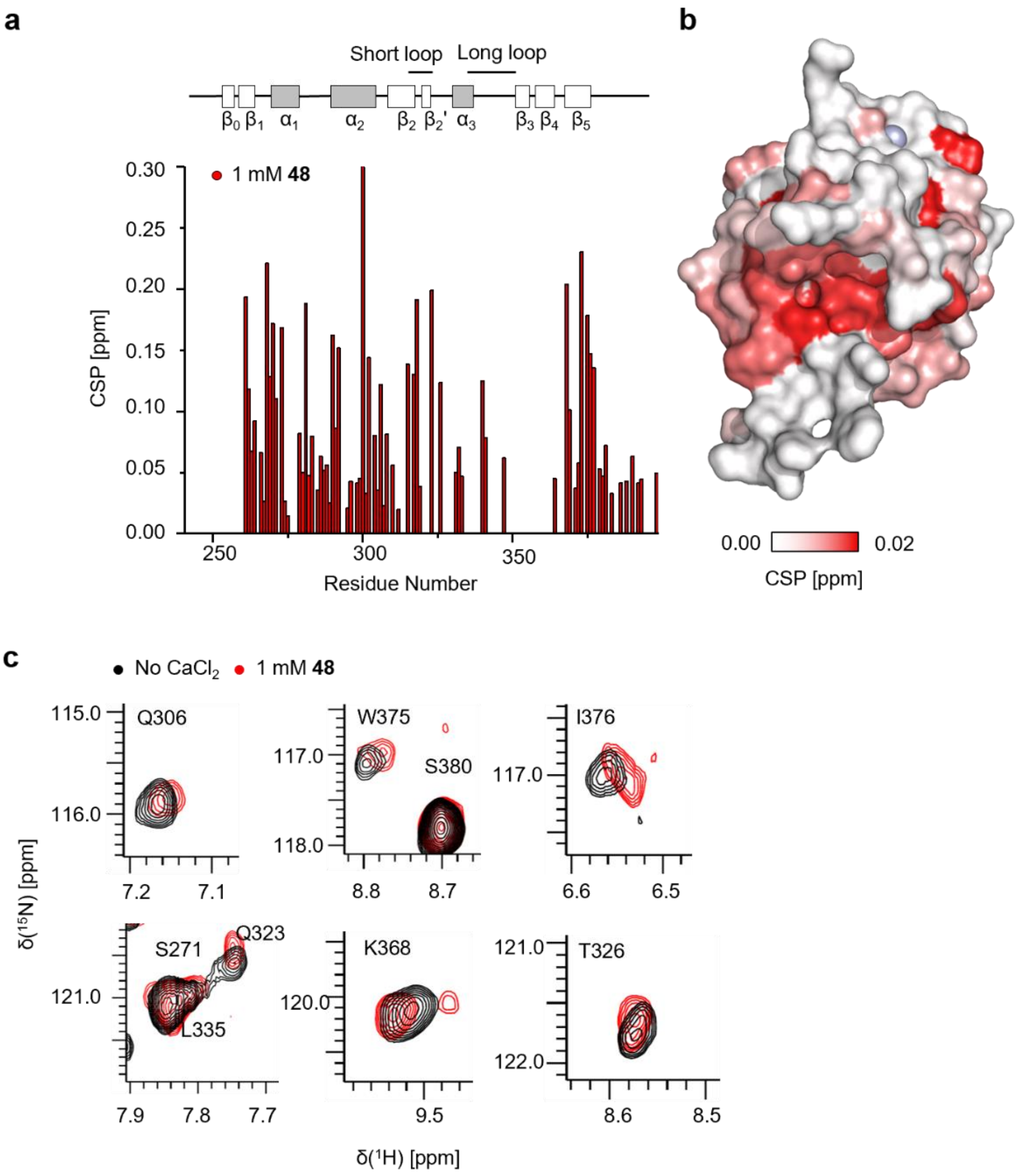

Figure S25: CSPs in ^{1}***H-***^{15}***N HSQC titration of 48 with DC-SIGN CRD in the absence of Ca***$^{2+}$ ***(compare with Figure 4 d-f). (a)*** *CSPs observed in* ^{15}*N HSQC NMR experiments in the absence of Ca*$^{2+}$ *confirm binding of* ***48*** *to the secondary binding pocket under CBS-inhibitory.* ***(b)*** *Mapping of CSPs on X-ray structure of DC-SIGN (PDB code: 1SL4) corroborates interaction with the secondary binding pocket.* ***(c)*** *Examples of residues in the secondary binding pocket (Q306, W375, I376) and outside of the secondary binding pocket (K368, Q323, T326) showing increased CSPs in the absence of Ca*$^{2+}$*.*

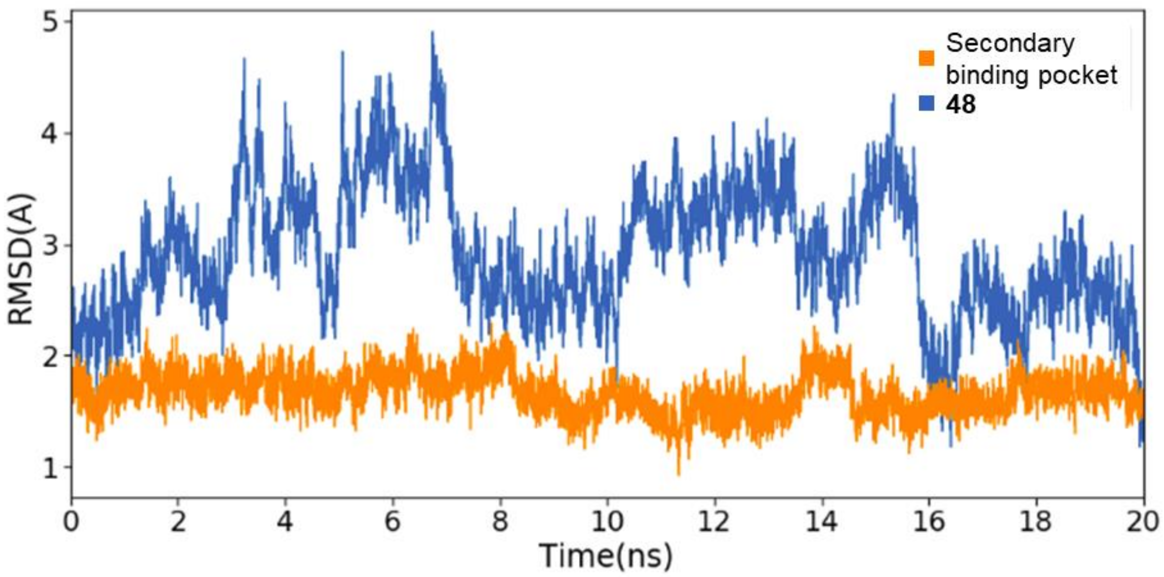

Figure S26: Stability of 48 at the secondary binding pocket of DC-SIGN. *RMSD values are shown as blue and orange lines for mannoside **48** and residues of the secondary binding pocket (T261, F262, Y268, F269, M270, Q306), respectively.*

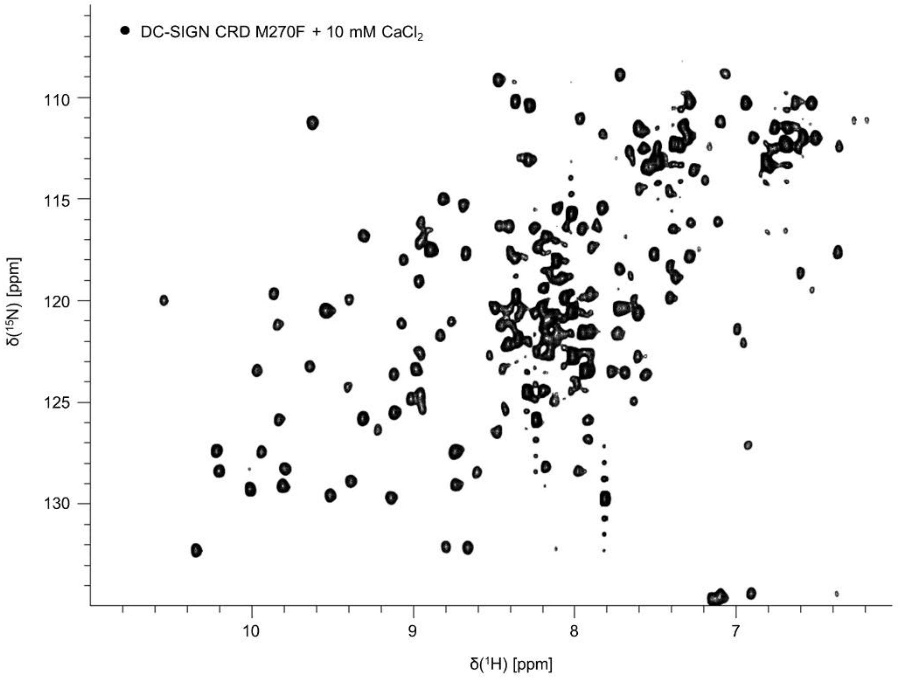

Figure S27. ¹⁵N HSQC NMR spectrum of DC-SIGN CRD M270F in the presence of 10 mM CaCl₂ in MES buffer at pH 6.

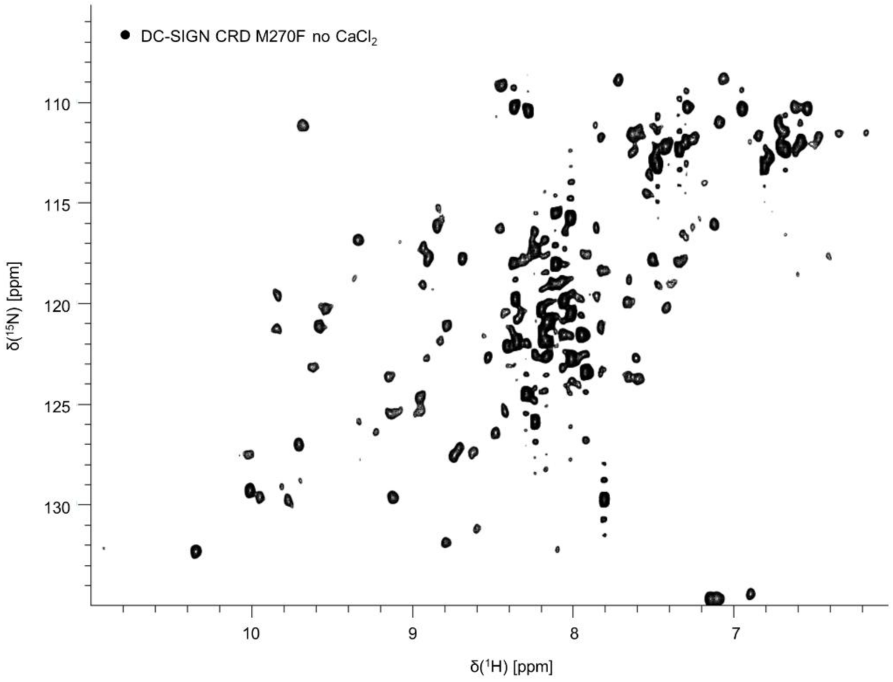

***Figure S28.** ^{15}N HSQC NMR spectrum of DC-SIGN CRD M270F in the absence of Ca^{2+} in MES buffer at pH 6.*

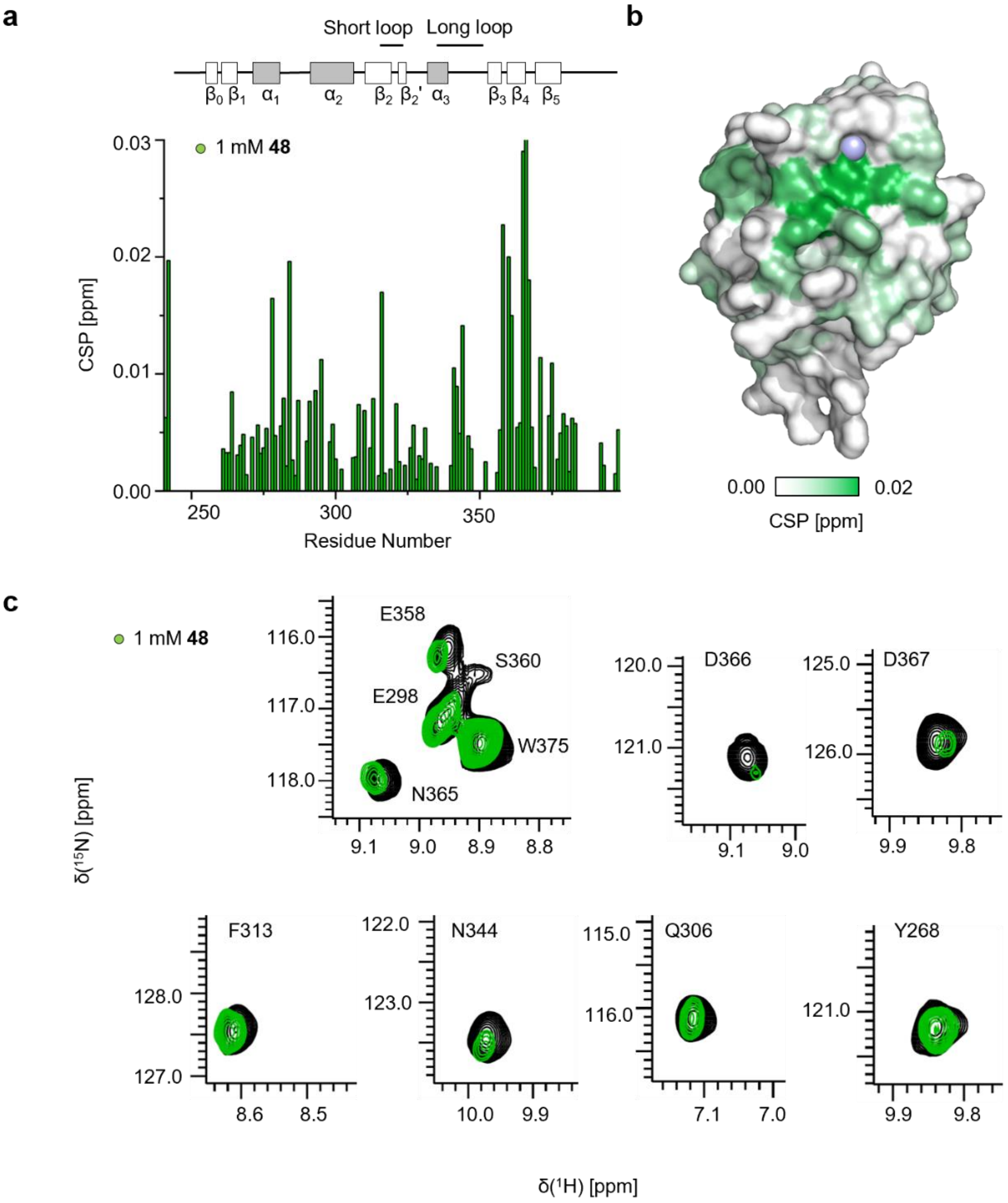

Figure S29: ^{1}H-^{15}N HSQC CSP fingerprint of 48-DC-SIGN CRD M270F mutant. (a) *CSPs observed in ^{15}N HSQC NMR confirm involvement of residues in the CBS in* ***48****-binding to the M270F mutant in the presence of Ca^{2+}.* ***(b)*** *Examples of residues of the CBS showing fast exchanging resonances as well as reduced intensity upon titration (N365, D366).* ***(c)*** *Mapping of CSPs on the X-ray structure of DC-SIGN (PDB code: 1SL4) validates interaction with the CBS.*

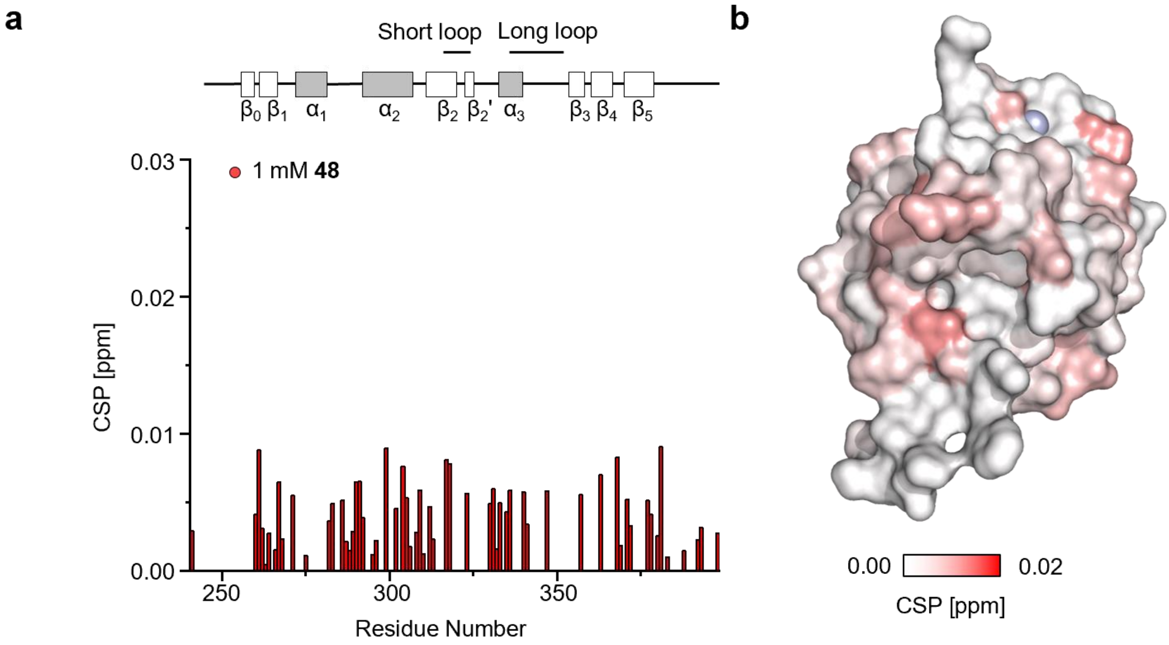

Figure S30: ^{1}H-^{15}N HSQC CSP fingerprint of 48-DC-SIGN CRD M270F mutant in the absence of Ca^{2+}. (a) *^{15}N HSQC NMR experiments with the M270F mutant and **48**. The CSP map demonstrate abrogation of **48**-binding in the absence of Ca^{2+}. Compared with wild-type DC-SIGN, CSPs for the secondary binding pocket are not increased under CBS- inhibitory conditions (Figure 4d, Figure S25). **(b)** Mapping of CSPs on the X-ray structure of DC-SIGN (PDB code: 1SL4).*

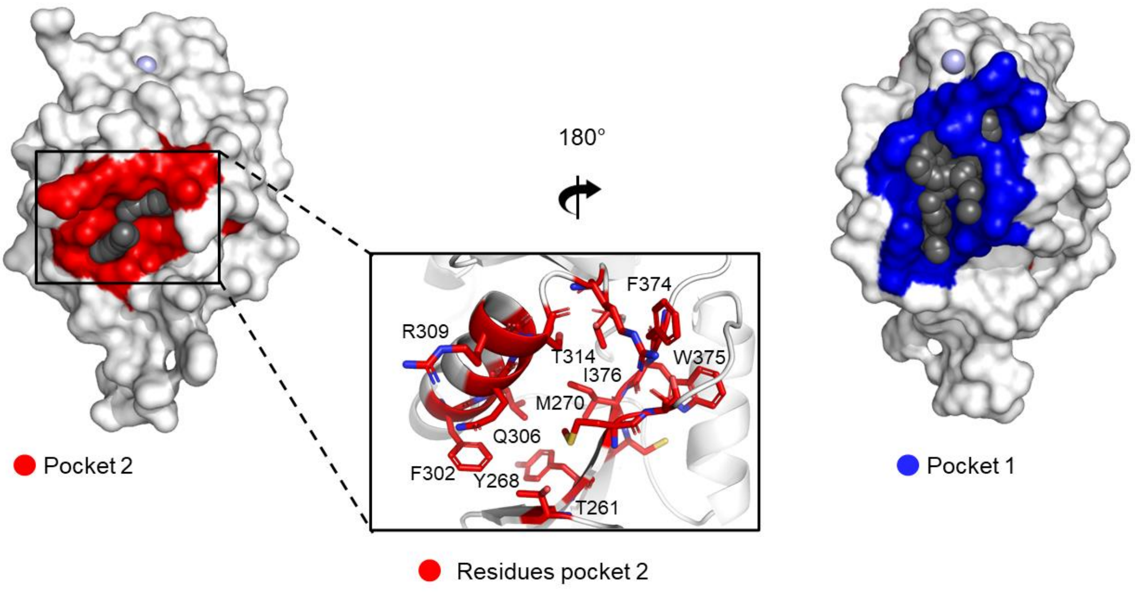

Figure S31: Potential allosteric sites predicted by Allosite. *Two potentially allosteric sites were predicted by the Allosite webserver and mapped onto the DC-SIGN CRD X-ray structure (PDB code: 1SL4).*[10,11] *Allosite pocket 1 (red) corresponds to the binding pocket described in Figure 4 d-f and Figure 5 e. Allosite pocket 2 is shown in blue. The pseudo ligands are shown in grey. Detailed information about both pockets is shown in* ***Table S10****.*

Supporting Tables

Table S1: ^{19}F R2-filtered NMR screening of the mannoside library against Langerin – 1 to 24. *The synthesis of the library was reported previously.*[2,12–15]

Name	Structure	R	$[I]_T$ [mM]	$\Delta R_{2,obs}$ [Hz]	$K_{I,est}$ [mM][a]
**1**	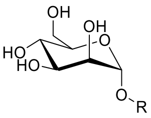	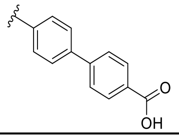	4	3.0	9
**2**		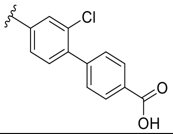	0.5	1.0	8
**3**		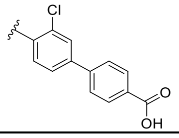	4	3.4	0.3
**4**		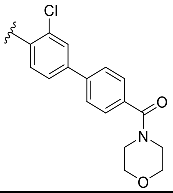	0.5	0.3	6
**5**		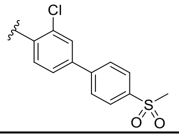	0.5	0.8	2
**6**		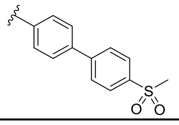	1	0.7	4
**7**		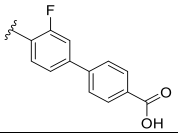	0.5	0.5	3
**8**		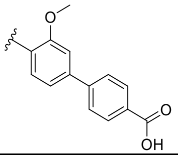	1	0.7	7

**9**		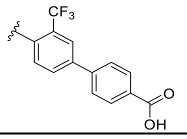	1	3.3	0.2
**10**		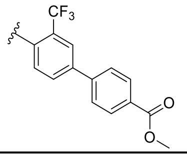	0.5	0.9	2
**11**		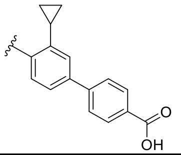	1	1.7	1
**12**		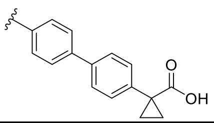	1	1.0	3
**13**		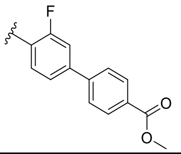	0.1	No competition	
**14**		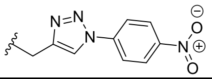	1	0.7	5
**15**		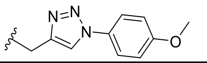	1	1.2	3
**16**		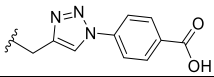	0.5	1	2
**17**		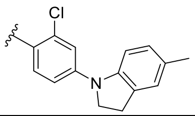	1	3.2	0.4
**18**		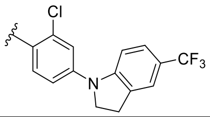	1	2.6	5
**19**		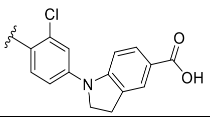	4	3.0	0.7
**20**		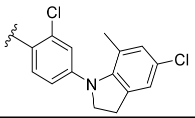	1	3.5	0.3

**21**		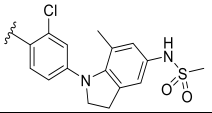	1	2.0	1
**22**		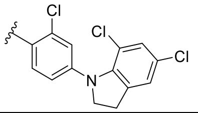	1	2.9	0.3
**23**		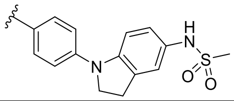	1	1.8	1
**24**		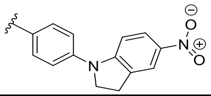	0.5	0.9	1

[a]Estimated K_I values were determined *via* ^{19}F R_2-filtered NMR experiments at a single competitor concentration.

Table S2: ^{19}F R2-filtered NMR screening of the mannoside library against Langerin – 25 to 27. *The synthesis of the library was reported previously.*[16,17]

**Name**	**Structure**	**R**	**$[I]_T$ [mM]**	**$\Delta R_{2,obs}$ [Hz]**	**$K_{I,est}$ [mM][a]**
**25**	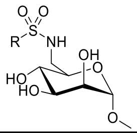	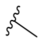	1	1.3	3
**26**		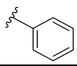	1	1.8	2
**27**		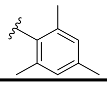	1	1.7	2

[a]Estimated K_I values were determined *via* ^{19}F R_2-filtered NMR experiments at a single competitor concentration.

Table S3: Affinity validation for mannosides and Langerin.

Name	Structure	K_I [mM]	K_D [mM]	Relative potency[a]
**46**	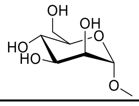	13±3	7±2	1.0
**3**	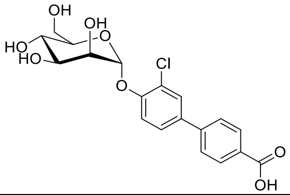	0.39±0.05		33
**9**	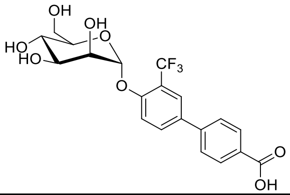	0.23±0.03	0.5±0.2	57
**20**	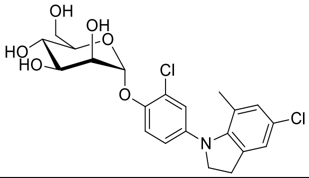	0.33±0.02		39
**25**	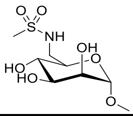	3.0±0.2	2.9±0.4	4.3

[a]The relative potency was calculated utilizing the K_I value determined for **46**.

Table S4: Structure-activity relationship for introduction of a linker to 9 for Langerin

Name	Structure	K_I [mM]	K_D [mM]	Relative potency[a]
**Man**	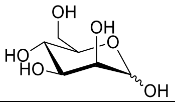	4.5±0.5[b]	5.8±0.3[b]	2.2
**45**[b]	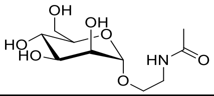	10±1[b]	12±1[b]	1.0
**9**	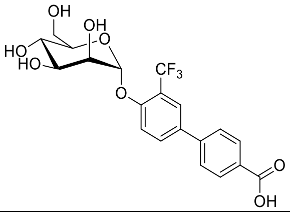	0.23±0.03	0.5±0.02	44
**43**	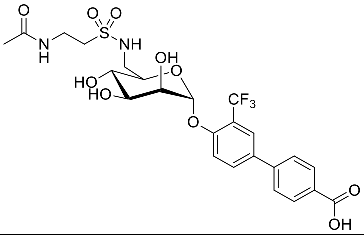	0.25±0.07	0.46±0.09	40

[a] The relative potency was calculated utilizing the K_I value determined for **45**.
[b] These values were previously published and **45** was prepared as previously reported.[6]

Table S5: Parameters for 19F R2-filtered NMR assay with DC-SIGN ECD.

Name	Structure	Parameter	DC-SIGN ECD
**48**	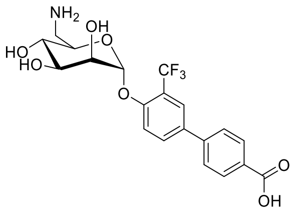	$R_{2,f}[s^{-1}]$	9.5 ± 0.1
		$R_{2,b}[s^{-1}]$	540 ± 55
		K_D[mM]	0.48 ± 0.1
		$K_{I,Man}$[mM] [a]	-
**49**[b]	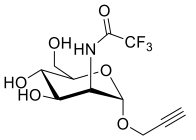	$R_{2,f}[s^{-1}]$	2.9 ± 0.3
		$R_{2,b}[s^{-1}]$	541 ± 55
		K_D[mM]	2.1 ± 0.2
		$K_{I,Man}$[mM][a]	2.4 ± 0.8

[a]Titration showed incomplete inhibition. No K_I could be calculated using $R_{2,f}$ and $R_{2,b}$ determined for **48**.

[b] **49** was prepared as previously reported.[6]

Table S6: *Comparison of affinity values for **48** determined from ^{19}F NMR and ^{15}N HSQC NMR assays*

Assay	Parameter	Value
^{19}F R_2-filtered NMR RDA	K_I [mM]	1.15 ± 0.01
^{19}F R_2-filtered NMR	K_D[mM]	0.48 ± 0.06
^{19}F NMR (^{19}F CSPs)	K_D[mM]	0.37 ± 0.06
^{15}N HSQC NMR	K_D[mM]	0.46 ± 0.16

Table S7: Coupling efficiency of prepared ligand-functionalized lipids

Glycolipid	Coupling efficiency
**42-Lip**	55%
**48-Lip**	83%
**Man-Lip**	82%
**Fuc-Lip**	94%
**LeX-Lip**	41%
**50-Lip**	87%

Table S8: DLS characterization of liposomal formulations used in this study.

Liposome	Z average size [nm]	PDI	Zeta potential [mV]
naked	158	0.075	-28.1
**Man-Lip** 4.75%	169	0.089	-27.3
**Man-Lip** 4.25%	151	0.038	-26.4
**Man-Lip** 3.75%	148	0.058	-25.7
**Man-Lip** 3.25%	155	0.115	-27.7
**Man-Lip** 2.75%	150	0.076	-31.8
**Man-Lip** 4.25%<br>**42-Lip** 0.32%	155	0.129	-30.9
**Man-Lip** 3.75%<br>**42-Lip** 0.63%	157	0.069	-28.9
**Man-Lip** 3.25%<br>**42-Lip** 0.95%	151	0.170	-32.1

**Man-Lip** 2.75%<br>**42-Lip** 1.26%	154	0.090	-31.2
**50-Lip** 4.75%	155	0.113	-22.4
**50-Lip** 4.25%<br>**42-Lip** 0.34%	154	0.108	-21.5
**50-Lip** 3.75%<br>**42-Lip** 0.68%	153	0.093	-22.3
**50-Lip** 3.25%<br>**42-Lip** 1.02%	163	0.159	-23.6
**50-Lip** 2.75%<br>**42-Lip** 1.36%	151	0.111	-24.4
**48-Lip** 0.42%	147	0.139	-25.5
**48-Lip** 0.83%	166	0.116	-27.9
**48-Lip** 1.25%	151	0.159	-30.4
**48-Lip** 1.66%	222	0.232	-31.6
**Man-Lip** 4.25%<br>**48-Lip** 0.42%	225	0.018	-26.4
**Man-Lip** 3.75%<br>**48-Lip** 0.83%	152	0.195	-24.2
**Man-Lip** 3.25%<br>**48-Lip** 0.15	162	0.226	-27.4
**Man-Lip** 2.75%<br>**48-Lip** 1.66	234	0.222	-22.6
**Fuc-Lip** 2.41%	134	0.179	-29.4

**LeX-Lip** 1.05%	130	0.179	-26.0
**Man-Lip** 2.0%<br>**48-Lip** 1.02%	129	0.176	-28.1
**LeX-Lip** 1.02%<br>**42-Lip** 1.37%	140	0.184	-32.3
**Man-Lip** 2.0%<br>**Fuc-Lip** 2.34%	138	0.094	-25.3
**Fuc-Lip** 2.34%<br>**LeX-Lip** 1.02%	142	0.088	-24.2
**Fuc-Lip** 2.34%<br>**42-Lip** 1.37%	133	0.202	-29.4

Table S9: Energy values for amino acid substitution at position 270 of DC-SIGN. *The values represent the difference of free energy after substitution. The values were obtained using FoldX.*[18]

**Mutation**	**ΔΔG**	**Mutation**	**ΔΔG**
MA270F	-1.10884	MA270N	1.56714
MA270Y	-0.207919	MA270C	1.67328
MA270I	0.197898	MA270T	1.75469
MA270L	0.329824	MA270E	1.94657
MA270P	0.715913	MA270Q	2.02411
MA270W	0.951441	MA270A	2.10555
MA270H	1.21562	MA270D	2.30746
MA270R	1.22301	MA270S	2.51665
MA270V	1.29116	MA270G	2.54774
MA270K	1.47935		

Table S10: Details for allosteric pockets predicted by Allosite.[10,11]

	Residues	Volume	SASA*	Drug-gability Score*	logitProb*	nmaScore*	hitScore*
**Allosite pocket 1**	W277, H278, I281, K285, A289, Q290, L291, S319, D320, L321, N322, Q232, T326, Q328, W329, V330, D331, G332	595.576	207.562	0.537	0.556	0.605	0.566
**Allosite pocket 2**	T261, Y268, M270, S271, F302, L303, Q306, S307, R309, S310, R312, T314, F374, W375, I376	776.724	391.161	0.802	0.709	0.302	0.627

Supporting Schemes

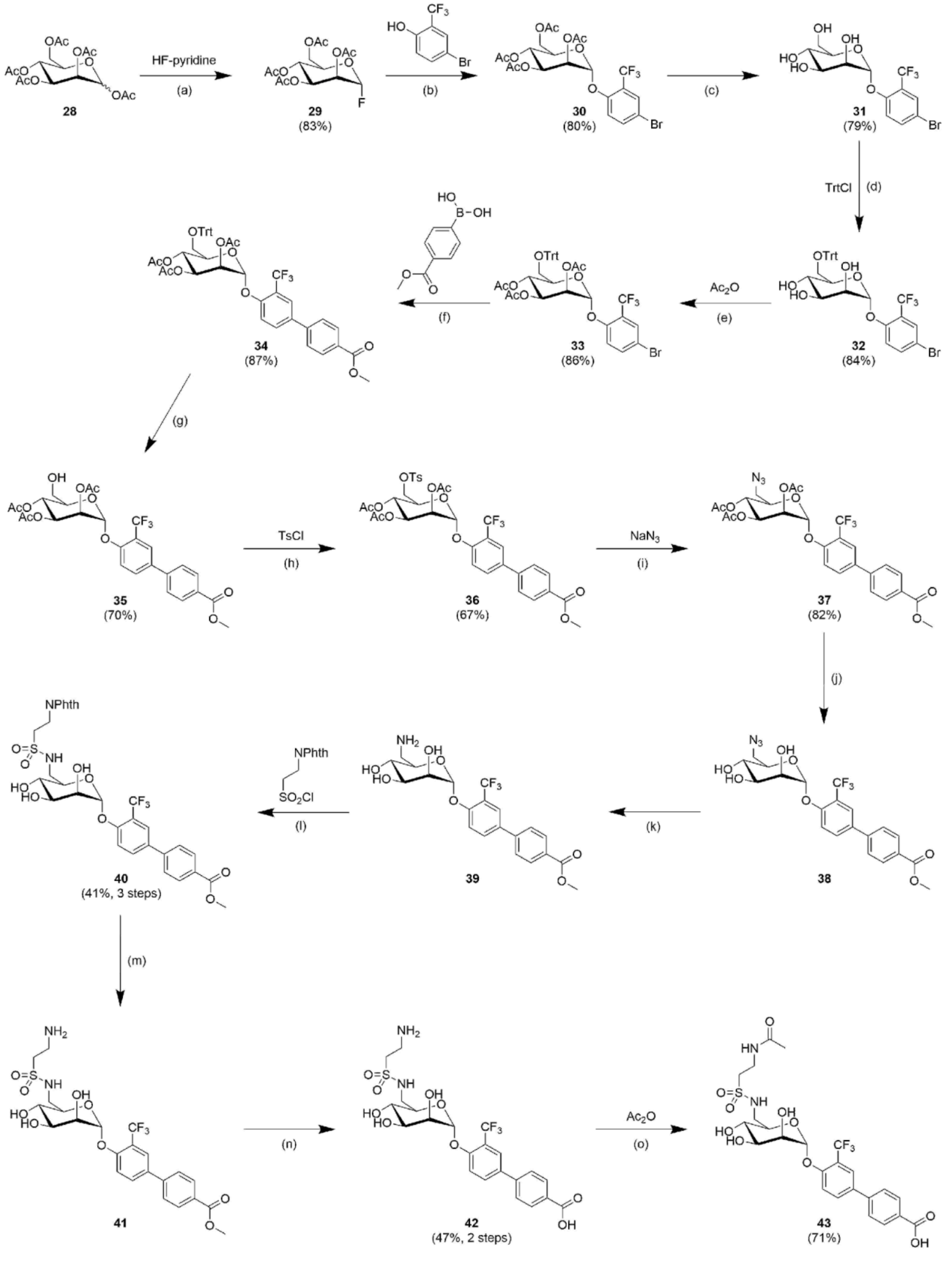

Scheme S1: ***Synthesis of mannosides 42 and 43.*** *Intermediate **30** was prepared as previously published.*[14,19] *Reaction conditions for the preparation of **43**: (a) DCM, 0°C to 40°C; (b) $BF_3{\cdot}OEt_2$, anhydrous DCM, 0°C to room temperature; (c) MeONa, MeOH, room temperature; (d) DMAP, pyridine, room temperature; (e) pyridine, room temperature; (f) Pd(dppf)$Cl_2{\cdot}CH_2Cl_2$, K_3PO_4, anhydrous DMF, 80°C; (g) FeCl3, H_2O, DCM, room temperature; (h) DMAP, anhydrous pyridine, 0°C to room temperature; (i) 1,4,7,10,13,16-hexaoxacyclooctadecane, anhydrous DMF, 0°C to 80°C; (j) MeONa, anhydrous MeOH, room temperature; (k) H_2, Pd/C, 1,4-dioxane, room temperature; (l) Et_3N, anhydrous DMF, 0°C to room temperature; (m) hydrazine monohydrate, MeOH, room temperature; (n) 2 M aqueous NaOH, MeOH, room temperature; (o) MeOH, room temperature.*

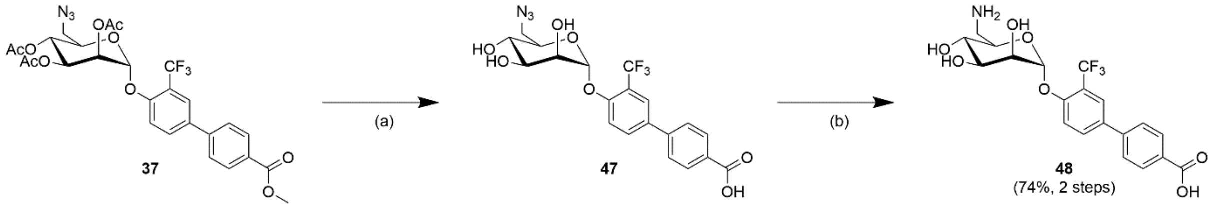

Scheme S2: Synthesis of mannoside 48. *Reaction conditions for the preparation of **48**: (a) MeONa, MeOH with H_2O traces, room temperature; (b) H_2, Pd/C, 1,4-dioxane, room temperature.*

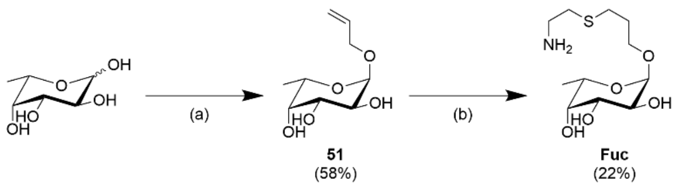

Scheme S3: Synthesis of Fuc for conjugation to PEGylated lipids. *Reaction conditions for the preparation of* ***Fuc****: (a) Amberlite IR120 (H^+), allyl alcohol, room termperature; (b) cysteamine hydrochloride, H_2O, 50°C.* ***51*** was prepared as previously reported.[20]

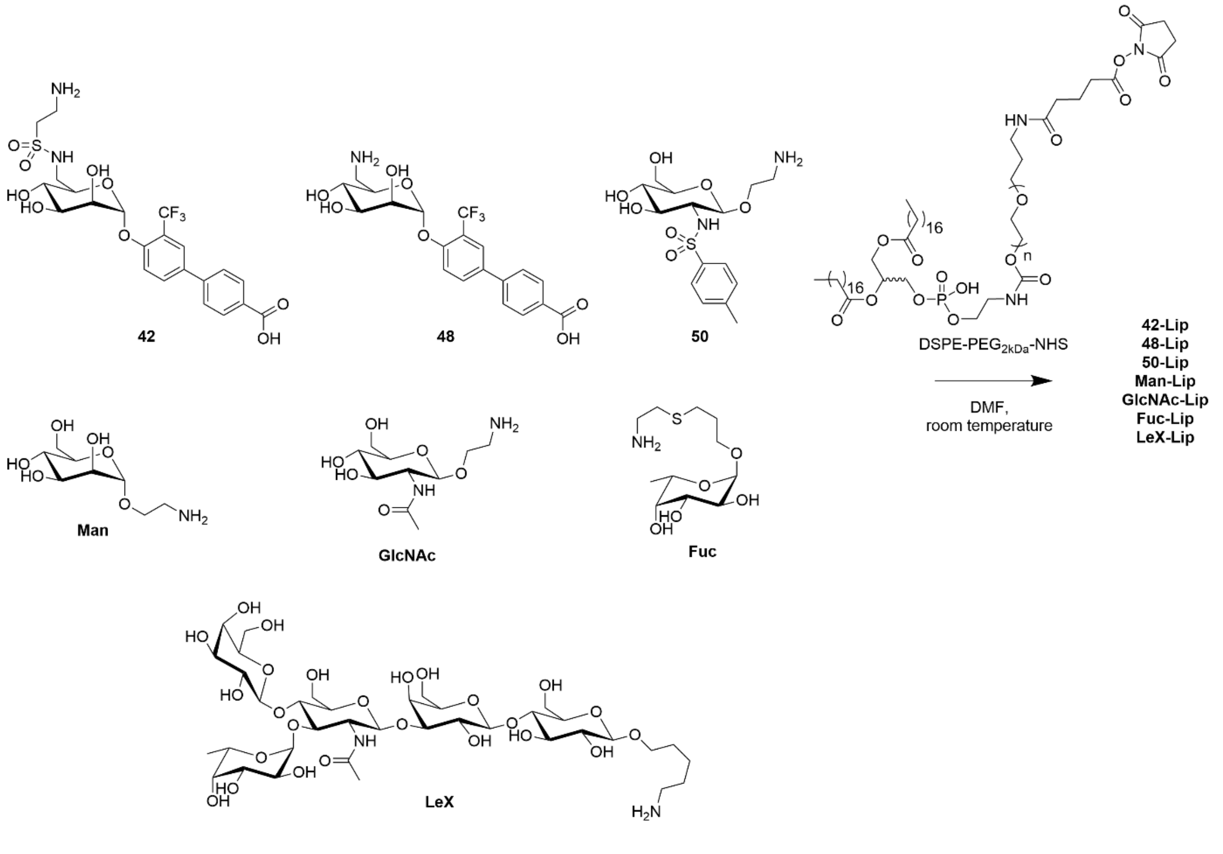

Scheme S4: Chemical Structures of ligands and conjugation to PEGylated lipids. *Glycolipids were purified by dialysis and coupling efficiency was quantified by ^1H NMR (**Table S7**). **50**, **Man**, **GlcNAc** and **LeX** were synthesized as previously described.*[4,21–23]

Methods

Synthetic chemistry - procedures

General remarks. Reagents and solvents used were purchased from Sigma Aldrich unless indicated otherwise and used as supplied without any further purification. MPLC and reversed-phase MPLC was conducted on a Combiflash R_f 200 (Teledyne Isco) using RediSep and RediSep C_{18} columns (Teledyne Isco). Analytical thin layer chromatography (TLC) was performed on glass plates coated with silica gel at a pore size of 60 Å (Machery Nagel or Merck). Compounds were detected *via* 3-methoxyphenol reagent (0.2% 3-methoxyphenol in EtOH: 2 N sulfuric acid in EtOH (1:1)), ninhydrin reagent (1.5 g ninhydrin in 15 mL AcOH and 500 mL MeOH) and CAM reagent (1.0 g $Ce(SO_4)_2{\cdot}4H_2O$ and 2.5 g ammonium molybdate pentahydrate in 96 mL of H_2O and 6 mL of concentrated H_2SO_4) upon heating or *via* UV adsorption (λ = 254 nm). NMR experiments were conducted on an Avance III 400 MHz spectrometer (Bruker), an Avance DMX 500 MHz spectrometer (Bruker) or an Ascend 700 MHz spectrometer (Bruker). Chemical shifts were referenced to the internal standards DMSO ($\delta(^{1}H)$ = 3.33 ppm), $CHCl_3$ ($\delta(^{1}H)$ = 7.26 ppm and $\delta(^{13}C)$ = 77.1 ppm), H_2O ($\delta(^{1}H)$ = 7.26 ppm), MeOH ($\delta(^{1}H)$ = 3.31 ppm, $\delta(^{13}C)$ = 49.0 ppm), TMS ($\delta(^{1}H)$ = 0.00 ppm, $\delta(^{13}C)$ = 0.0 ppm) and TFA ($\delta(^{19}F)$ = 76.55 ppm). Coupling constants are reported in Hz and coupling patterns are indicated as s for singlet, d for doublets, dd for doublets of doublets, ddd for doublets of doublets of doublets, t for triplets, dt for doublets of triplets, td for triplet of doublets, q for quartets and m for multiplets. Resonances were assigned by means of COSY, TOCSY, ^{13}C HSQC and ^{13}C H2BC NMR experiments.[24] NMR spectra were processed in MestReNova (MestreLab Research). The specific optical rotation was determined using a Model 341 polarimeter (PerkinElmer). ESI-MS analysis was conducted using an 1100 Series LC/MS coupled to a Micromass ZQ spectrometer (Waters) or directly using an amaZon SL spectrometer (Bruker). HR ESI-MS analysis was conducted using a 6210 ESI-TOF spectrometer (Agilent). ATR-FTIR spectra were acquired using a Spectrum 100 FTIR spectrometer (PerkinElmer). Reversed-phase preparative HPLC was performed on a 1100 Series LC/MS (Thermo Scientific) using a preparative Nuleodur C18 column (Machery Nagel). Analytical HPLC was performed on an Aqcuity UPLC system using an analytical BEH C18 column (Waters) or on a 1200 Series LC/MS coupled to a 6130 ESI-Q spectrometer using an analytical Atlantis T3 column (Agilent).

4'-Bromo-2'-(trifluoromethyl)phenyl α-D-mannopyranoside

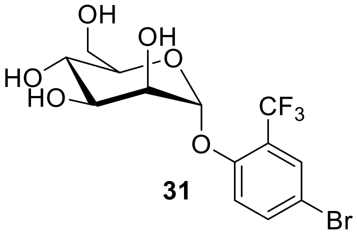

31

Freshly prepared 1 M NaOMe in MeOH (2 mL) was added to a solution of **30** (4.41 g, 7.25 mmol) in MeOH (50 mL). The reaction mixture was stirred overnight at room temperature under argon atmosphere. After neutralization with acetic acid, solvents were removed *in vacuo*, and the residue was purified *via* MPLC (gradient: 100% DCM to 100% MeOH in 20 min) to yield **31** as a white solid (2.55 g, 5.75 mmol, 79%).

^{1}H NMR (500.0 MHz, MeOD): δ = 7.77 – 7.69 ppm, m, 2 H (aromatic H of phenyl); δ = 7.47 ppm, d, 1 H, J = 8.7 Hz (aromatic H of phenyl); δ = 5.61 ppm, d, 1 H, J = 1.9 Hz (H1); δ = 4.04 ppm, dd, 1 H, J = 3.4, 1.9 Hz (H2), δ = 3.91 ppm, dd, 1 H, J = 9.5, 3.4 Hz (H3); δ = 3.81 ppm, dd, 1 H, J = 12.1, 2.4 Hz (H6b); δ = 3.78 – 3.68 ppm, m, 2H (H4, H6a); δ = 3.55 ppm, ddd, 1 H, J = 9.8, 5.9, 2.4 Hz (H5).

^{13}C NMR (125.8 MHz, MeOD): δ = 154.7 ppm, 1 C (aromatic C of phenyl); δ = 137.7 ppm, 1 C (aromatic C of phenyl); δ = 130.6 ppm, q, 1 C, J = 5.4 Hz (aromatic C of phenyl); δ = 124.2 ppm, q, 1 C, J = 272.3 Hz (*C*F$_3$); δ = 122.1 ppm, q, 1 C, J = 32.0 Hz (aromatic C of phenyl), δ = 119.3 ppm, 1 C (aromatic C of phenyl); δ = 114.6 ppm, 1 C (aromatic C of phenyl); δ = 100.4 ppm, 1 C (C1); δ = 76.2 ppm, 1 C (C5); δ = 72.2 ppm, 1 C (C3); δ = 71.6 ppm, 1 C (C2); δ = 68.1 ppm, 1 C (C4); δ = 62.7 ppm, 1 C (C6).

ESI-MS for $C_{13}H_{14}BrF_3O_6$: $m\cdot z^{-1}(M+Na^+)_{calc}$ = 424.98; $m\cdot z^{-1}(M+Na^+)_{obs}$ = 424.95.

4'-Bromo-2'-(trifluoromethyl)phenyl 6-O-trityl-α-D-mannopyranoside

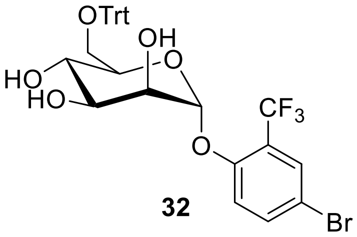

32

31 (800 mg, 1.98 mmol), trityl chloride (663 mg, 2.38 mmol) and a catalytic amount of DMAP were dissolved in pyridine (20 mL). The reaction mixture was stirred overnight at room temperature under argon atmosphere. The solvent was removed *in vacuo* by co-evaporation with toluene and the residue was purified *via* MPLC (gradient: 100% PE to 100% EtOAc in 20 min) to yield **32** as a white solid (1.07 g, 1.66 mmol, 84%).

^1H NMR (500.0 MHz, $CDCl_3$): δ = 7.55 ppm, dd, 1 H, J = 8.9, 2.5 Hz (aromatic H of phenyl or OTrt); δ = 7.41 – 7.34 ppm, m, 6 H (aromatic H of phenyl or OTrt); δ = 7.32 – 7.18 ppm, m, 11 H (aromatic H of phenyl or OTrt); δ = 5.59 ppm, d, 1 H, J = 1.6 Hz (H1); δ = 4.17 ppm, dd, 1 H, J = 3.5, 1.7 Hz (H2); δ = 3.96 ppm, dd, 1 H, J = 9.3, 3.5 Hz (H3); δ = 3.79 ppm, t, 1 H, J = 9.5 Hz (H4); δ = 3.62 ppm, ddd, 1 H, J = 9.9, 5.7, 4.4 Hz (H5); δ = 3.45 ppm, dd, 1 H, J = 10.2, 4.4 Hz (H6a); δ = 3.37 ppm, dd, 1 H, J = 10.2, 5.8 Hz (H6b); δ = 2.91 – 2.60 ppm, m, 3 H (O*H*).

^{13}C NMR (125.8 MHz, $CDCl_3$): δ = 153.0 ppm, 1 C (aromatic C of phenyl or OTrt); δ = 149.9 ppm, 1 C (aromatic C of phenyl or OTrt); δ = 143.7 ppm, 1 C (aromatic C of phenyl or OTrt); δ = 136.3 ppm, 1 C (aromatic C of phenyl or OTrt); δ = 136.2 ppm, 1 C (aromatic C of phenyl or OTrt); δ = 130.1 ppm, q, 1 C, J = 5.5 Hz (aromatic C of phenyl); δ = 128.7 ppm, 1 C (aromatic C of phenyl or OTrt); δ = 128.1 ppm, 1 C (aromatic C of phenyl or OTrt); δ = 127.4 ppm, 1 C (aromatic C of phenyl or Trt); δ = 121.4 ppm, q, 1 C, J = 32.0 Hz (aromatic C of phenyl); δ = 117.8 ppm, 1 C (aromatic C of phenyl or OTrt); δ = 114.2 ppm, 1 C (aromatic C of phenyl); δ = 98.0 ppm, 1 C (C1); δ = 71.6 ppm, 1 C (C5); δ = 71.4 ppm, 1 C (C3); δ = 70.0 ppm, 1 C (C2); δ = 69.6 ppm, 1 C (C4); δ = 64.4 ppm, 1 C (C6).

ESI-MS for $C_{32}H_{28}BrF_3O_6$: $m \cdot z^{-1}(M+Na^+)_{calc}$ = 667.09; $m \cdot z^{-1}(M+Na^+)_{obs}$ = 667.17.

4’-Bromo-2’-(trifluoromethyl)phenyl 2,3,4-tri-O-acetyl-6-O-trityl-α-D-mannopyranoside

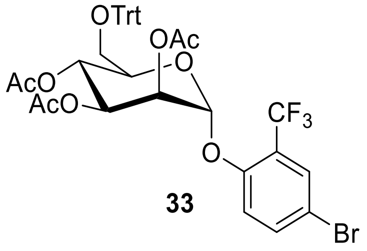

33

32 (1.07 g, 1.66 mmol) was dissolved in pyridine (20 mL) and acetic anhydride was added under argon atmosphere and the reaction mixture was stirred overnight at room temperature. Solvents were removed *in vacuo* and the residue was co-evaporated with toluene, dissolved in DCM (50 mL), washed with 1 M HCl and sat. $NaHCO_3$. The organic phase was dried with Na_2SO_4 and solvents were evaporated *in vacuo*. The residue was purified *via* MPLC (gradient: 100% PE to 100% EtOAc in 20 min) to yield **33** as a white solid (1.10 g, 1.43 mmol, 86%).

^1H NMR (500.0 MHz, $CDCl_3$): δ = 7.76 ppm, d, 1 H, J = 2.4 Hz (aromatic H of phenyl or OTrt); δ = 7.57 ppm, dd, 1 H, J = 8.9, 2.5 Hz (aromatic H of phenyl or OTrt); δ = 7.37 – 7.33, m, 7 H (aromatic H of phenyl or OTrt); δ = 7.30 – 7.26 ppm, m, 4 H (aromatic H of phenyl or OTrt); δ = 7.26 – 7.21 ppm, m, 5 H (aromatic H of phenyl or OTrt); δ = 5.62 ppm, d, 1 H, J = 1.5 Hz (H1); δ = 5.47 – 5.43 ppm, m, 2 H (H2, H3); δ = 5.29 ppm,

ddd, 1 H, J = 11.5, 9.7, 1.7 Hz (H4); δ = 3.90 ppm, ddd, 1 H, J = 10.2, 6.3, 2.2 Hz (H5); δ = 3.23 ppm, dd, 1 H, J = 10.6, 6.3 Hz (H6a); δ = 3.13 ppm, dd, 1 H, J = 10.6, 2.2 Hz (H6b); δ = 2.20 ppm, s, 3 H (OCOCH_3); δ = 2.00 ppm, s, 3 H (OCOCH_3); δ = 1.75 ppm, s, 3 H (OCOCH_3).

^{13}C NMR (125.8 MHz, $CDCl_3$): δ = 170.1 ppm, 1 C (O*C*OCH_3); δ = 169.8 ppm, 1 C (O*C*OCH_3); δ = 169.6 ppm, 1 C (O*C*OCH_3); δ = 152.5, 1 C (aromatic C of phenyl or OTrt); δ = 143.7 ppm, 1 C (aromatic C of phenyl or OTrt); δ = 136.3 ppm, 1 C (aromatic C of phenyl or OTrt); δ = 130.3 ppm, q, 1 C, J = 5.0 Hz (aromatic C of phenyl); δ = 128.8 ppm, 1 C (aromatic C of phenyl or OTrt); δ = 127.9 ppm, 1 C (aromatic C of phenyl or OTrt); δ = 127.2 ppm, 1 C (aromatic C of phenyl or OTrt); δ =121.9 ppm, q, 1 C, J = 31.9 Hz (aromatic C of phenyl); δ = 118.0 ppm, 1 C (aromatic C of phenyl or OTrt); δ = 114.8 ppm, 1 C (aromatic C of phenyl or OTrt); δ = 95.8 ppm, 1 C (C1); δ = 71.7 ppm (C5); δ = 69.4 and 68.8 ppm, 2 C (C2, C3); δ = 66.1 ppm, 1 C (C4); δ = 62.5 ppm (C6); δ = 21.0 ppm, 1 C (OCO*C*H_3); δ = 20.8 ppm, 1 C (OCO*C*H_3); δ = 20.6 ppm, 1 C (OCO*C*H_3).

$[\alpha]^{20}_{D}$ = +62.2° (c = 1.00, $CHCl_3$).

ESI-MS for $C_{38}H_{34}BrF_3O_9$: $m\cdot z^{-1}(M+Na^+)_{calc}$ = 793.12; $m\cdot z^{-1}(M+Na^+)_{obs}$ = 793.28.

Methyl 4'-(2'',3'',4''-tri-O-acetyl-6''-O-trityl-α-D-mannopyranosyloxy)-3'-trifluoromethylbiphenyl-4-carboxylate

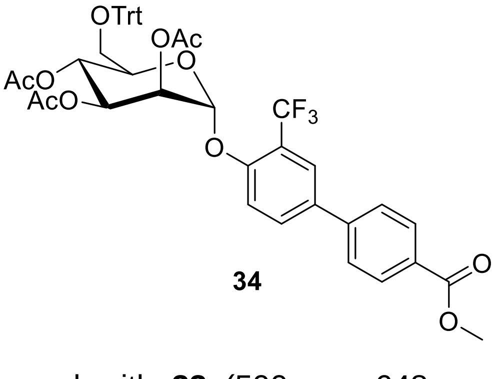

34

A Schlenk tube was charged with **33** (500 mg, 648 µmol), (4-(methoxycarbonyl) phenyl)boronic acid (128 mg, 713 µmol), K_3PO_4 (206 mg, 972 µmol) and Pd(dppf)$Cl_2\cdot CH_2Cl_2$ (16 mg, 19 µmol). The tube was sealed, evacuated and subsequently flushed with argon. This procedure was repeated twice. Next, anhydrous DMF (6 mL) was added under a stream of argon. The reaction mixture was degassed *via* ultrasonication, flushed with argon and stirred overnight at 80°C. Product formation was monitored *via* TLC (2:1 (PE:EtOAc). The reaction mixture was cooled to room temperature and diluted with EtOAc (50 mL). Next, the diluted reaction mixture was extracted with H_2O (50 mL) and brine (50 mL) and dried with Na_2SO_4. Solvents were removed *in vacuo* and the residue was purified *via* MPLC (gradient: 100% PE to 100% EtOAc in 20 min) to yield **34** as a white solid (464 mg, 561 µmol, 87%).

^1H NMR (500.0 MHz, $CDCl_3$): δ = 8.14 – 8.10 ppm, m, 2 H (aromatic H of biphenyl); δ = 7.89 ppm, d, 1 H, J = 2.2 Hz (aromatic H of biphenyl); δ = 7.69 ppm, dd, 1 H, J = 9.0, 2.2 Hz (aromatic H of biphenyl); δ = 7.62 – 7.58 ppm, m, 2 H (aromatic H of biphenyl); δ = 7.55 ppm, d, 1 H, J = 8.9 Hz (aromatic H of biphenyl); δ = 7.39 – 7.33 ppm, m, 6 H, (aromatic H of OTrt); δ = 7.25 – 7.17 ppm, m, 9 H, (aromatic H of OTrt); δ = 5.70 – 5.72 ppm, m, 1 H (H1); δ = 5.52 – 5.47 ppm, m, 2 H (H2, H3); δ = 5.36 – 5.29 ppm, m, 1 H (H4); δ = 3.95 ppm, s, 3 H (OCH_3); δ = 3.92 ppm, ddd, 1 H, J = 10.2, 5.9, 2.0 Hz (H5); δ = 3.27 ppm, dd, 1 H, J = 10.8, 6.2 Hz (H6a); δ = 3.18 ppm, dd, 1 H, J = 10.9, 2.0 Hz (H6b); δ = 2.22 ppm, s, 3 H ($OCOCH_3$); δ = 2.01 ppm, s, 3 H ($OCOCH_3$); δ = 1.77 ppm, s, 3 H ($OCOCH_3$).

^{13}C NMR (125.8 MHz, $CDCl_3$): δ = 170.0 ppm, 1 C ($OCOCH_3$); δ = 169.7 ppm, 1 C ($OCOCH_3$); δ = 169.4 ppm, 1 C ($OCOCH_3$); δ = 166.8 ppm, 1 C (carbonyl C of biphenyl); δ = 153.2 ppm, 1 C (aromatic C of biphenyl); δ = 143.6 ppm, 3 C (aromatic C of OTrt); δ = 143.5 ppm, 1 C (aromatic C of biphenyl); δ = 134.3 ppm, 1 C (aromatic C of biphenyl); δ = 131.9 ppm, 1 C (aromatic C of biphenyl); δ = 130.3 ppm, 2 C (aromatic C of biphenyl); δ = 129.3 ppm, 1 C (aromatic C of biphenyl); δ = 128.6 ppm, 6 C (aromatic C of OTrt); δ = 127.8 ppm, 6 C (aromatic C of OTrt); δ = 127.0 ppm, 3 C (aromatic C of OTrt); δ = 126.8 ppm, 2 C (aromatic C of biphenyl); δ = 126.0 - 125.8 ppm, m, 1 C (aromatic C of biphenyl); δ = 123.2 ppm, q, 1 C, J = 272.4 Hz (CF_3); δ = 120.6 ppm, q, 1 C, J = 31.6 Hz (aromatic C of biphenyl); δ = 116.6 ppm, 1 C (aromatic C of biphenyl); δ = 95.6 ppm, 1 C (C1); δ = 86.8 ppm, 1 C (aliphatic C, OTrt); δ = 71.5 ppm, 1 C (C5); δ = 69.4 ppm, 1 C (C2); δ = 68.8 ppm, 1 C (C3); δ = 66.1 ppm, 1 C (C4); δ = 62.4 ppm, 1 C (C6); δ = 52.2 ppm, 1 C (OCH_3); δ = 20.9 ppm, 1 C ($OCOCH_3$); δ = 20.7 ppm, 1 C ($OCOCH_3$); δ = 20.5 ppm, 1 C ($OCOCH_3$).

R_f = 0.43 with PE:EtOAc (2:1).

$[\alpha]^{20}_D$ = +67.7° (c = 1.00, $CHCl_3$).

ESI-MS for $C_{46}H_{41}F_3O_{11}$: $m \cdot z^{-1}(M+Na^+)_{calc}$ = 849.80; $m \cdot z^{-1}(M+Na^+)_{obs}$ = 849.37.

Methyl 4'-(2'',3'',4''-tri-O-acetyl-α-D-mannopyranosyloxy)-3'-trifluoromethylbiphenyl-4-carboxylate

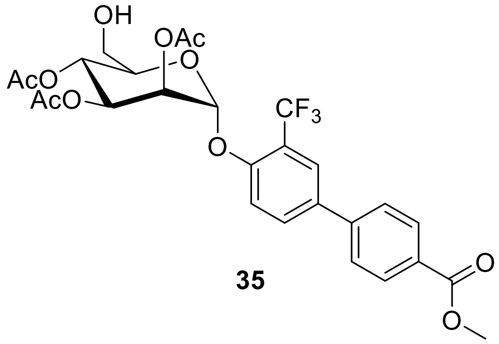

35

34 (454 mg, 549 µmol) was dissolved in DCM (20 mL). Next, $FeCl_3$ (178 mg, 1.10 mmol) and H_2O (120 µL, 6.59 mmol) were added. The reaction mixture was stirred for 5 h at room temperature. Product formation was monitored *via* TLC (1:2 (PE:EtOAc). The reaction mixture was diluted with DCM (40 mL) and the organic phase was extracted with H_2O (40 mL) and dried with Na_2SO_4. Solvents were evaporated *in vacuo* and the residue was purified *via* MPLC (gradient: 100% PE to 100% EtOAc in 15 min) to yield **35** as a white solid (223 mg, 382 µmol, 69%).

^{1}H NMR (500.0 MHz, $CDCl_3$): δ = 8.14 – 8.10 ppm, m, 2 H (aromatic H of biphenyl); δ = 7.87 – 7.85 ppm, m, 1 H (aromatic H of biphenyl); δ = 7.77 – 7.72 ppm, m, 1 H (aromatic H of biphenyl); δ = 7.64 – 7.58 ppm, m, 2 H (aromatic H of biphenyl); δ = 7.34 ppm, d, 1 H, J = 9.0 Hz (aromatic H of biphenyl); δ = 5.77 – 5.73 ppm, m, 1 H (H1); δ = 5.64 ppm, dd, 1 H, J = 10.2, 3.4 Hz (H3); δ = 5.53 – 5.50 ppm, m, 1 H (H2); δ = 5.42 – 5.36 ppm, m, 1 H (H4); δ = 3.95 ppm, s, 3 H (OC*H_3*); δ = 3.88 – 3.83 ppm, m, 1 H (H5); δ = 3.73 – 3.66 ppm, m, 1 H (H6a); δ = 3.65 – 3.57 ppm, m, 1 H (H6b); δ = 2.21 ppm, s, 3 H (OCOC*H_3*); δ = 2.11 ppm, s, 3 H (OCOC*H_3*); δ = 2.06 ppm, s, 3 H (OCOC*H_3*).

^{13}C NMR (125.8 MHz, $CDCl_3$): δ = 171.2 ppm, 1 C (O*C*OCH_3); δ = 170.9 ppm, 1 C (O*C*OCH_3); δ = 169.6 ppm, 1 C (O*C*OCH_3); δ = 166.8 ppm, 1 C (carbonyl C of biphenyl); δ = 152.9 ppm, 1 C (aromatic C of biphenyl); δ = 143.4 ppm, 1 C (aromatic C of biphenyl); δ = 134.4 ppm, 1 C (aromatic C of biphenyl); δ = 132.0 ppm, 1 C (aromatic C of biphenyl); δ = 130.3 ppm, 2 C (aromatic C of biphenyl); δ = 129.4 ppm, 1 C (aromatic C of biphenyl); δ = 126.8 ppm, 2 C (aromatic C of biphenyl); δ = 126.2 - 126.0 ppm, m, 1 C (aromatic C of biphenyl); δ = 123.1 ppm, q, 1 C, J = 272.5 Hz (*C*F_3); δ = 120.4 ppm, q, 1 C, J = 31.8 Hz (aromatic C of biphenyl); δ = 115.5 ppm, 1 C (aromatic C of biphenyl); δ = 95.6 ppm, 1 C (C1); δ = 72.0 ppm, 1 C (C5); δ = 69.2 ppm, 1 C (C2); δ = 68.3 ppm, 1 C (C3); δ = 65.9 ppm, 1 C (C4); δ = 60.9 ppm, 1 C (C6); δ = 52.2 ppm, 1 C (O*C*H_3); δ = 20.9 ppm, 1 C (OCO*C*H_3); δ = 20.8 ppm, 1 C (OCO*C*H_3); δ = 20.7 ppm, 1 C (OCO*C*H_3).

R_f = 0.53 with EtOAc:PE (2:1).

$[\alpha]^{20}_D$ = +62.1° (c = 1.00, $CHCl_3$).

ESI-MS for $C_{27}H_{27}F_3O_{11}$: $m{\cdot}z^{-1}(M+Na^+)_{calc}$ = 607.14; $m{\cdot}z^{-1}(M+Na^+)_{obs}$ = 607.10.

Methyl 4'-(2'',3'',4''-tri-O-acetyl-6''-O-tosyl-α-D-mannopyranosyloxy)-3'-trifluoromethylbiphenyl-4-carboxylate

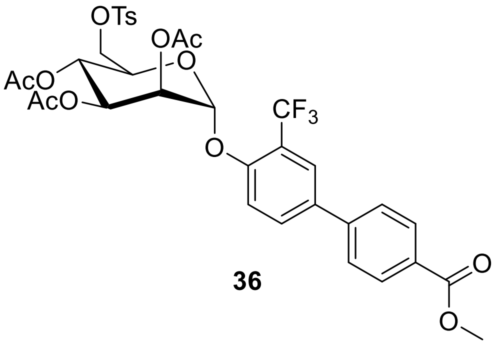

36

35 (216 mg, 370 µmol) was dissolved in dry pyridine (4 mL) and tosyl chloride (106 mg, 554 µmol) and a small amount of DMAP was added at 0°C. After 4 h more tosyl chloride (70.6 mg, 247 µmol) was added at 0°C. Next, the reaction mixture was stirred overnight at room temperature. Product formation was monitored *via* TLC (1:1 (PE:EtOAc)). Solvents were evaporated *in vacuo*. The residue was dissolved in DCM (50 mL) and the organic phase was extracted with 0.1 M HCl (50 mL), sat. $NaHCO_3$ (50 mL) and H_2O (50 mL). Subsequently, the organic phase was dried with Na_2SO_4 and solvents were evaporated *in vacuo*. The residue was purified *via* MPLC (gradient: 100% PE to 100% EtOAc in 20 min) to yield **36** as a white solid (183 mg, 248 µmol, 67%).

^{1}H NMR (500.0 MHz, $CDCl_3$): δ = 8.15 – 8.11 ppm, m, 2 H (aromatic H of biphenyl); δ = 7.85 ppm, d, 1 H, J = 2.0 Hz (aromatic H of biphenyl); δ = 7.74 – 7.68 ppm, m, 3 H (aromatic H of OTs, biphenyl); δ = 7.65 – 7.59 ppm, m, 2 H (aromatic H of biphenyl); δ = 7.33 – 7.27 ppm, m, 3 H (aromatic H of OTs, biphenyl); δ = 5.60 ppm, d, 1 H, J = 1.6 Hz (H1); δ = 5.50 ppm, dd, 1 H, J = 10.0, 3.4 Hz (H3); δ = 5.53 – 5.50 ppm, dd, 1 H, J = 3.5, 1.9 Hz (H2); δ = 5.33 – 5.26 ppm, m, 1 H (H4); δ = 4.17 ppm, dd, 1 H, J = 11.3, 5.9 Hz (H6a); δ = 4.12 ppm, dd, 1 H, J = 11.3, 2.4 Hz (H6b); δ = 4.04 ppm, ddd, 1 H, J = 10.0, 5.9, 2.5 Hz (H5); δ = 3.95 ppm, s, 3 H (OCH_3); δ = 2.41 ppm, s, 3 H (aliphatic H, OTs); δ = 2.19 ppm, s, 3 H (OCOCH_3); δ = 2.03 ppm, s, 3 H (OCOCH_3); δ = 2.00 ppm, s, 3 H (OCOCH_3).

^{13}C NMR (125.8 MHz, $CDCl_3$): δ = 169.9 ppm, 1 C (O*C*OCH_3); δ = 169.6 ppm, 1 C (O*C*OCH_3); δ = 169.6 ppm, 1 C (O*C*OCH_3); δ = 166.8 ppm, 1 C (carbonyl C of biphenyl); δ = 152.9 ppm, 1 C (aromatic C of biphenyl); δ = 145.1 ppm, 1 C (aromatic C of OTs); δ = 143.4 ppm, 1 C (aromatic C of biphenyl); δ = 134.7 ppm, 1 C (aromatic C of biphenyl); δ = 132.7 ppm, 1 C (aromatic C of OTs); δ = 132.0 ppm, 1 C (aromatic C of biphenyl); δ = 130.3 ppm, 2 C (aromatic C of biphenyl); δ = 129.8 ppm, 2 C (aromatic C of OTs); δ = 129.4 ppm, 1 C (aromatic C of biphenyl); δ = 128.0 ppm, 2 C (aromatic C of OTs); δ = 126.8 ppm, 2 C (aromatic C of biphenyl); δ = 126.0 ppm, q, 1 C, J = 5.1 Hz (aromatic C of biphenyl); δ = 123.1 ppm, q, 1 C, J = 272.5 Hz (*C*F_3); δ = 120.5 ppm, q, 1 C, J = 31.8 Hz (aromatic C of biphenyl); δ = 116.1 ppm, 1 C (aromatic

C of biphenyl); δ = 95.7 ppm, 1 C (C1); δ = 69.6 ppm, 1 C (C5); δ = 69.0 ppm, 1 C (C2); δ = 68.4 ppm, 1 C (C3); δ = 67.6 ppm, 1 C (C6); δ = 65.6 ppm, 1 C (C4); δ = 52.2 ppm, 1 C (O*C*H_3); δ = 21.6 ppm, 1 C (aliphatic C, OTs); δ = 20.8 ppm, 1 C (OCO*C*H_3); δ = 20.6 ppm, 1 C (OCO*C*H_3); δ = 20.6 ppm, 1 C (OCO*C*H_3).

R_f = 0.65 with EtOAc:PE (1:1).

$[\alpha]^{20}_D$ = +58.4° (c = 1.00, $CHCl_3$).

ESI-MS for $C_{34}H_{33}F_3O_{13}S$: $m \cdot z^{-1}(M+Na^+)_{calc}$ = 761.15; $m \cdot z^{-1}(M+Na^+)_{obs}$ = 761.17.

Methyl 4'-(2'',3'',4''-tri-O-acetyl-6''-deoxy-6''-azido-α-D-mannopyranosyloxy)-3'-trifluoromethylbiphenyl-4-carboxylate

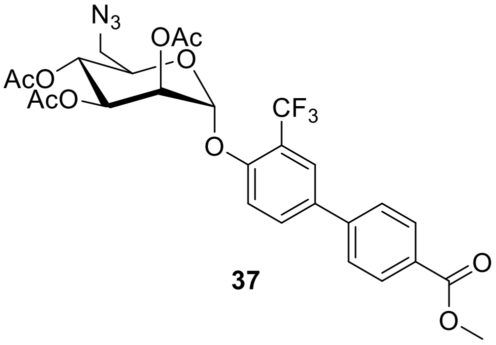

37

36 (30 mg, 41 µmol), sodium azide (14 mg, 203 µmol) and 1,4,7,10,13,16-hexaoxacyclooctadecane (6 mg, 20 µmol) were dissolved in anhydrous DMF (2 mL) at room temperature under argon atmosphere. The reaction mixture was stirred at 80°C overnight and subsequently diluted with diethyl ether (25 mL). The organic phase was extracted with H_2O (25 mL) and dried over Na_2SO_4. Solvents were evaporated *in vacuo* and the residue was purified *via* MPLC (gradient: 100% PE to 100% EtOAc in 20 min) to yield **37** (20.4 mg, 33 µmol, 82%) as a light orange solid.

^{1}H NMR (500.0 MHz, $CDCl_3$): δ = 8.15 – 8.09 ppm, m, 2 H (aromatic H of biphenyl); δ = 7.87 ppm, d, 1 H, J = 2.0 Hz (aromatic H of biphenyl); δ = 7.78 ppm, dd, 1 H, J = 8.9, 2.1 Hz (aromatic H of biphenyl); δ = 7.65 – 7.59 ppm, m, 2 H (aromatic H of biphenyl); δ = 7.37 ppm, d, 1 H, J = 8.8 Hz (aromatic H of biphenyl); δ = 5.72 ppm, d, 1 H, J = 1.7 Hz (H1); δ = 5.57 ppm, dd, 1 H, J = 10.9, 3.4 Hz (H3); δ = 5.50 ppm, dd, 1 H, J = 3.5, 1.9 Hz (H2); δ = 5.41 – 5.35 ppm, m, 1 H (H4); δ = 4.08 – 4.02 ppm, m, 1 H (H5); δ = 3.95 ppm, s, 3 H (OC*H_3*); δ = 3.39 ppm, dd, 1 H, J = 13.3, 6.6 Hz (H6a); δ = 3.27 ppm, dd, 1 H, J = 13.4, 2.5 Hz (H6b); δ = 2.23 ppm, s, 3 H (OCOC*H_3*); δ = 2.07 ppm, s, 3 H (OCOC*H_3*); δ = 2.05 ppm, s, 3 H (OCOC*H_3*).

^{13}C NMR (125.8 MHz, $CDCl_3$): δ = 169.9 ppm, 1 C (O*C*OCH_3); δ = 169.8 ppm, 1 C (O*C*OCH_3); δ = 169.6 ppm, 1 C (O*C*OCH_3); δ = 166.8 ppm, 1 C (carbonyl C of biphenyl); δ = 152.8 ppm, 1 C (aromatic C of biphenyl); δ = 143.4 ppm, 1 C (aromatic C of biphenyl); δ = 134.6 ppm, 1 C (aromatic C of biphenyl); δ = 132.0 ppm, 1 C

(aromatic C of biphenyl); δ = 130.3 ppm, 2 C (aromatic C of biphenyl); δ = 129.4 ppm, 1 C (aromatic C of biphenyl); δ = 126.8 ppm, 2 C (aromatic C of biphenyl); δ = 126.1 ppm, q, 1 C, J = 5.1 Hz (aromatic C of biphenyl); δ = 123.2 ppm, q, 1 C, J = 272.8 Hz (CF_3); δ = 120.4 ppm, q, 1 C, J = 31.8 Hz (aromatic C of biphenyl); δ = 115.6 ppm, 1 C (aromatic C of biphenyl); δ = 95.3 ppm, 1 C (C1); δ = 71.5 ppm, 1 C (C5); δ = 69.1 ppm, 1 C (C2); δ = 68.3 ppm, 1 C (C3); δ = 66.6 ppm, 1 C (C4); δ = 52.2 ppm, 1 C (OCH_3); δ = 51.0 ppm, 1 C (C6); δ = 20.9 ppm, 1 C ($OCOCH_3$); δ = 20.7 ppm, 1 C ($OCOCH_3$); δ = 20.6 ppm, 1 C ($OCOCH_3$).

R_f = 0.56 with PE:EtOAc (2:1).

$[\alpha]^{20}_D$ = +35.8° (c = 1.00, $CHCl_3$).

ESI-MS for $C_{27}H_{26}F_3N_3O_{10}$: $m{\cdot}z^{-1}(M+Na^+)_{calc}$ = 632.15; $m{\cdot}z^{-1}(M+Na^+)_{obs}$ = 632.25.

ATR-FTIR (selected resonances): ν = 2104.6 cm^{-1} (azide stretching).

Methyl 4’-(2’’,3’’,4’’-tri-O-acetyl-6’’-deoxy-6’’-2’’’-(phthalimido)ethylsulfonamido-α-D-mannopyranosyloxy)-3’-trifluoromethylbiphenyl-4-carboxylate

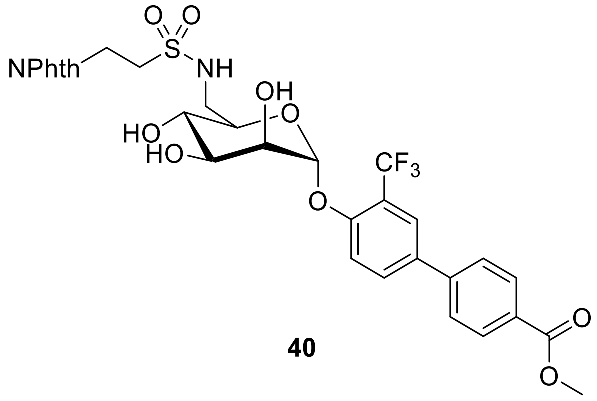

40

37 (101 mg, 166 µmol) was dissolved in anhydrous MeOH (5 mL) and 1 M MeONa (30 µL, 30 µmol). The reaction mixture was stirred for 4 h at room temperature under argon atmosphere. Product formation was monitored *via* TLC (PE:EA (1:2)). The pH was adjusted to 6 to 7 using Amberlite IR120 (H^+) and solvents were removed *in vacuo*.

Next, the residue was dissolved in 1,4-dioxane (3 mL) and Pd/C (40 mg) was added under argon atmosphere in a twin-neck flask. The flask was flushed with H_2 at atmospheric pressure. The reaction mixture was stirred for overnight at room temperature. Product formation was monitored *via* TLC (2:1 (PE:EtOAc)) and ESI-MS. Pd/C was removed by Celite filtration and solvents were removed *in vacuo*.

The residue was dissolved in anhydrous DMF (1.5 mL) and Et_3N (25 µL, 174 µmol) was added. 2-(phthalimido)ethanesulfonyl chloride (30 mg, 104 µmol) dissolved in DMF (750 µL) was slowly added under argon atmosphere at 0°C. The reaction mixture was stirred for 1 h at 0°C, allowed to heat up to room temperature and stirred for an additional 48 h. Solvents were evaporated *in vacuo* and the residue was purified *via*

reversed-phase MPLC (gradient: 100% H2O to 100% MeOH, elution at 100% MeOH) to yield **40** (34 mg, 49 µmol, 41% over 3 steps) as a white solid.

^{1}H NMR (500.0 MHz, MeOD): δ = 8.03 – 7.98 ppm, m, 2 H (aromatic H of biphenyl); δ = 7.87 ppm, dd, 1 H, J = 8.6, 2.3 Hz (aromatic H of biphenyl); δ = 7.76 – 7.72 ppm, m, 5 H (aromatic H of biphenyl, NPhth); δ = 7.65 – 7.59 ppm, m, 3 H (aromatic H of biphenyl); δ = 5.73 ppm, d, 1 H, J = 1.5 Hz (H1); δ = 4.08 ppm, dd, 1 H, J = 3.5, 1.8 Hz (H2); δ = 4.03 – 3.87 ppm, m, 6 H (NCH_2CH$_2$S, H3, OCH_3); δ = 3.74 – 3.69 ppm, m, 1 H (H4); δ = 3.67 – 3.62 ppm, m, 1 H (H5); δ = 3.39 ppm, dd, 1 H, J = 14.1, 2.4 Hz (H6a); δ = 3.38 – 3.35 ppm, m, 3 H, J = 13.4, 2.5 Hz (H6b, NCH$_2$CH_2S).

^{13}C NMR (125.8 MHz, MeOD): δ = 169.9 ppm, 2 C (carbonyl C of NPhth); δ = 168.2 ppm, 1 C (carbonyl C of biphenyl); δ = 155.1 ppm, 1 C (aromatic C of biphenyl); δ = 143.8 ppm, 1 C (aromatic C of biphenyl); δ = 135.4 ppm, 2 C (aromatic C of NPhth); δ = 134.6 ppm, 1 C (aromatic C of biphenyl); δ = 133.5 ppm, 1 C (aromatic C of biphenyl); δ = 133.2 ppm, 2 C (aromatic C of NPhth); δ = 131.2 ppm, 2 C (aromatic C of biphenyl); δ = 130.3 ppm, 1 C (aromatic C of biphenyl);); δ = 127.7 ppm, 2 C (aromatic C of biphenyl); δ = 126.4 ppm, q, 1 C, J = 5.2 Hz (aromatic C of biphenyl); δ = 125.0 ppm, q, 1 C, J = 272.2 Hz (*C*F$_3$); δ = 124.2 ppm, 2 C (aromatic C of NPhth); δ = 120.8 ppm, q, 1 C, J = 30.8 Hz (aromatic C of biphenyl); δ = 117.7 ppm, 1 C (aromatic C of biphenyl); δ = 99.8 ppm, 1 C (C1); δ = 74.7 ppm, 1 C (C5); δ = 71.9 ppm, 1 C (C3); δ = 71.6 ppm, 1 C (C2); δ = 69.1 ppm, 1 C (C4); δ = 52.7 ppm, 1 C (O*C*H$_3$); δ = 52.7 ppm, 1 C (NCH$_2$*C*H$_2$S); δ = 45.0 ppm, 1 C (C6); δ = 33.7 ppm, 1 C (N*C*H$_2$CH$_2$S).

R_f = 0.61 with 5% MeOH in DCM.

$[\alpha]^{20}_{D}$ = +57.8° (c = 1.00, MeOH).

ESI-MS for $C_{31}H_{29}F_3N_2O_{11}S$: $m\cdot z^{-1}(M\text{-}H^+)_{calc}$ = 693.14; $m\cdot z^{-1}(M\text{-}H^+)_{obs}$ = 693.31.

4’-(6’’-(2’’’-Aminoethylsulfonamido)-6’’-deoxy-α-D-mannopyranosyloxy)-3’-trifluoromethylbiphenyl-4-carboxylic acid

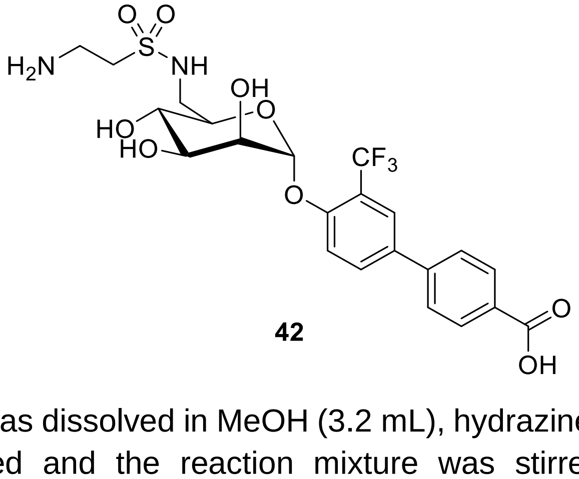

42

40 (33 mg, 48 µmol) was dissolved in MeOH (3.2 mL), hydrazine monohydrate (33 µL, 665 µmol) was added and the reaction mixture was stirred overnight at room temperature. Product formation was analyzed *via* MS and TLC (5% MeOH in DCM).

Next, 2 M aqueous NaOH (340 µL, 665 µmol) was added and the reaction mixture was stirred at room temperature for 4 h. More 2 M NaOH (340 µL, 665 µmol) was added and reaction mixture was stirred at room temperature overnight. Product formation was analyzed *via* ESI-MS. Solvents were evaporated *in vacuo* and the residue was purified *via* reversed-phase MPLC (gradient: 100% H_2O to 100% MeOH in 30 min) to yield **42** (12.3 mg, 22 µmol, 47% over 2 steps) as a white solid after lyophilization from H_2O.

^{1}H NMR (700.0 MHz, D_2O): δ = 8.04 – 8.00 ppm, m, 1 H (aromatic H of biphenyl); δ = 7.98 – 7.92 ppm, m, 3 H (aromatic H of biphenyl); δ = 7.76 – 7.70 ppm, m, 2 H (aromatic H of biphenyl); δ = 7.51 ppm, d, 1 H, J = 8.9 Hz (aromatic H of biphenyl); δ = 5.88 – 5.84 ppm, m, 1 H (H1); δ = 4.27 – 4.23 ppm, m, 1 H (H2); δ = 4.06 ppm, dd, 1 H, J = 9.5, 3.5 Hz (H3); δ = 3.71 – 3.66 ppm, m, 1 H (H4); δ = 3.65 – 3.60 ppm, m, 1 H (H5); δ = 3.53 ppm, dd, 1 H, J = 14.8, 2.1 Hz (H6a); δ = 3.25 ppm, dd, 1 H, J = 14.5, 8.1 Hz (H6b); δ = 3.12 – 2.98 ppm, m, 2 H (NCH_2CH_2S); δ = 2.86 – 2.80 ppm, m, 2 H (NCH_2CH_2S).

^{13}C NMR (176.0 MHz, D_2O): δ = 175.3 ppm, 1 C (carbonyl C of biphenyl); δ = 160.9 ppm, 1 C (aromatic C of biphenyl); δ = 152.1 ppm, 1 C (aromatic C of biphenyl); δ = 141.3 ppm, 1 C (aromatic C of biphenyl); δ = 135.4 ppm, 1 C (aromatic C of biphenyl); δ = 133.8 ppm, 1 C (aromatic C of biphenyl); δ = 132.1 ppm, 1 C (aromatic C of biphenyl); δ = 129.4 ppm, 2 C (aromatic C of biphenyl); δ = 126.5 ppm, 2 C (aromatic C of biphenyl); δ = 125.8 ppm, 1 C (aromatic C of biphenyl); δ = 123.5 ppm, q, 1 C, J = 272.4 Hz (CF_3); δ = 119.1 ppm, q, 1 C, J = 30.9 Hz (aromatic C of biphenyl); δ = 116.1 ppm, 1 C (aromatic C of biphenyl); δ = 96.7 ppm, 1 C (C1); δ = 72.7 ppm, 1 C (C5); δ = 70.1 ppm, 1 C (C3); δ = 69.6 ppm, 1 C (C2); δ = 67.8 ppm, 1 C (C4); δ = 54.2 ppm, 1 C (NCH_2CH_2S); δ = 43.1 ppm, 1 C (C6); δ = 35.3 ppm, 1 C (NCH_2CH_2S).

R_f = no migration with DCM:MeOH:aqueous NH_4OH (8:2:0.4).

$[\alpha]^{20}_D$ = +71.6° (c = 0.10, H_2O).

HR ESI-MS for $C_{22}H_{25}F_3N_2O_9S$: $m\cdot z^{-1}(M+H^+)_{calc}$ = 551.131; $m\cdot z^{-1}(M+H^+)_{obs}$ = 551.132.

4’-(6’’-(2’’’-Acetamidoethylsulfonamido)-6’’-deoxy-α-D-mannopyranosyloxy)-3’-trifluoromethylbiphenyl-4-carboxylic acid

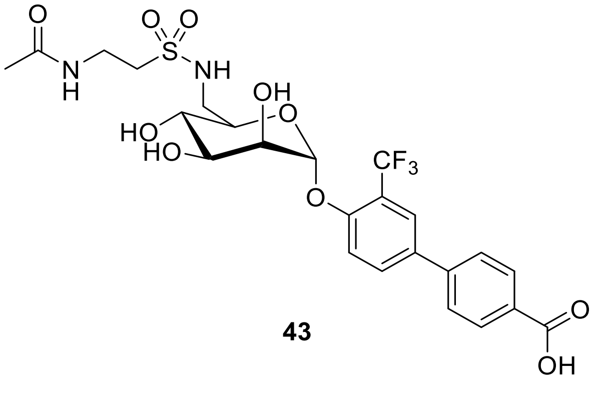

43

42 (5.2 mg, 9.5 µmol) was dissolved in MeOH (1 mL) and acetic anhydride (5.4 µL, 57 µmol) was added dropwise at room temperature under argon atmosphere. The reaction mixture was stirred for 4 h. Product formation was monitored *via* ESI-MS and TLC (10% MeOH in DCM). Solvents were evaporated *in vacuo* and the residue was purified *via* reversed-phase MPLC (gradient 100% H_2O to 100% MeOH in 35 min) to yield **43** (3.98 mg, 6.7 µmol, 70.7%) as a white solid after lyophilization from H_2O. A purity higher than 95% was demonstrated *via* analytical reversed-phase HPLC.

^{1}H NMR (500.0 MHz, D_2O): δ = 8.00 – 7.90 ppm, m, 4 H (aromatic H of biphenyl); δ = 7.74 – 7.68 ppm, m, 2 H (aromatic H of biphenyl); δ = 7.47 ppm, d, 1 H, J = 8.8 Hz (aromatic H of biphenyl); δ = 5.85 ppm, d, 1 H, J = 1.5 Hz (H1); δ = 4.24 ppm, dd, 1 H, J = 3.6, 1.8 Hz (H2); δ = 4.05 ppm, dd, 1 H, J = 9.6, 3.5 Hz (H3); δ = 2.67 – 2.61 ppm, m, 1 H (H4); δ = 3.60 – 3.53 ppm, m, 2 H (H5, H6a); δ = 3.23 ppm, dd, 1 H, J = 15.6, 9.3 Hz (H6b); δ = 3.19 – 3.00 ppm, m, 2 H (NCH_2CH$_2$S); δ = 3.09 – 2.86 ppm, m, 2 H (NCH$_2$CH_2S); δ = 1.70 ppm, s, 3 H (NCOCH_3).

^{13}C NMR (125.7 MHz, D_2O): δ = 175.2 ppm, 1 C (carbonyl C of biphenyl); δ = 173.7 ppm, 1 C (N*C*OCH$_3$); δ = 161.3 ppm, 1 C (aromatic C of biphenyl); δ = 151.8 ppm, 1 C (aromatic C of biphenyl); δ = 141.1 ppm, 1 C (aromatic C of biphenyl); δ = 135.3 ppm, 1 C (aromatic C of biphenyl); δ = 133.5 ppm, 1 C (aromatic C of biphenyl); δ = 131.9 ppm, 1 C (aromatic C of biphenyl); δ = 129.6 ppm, 2 C (aromatic C of biphenyl); δ = 126.3 ppm, 2 C (aromatic C of biphenyl); δ = 125.6 ppm, q, 1 C, J = 5.4 Hz (aromatic C of biphenyl); δ = 123.5 ppm, q, 1 C, J = 272.0 Hz (*C*F$_3$); δ = 118.9 ppm, q, 1 C, J = 30.9 Hz (aromatic C of biphenyl); δ = 115.8 ppm, 1 C (aromatic C of biphenyl); δ = 96.3 ppm, 1 C (C1); δ = 72.8 ppm, 1 C (C5); δ = 70.0 ppm, 1 C (C3); δ = 69.5 ppm, 1 C (C2); δ = 67.9 ppm, 1 C (C4); δ = 51.3 ppm, 1 C (NCH$_2$*C*H$_2$S); δ = 43.2 ppm, 1 C (C6); δ = 33.5 ppm, 1 C (N*C*H$_2$CH$_2$S) δ = 21.4 ppm, 1 C (NCO*C*H$_3$).

R_f = 0.15 with 10% MeOH in DCM.

$[\alpha]^{20}_D$ = +74.8° (c = 0.05, H_2O).

HR ESI-MS for $C_{24}H_{27}F_3N_2O_{10}S$: $m\cdot z^{-1}(M+Na^+)_{calc}$ = 615.124; $m\cdot z^{-1}(M+Na^+)_{obs}$ = 615.125.

4'-(6''-Amino-6''-deoxy-α-D-mannopyranosyloxy)-3'-trifluoromethylbiphenyl-4-carboxylic acid

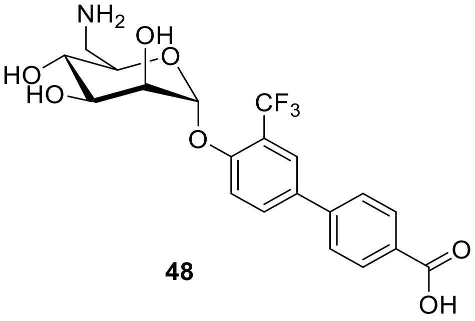

48

37 (0.35 g, 0.72 mmol) was dissolved in MeOH (10 mL) and 25% (w/w) MeONa solution (0.5 mL) was added. The reaction mixture was stirred for 1 h at room temperature under argon atmosphere in presence of trace H_2O for deprotection of the carboxylic acid and product formation was monitored via HPLC. Thereafter, the reaction mixture was neutralized using Amberlite IR120 (H^+) and solvents was removed *in vacuo*. Subsequently, the residue was dissolved in 1,4-dioxane (3 mL) and Pd/C (35 mg) was added under argon atmosphere. The flask was then flushed with H_2 at atmospheric pressure, stirred for 3 h at room temperature whilst product formation was monitored via UHPLC. Pd/C was removed by Celite filtration and the solvents were removed *in vacuo*. Purification by reversed-phase HPLC (gradient: 100% H_2O to 50% MeOH in 30 min) gave **48** as a white powder (0.24 g, 0.53 mmol, 74%). Purity was further monitored by analytical reversed-phase HPLC (**Figure S14**).

^1H NMR (700.0 MHz, D_2O): δ = 8.04 ppm, d, 1 H (aromatic H of biphenyl); δ = 7.99 – 7.94 ppm, dd, 3 H (aromatic H of biphenyl); δ = 7.78 – 7.74, m, 2 H (aromatic H of biphenyl); δ = 7.53, d, 1 H, J = 8.7 Hz) (aromatic H of biphenyl); δ = 5.89 ppm, d, 1 H, J = 1.8 Hz (H1); δ = 4.27 ppm, dd, 1 H, J = 3.5, 1.9 Hz (H2); δ = 4.08 ppm, dd, 1 H, J = 9.4, 3.5 Hz (H3); δ = 3.75 – 3.70, m, 2 H (H5); δ = 3.70 – 3.65, m, 1 H (H4); δ = 3.48 – 3.38 ppm, m, 2 H (H6a, H6b).

^{13}C NMR (125.7 MHz, DMSO): ^{13}C NMR (125.7 MHz, DMSO): δ = 171.4 ppm, 1 C (carbonyl C of biphenyl); δ = 168.3 ppm, 1 C (aromatic C of biphenyl); δ = 155.9 ppm, 1 C (aromatic C of biphenyl); δ = 140.9 ppm, 1 C (aromatic C of biphenyl); δ = 136.2 ppm, 1 C (aromatic C of biphenyl); δ = 134.5 ppm, 1 C (aromatic C of biphenyl); δ = 132.4 ppm, 1 C (aromatic C of biphenyl); δ = 129.6 ppm, 2 C (aromatic C of biphenyl); δ = 127.3 ppm, 2 C (aromatic C of biphenyl); δ = 125.3 ppm, q, 1 C, J = 5.4 Hz (aromatic C of biphenyl); δ = 123.1 ppm, q, 1 C, J = 272.0 Hz (*C*F_3); δ = 119.3 ppm, q, 1 C, J = 30.9 Hz (aromatic C of biphenyl); δ = 115.1 ppm, 1 C (aromatic C of biphenyl); δ = 101.4 ppm, 1 C (C1); δ = 73.1 ppm, 1 C (C5); δ = 69.1 ppm, 1 C (C3); δ = 69.0 ppm, 1 C (C2); δ = 67.9 ppm, 1 C (C4); δ = 63.6 ppm, 1 C (C6).

ESI-MS for $C_{20}H_{20}F_3NO_7$: $m{\cdot}z^{-1}(M+H^+)_{calc}$ = 444.13; $m{\cdot}z^{-1}(M+Na^+)_{obs}$ = 444.27.

3-Cysteamine-1-propyl α-L-fucopyranoside

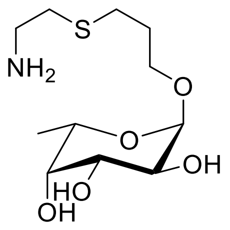

Fuc

51 was synthesized as previously reported.[20] **Fuc** was prepared following the protocol published by Sommer *et al.* for methylated mannosides.[25] Briefly, **51** (100 mg, 0.49 mmol) and cysteamine hydrochloride (556 mg, 4.9 mmol) were stirred at 50 °C in H_2O (5 mL) for 18 h. Upon full conversion, the reaction was stopped by evaporation *in vacuo*. Purification of the residue by MPLC (CH_2Cl_2/MeOH gradient 5 to 30%, supplemented with 1% aqueous ammonia 25% w/v) followed by a second MPLC purification on C_{18} silica (H_2O, 2% MeCN, 0.1% formic acid) yielded **Fuc** as an amorphous solid (37 mg, 0.105 mmol, 22%) after lyophilization. Despite a quantitative turnover, the yield was low due to difficulties associated with the molecule's high polarity.

^{1}H NMR (500.0 MHz, MeOD): δ = 4.79 – 4.68 ppm, m, 1 H (H1); δ = 3.94, q, 1 H, J = 1.3, 6.6 Hz (H5); δ = 3.79 ppm, ddd, 1 H (-$OCH_2CH_2CH_2S$-); δ = 3.76 – 3.71 ppm, m, 2 H (H2 and H3); δ = 3.70 – 3.64 ppm, m, 1 H (H4); δ = 3.52 ppm, ddd, 1 H, J = 5.3 Hz, 6.6, 10.0 Hz (-$OCH_2CH_2CH_2S$-); δ = 3.15 ppm, t, 2 H, J = 6.9 Hz (-$SC\mathit{H_2}CH_2NH_2$); δ = 2.89 – 2.78 ppm, m, 2 H (-$SCH_2C\mathit{H_2}NH_2$); δ = 2.72 ppm, td, 2 H, J = 1.7, 7.2 Hz (-$OCH_2CH_2CH_2S$-); δ = 2.10 – 1.78 ppm, m, 2 H (-$OCH_2CH_2CH_2S$-); δ = 1.21 ppm, d, 3 H, J = 6.6 Hz (H6).

^{13}C NMR (126 MHz, MeOD) δ = 99.05 ppm, 1 C (C1); δ = 72.20 ppm, 1 C, (C4); δ = 70.26 ppm, 1 C (C2 or C3); δ = 68.61 ppm, 1 C (C2 or C3); δ = 66.32 ppm, 1 C (C5); δ = 65.80 ppm, 1 C (CH_2); δ = 38.47 ppm, 1 C (CH_2); δ = 28.94 ppm, 1 C (CH_2); δ = 28.33 ppm, 1 C (CH_2); δ = 27.59 ppm, 1 C (CH_2); δ = 15.30 ppm, 1 C (C6).

ESI-MS for $C_{11}H_{23}NO_5S$: $m \cdot z^{-1}(M+H^+)_{calc}$ = 282.14; $m \cdot z^{-1}(M+H^+)_{obs}$ = 282.14.

Glycolipids – 42-Lip, 48-Lip, 50-Lip, Man-Lip, Fuc-Lip and LeX-Lip

Structures are shown in **Scheme S4**.

General procedure. DSPE-PEG_{2kDa}-NHS (NOF Europe) was dissolved in anhydrous DMF (to 0.65 mM) and anhydrous *N,N*-diisopropylethylamine (1 µL per mL DMF) was added. 5.5 equivalents of the targeting ligand bearing a primary amino group (stock

solution at 3.5 mM) were added and reaction mixtures was stirred for 18 h at room temperature. Solvents were removed *in vacuo* and the residue was dissolved in 0.1 M $NaHCO_3$ in H_2O and purified via dialysis (3 mL Slide-A-Lyzer cassette; twice against 2 L of 0.1 M $NaHCO_3$ for 3 h and subsequently twice against H_2O). Solvents were removed by lyophilization and the residue was dissolved in DMSO-d_6 to determine coupling efficiencies using ^1H NMR spectroscopy by integration of characteristic resonances (**Table S1_RW).**

Synthetic chemistry – ^1H and ^{13}C NMR Spectra

4'-Bromo-2'-(trifluoromethyl)phenyl α-D-mannopyranoside (31)

^1H NMR (500.0 MHz, MeOD)

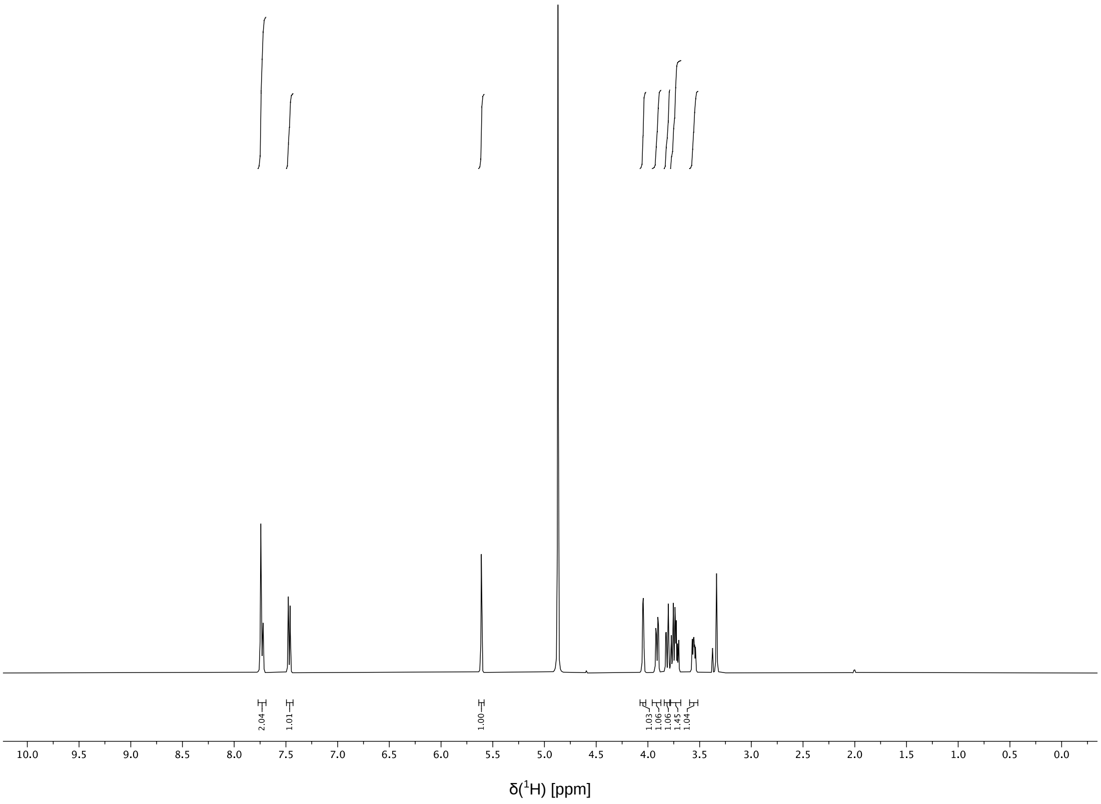

^{13}C NMR (125.8 MHz, MeOD)

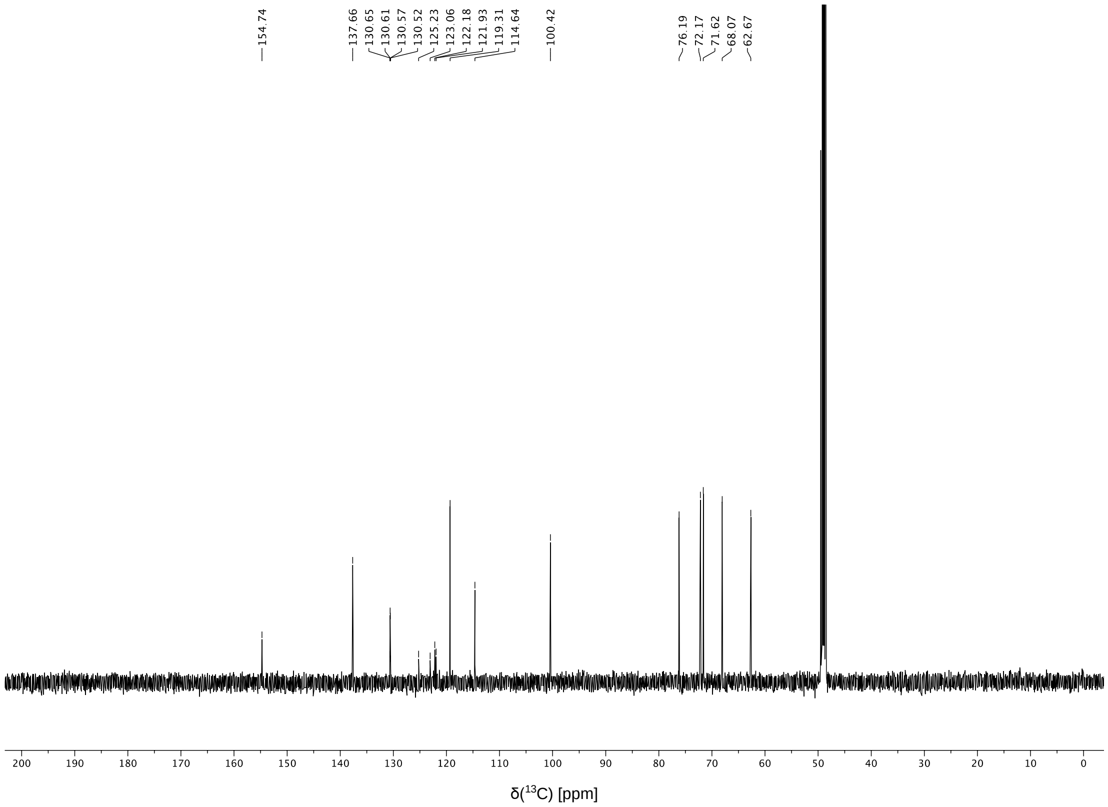

4’-Bromo-2’-(trifluoromethyl)phenyl 6-O-trityl-α-D-mannopyranoside (32)

^{1}H NMR (500.0 MHz, $CDCl_3$)

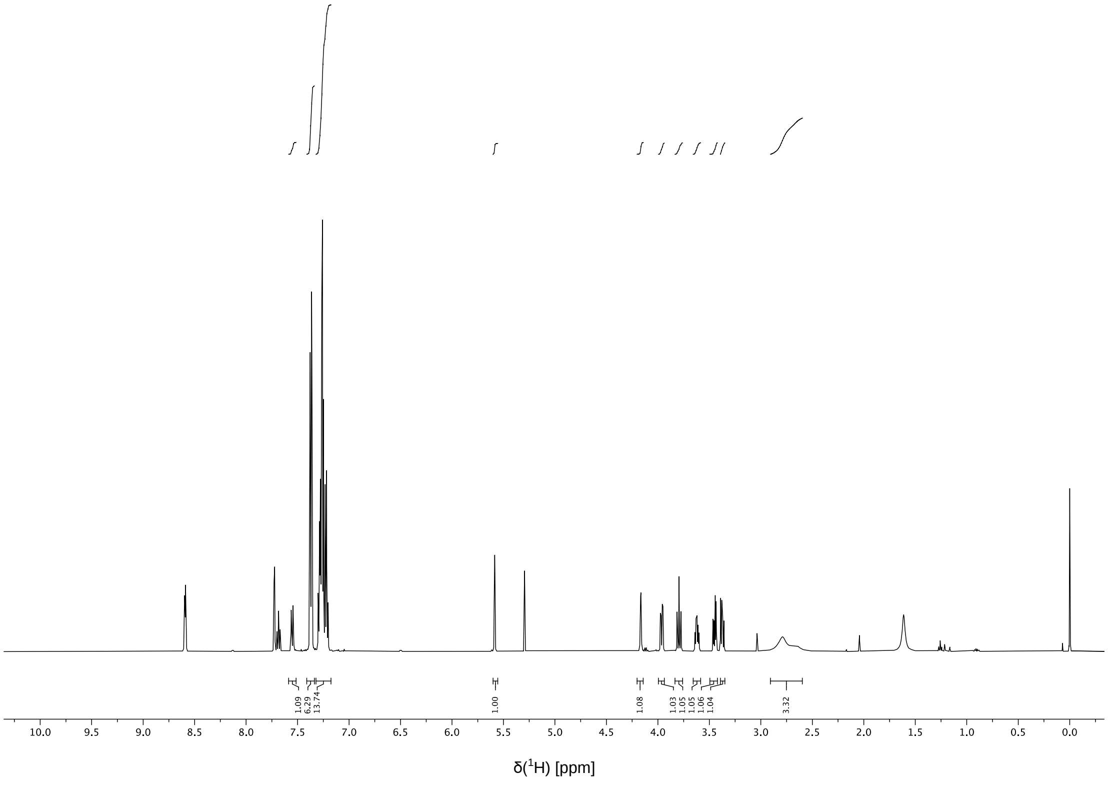

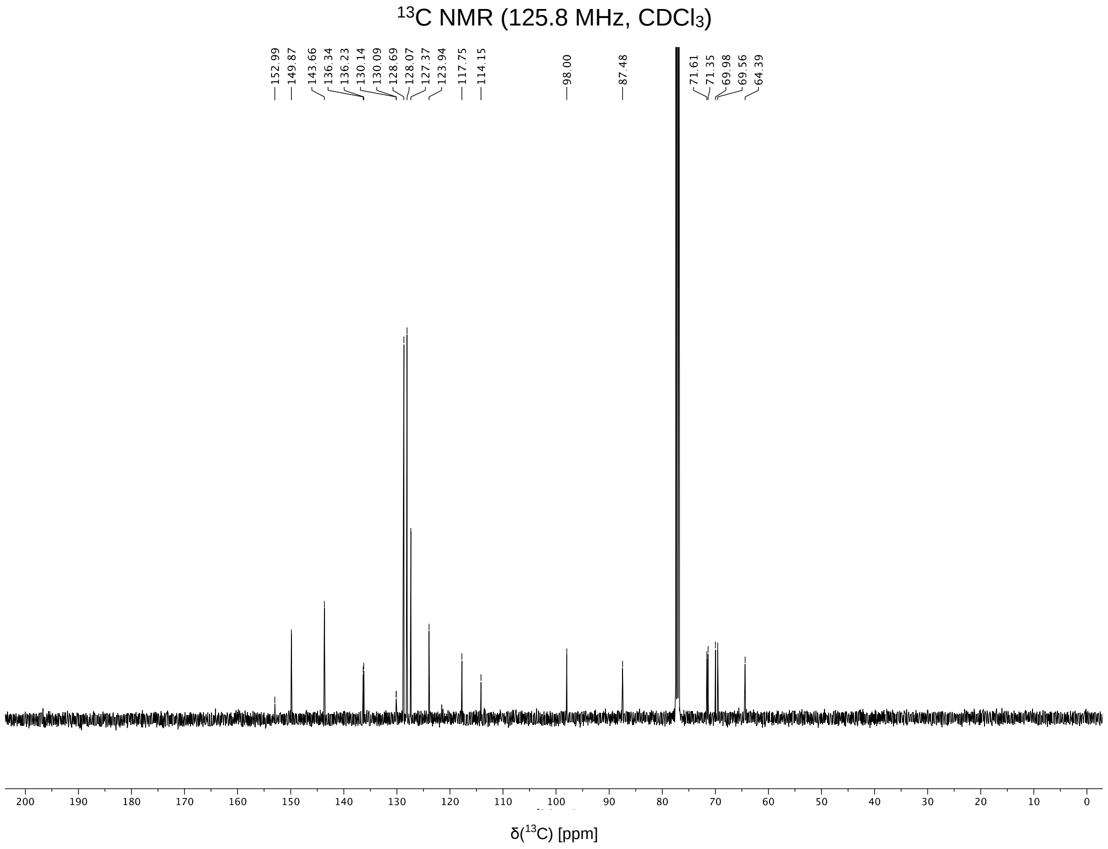

13C NMR (125.8 MHz, CDCl3)
152.99
149.87
143.66
136.34
136.23
130.14
130.09
128.69
128.07
127.37
123.94
117.75
114.15
98.00
87.48
71.61
71.35
69.98
69.56
64.39
200
190
180
170
160
150
140
130
120
110
100
90
80
70
60
50
40
30
20
10
0
δ(13C) [ppm]

4'-Bromo-2'-(trifluoromethyl)phenyl 2,3,4-tri-O-acetyl-6-O-trityl-α-D-mannopyranoside (33)

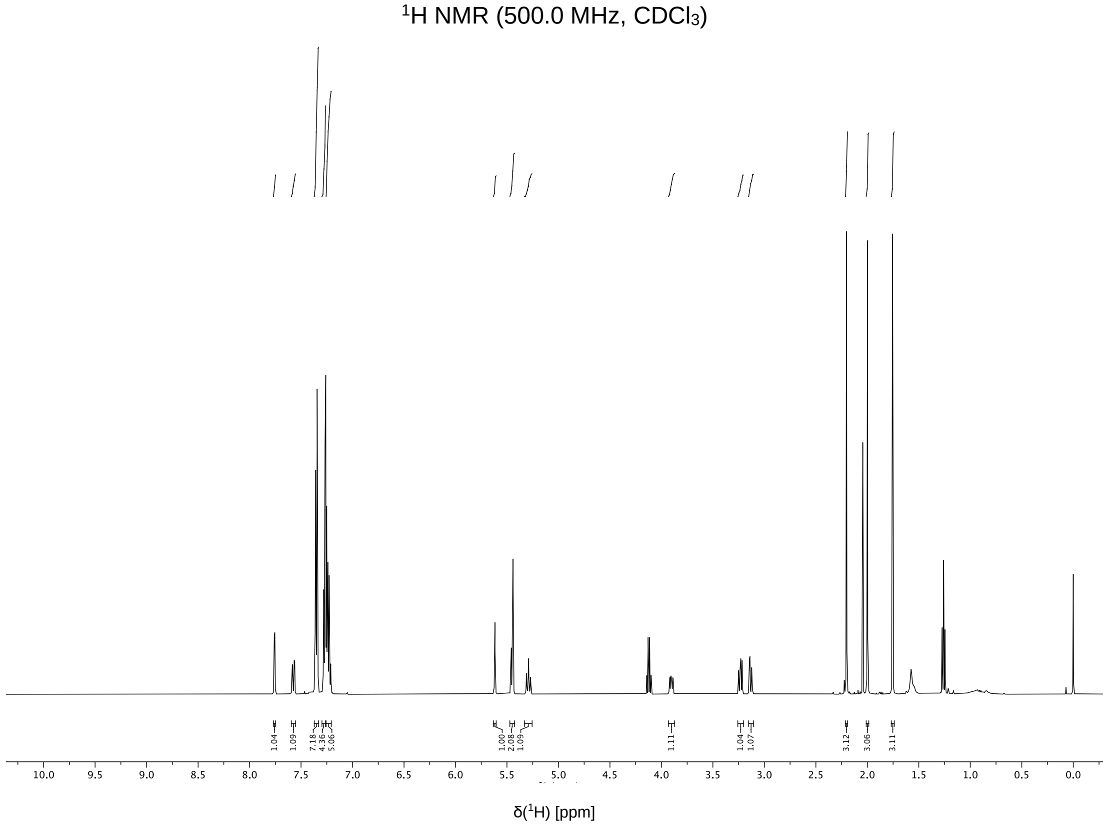

^{13}C NMR (125.8 MHz, $CDCl_3$)

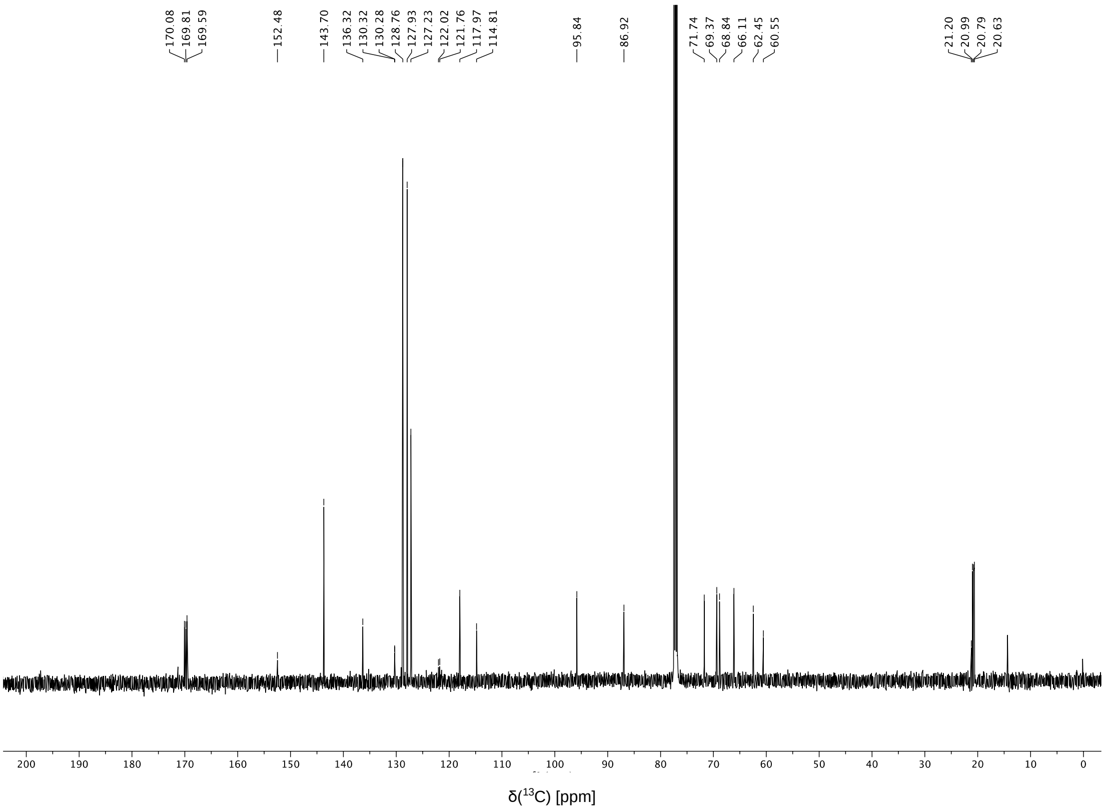

Methyl 4’-(2’’,3’’,4’’-tri-O-acetyl-6’’-O-trityl-α-D-mannopyranosyloxy)-3’-trifluoromethylbiphenyl-4-carboxylate (34)

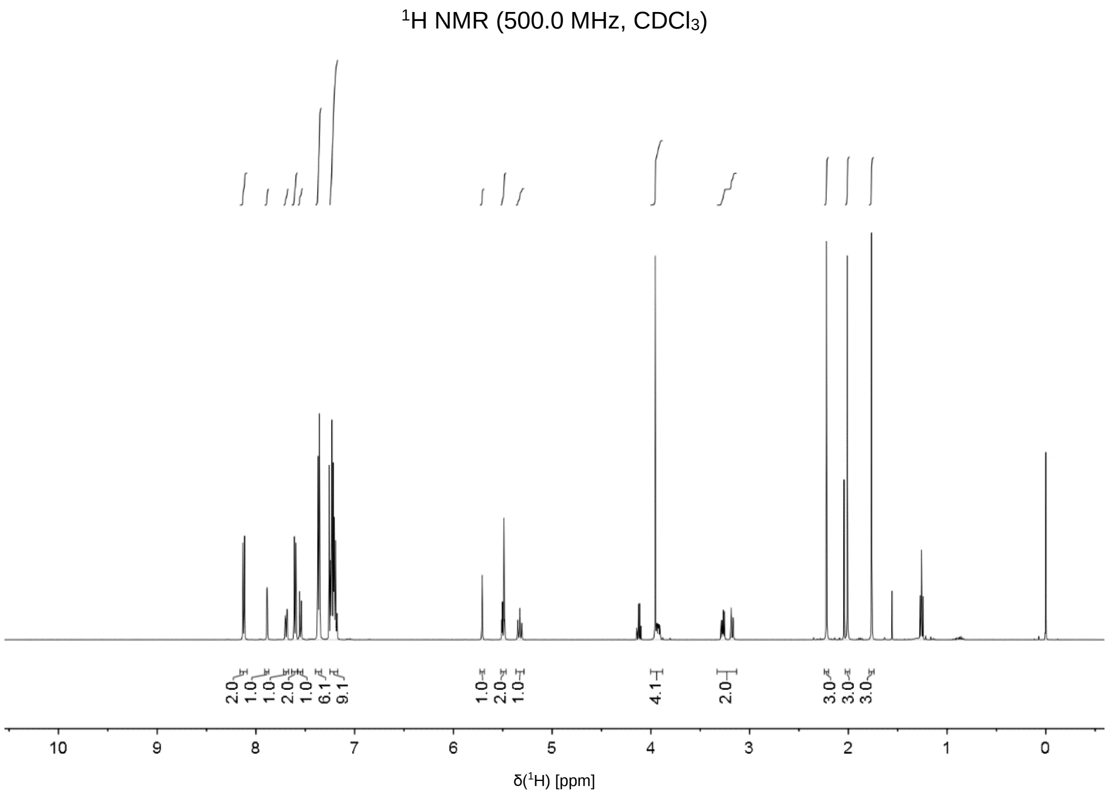

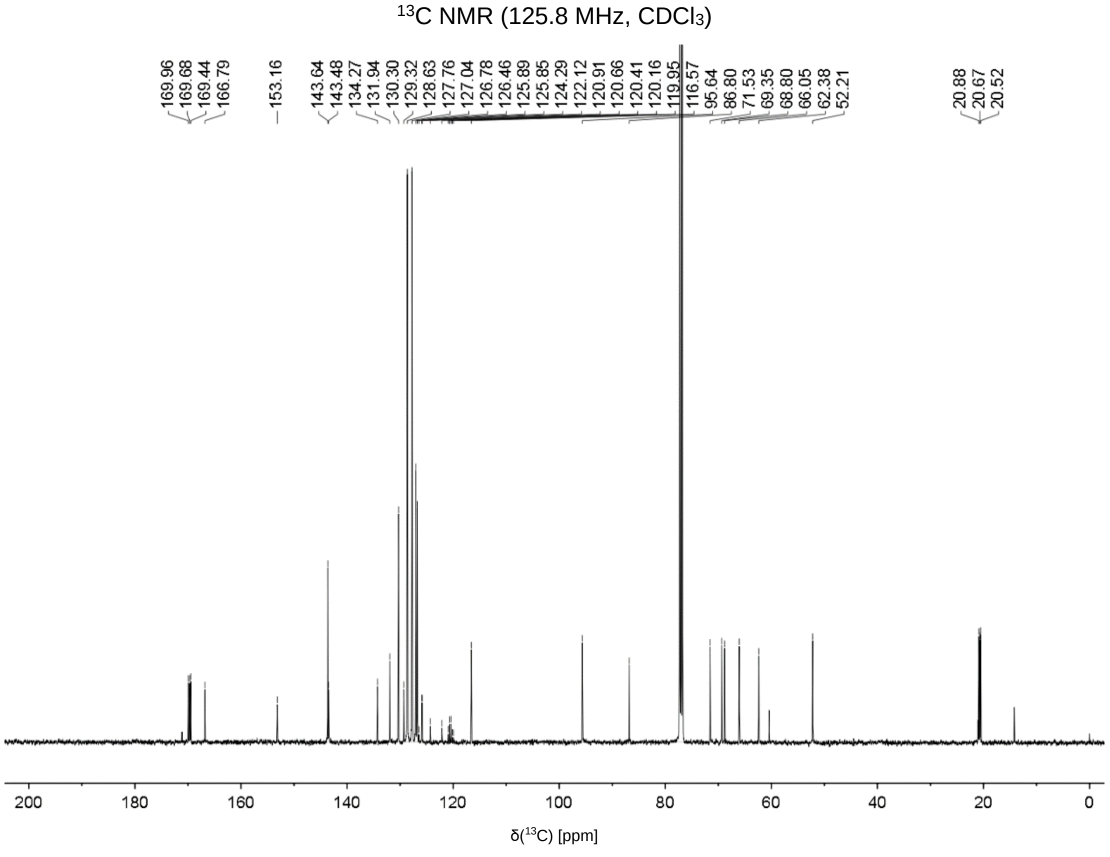

Methyl 4'-(2'',3'',4''-tri-O-acetyl-α-D-mannopyranosyloxy)-3'-trifluoromethylbiphenyl-4-carboxylate (35)

^{1}H NMR (500.0 MHz, $CDCl_3$)

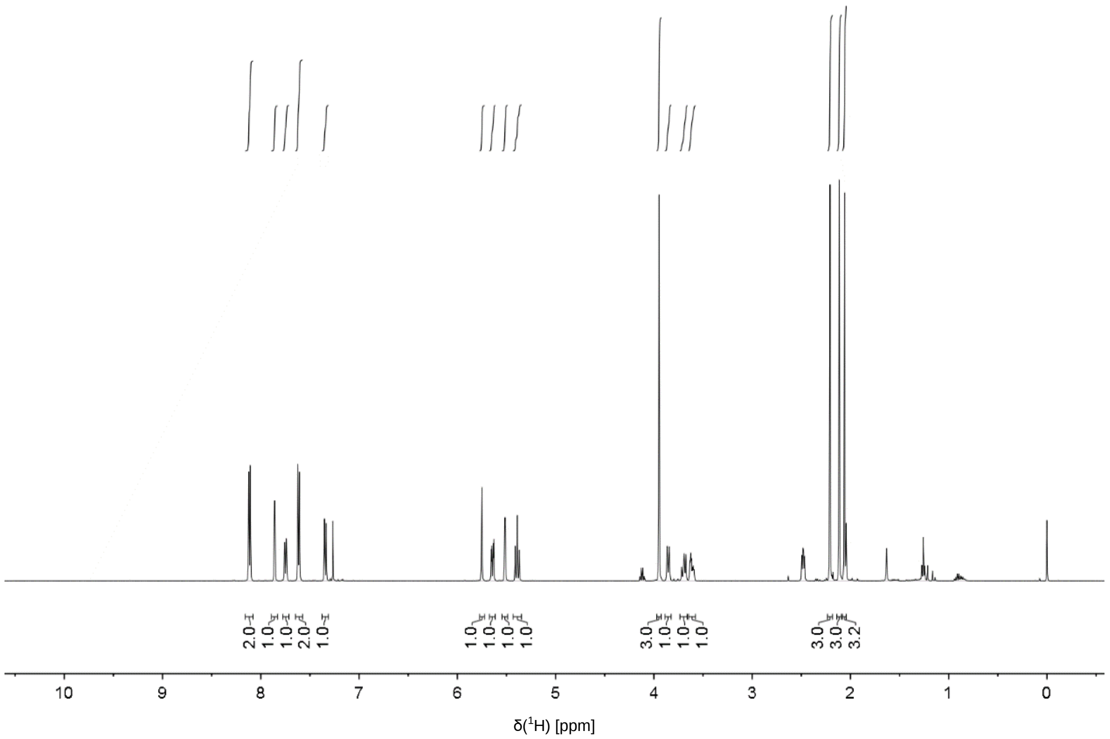

^{13}C NMR (125.8 MHz, $CDCl_3$)

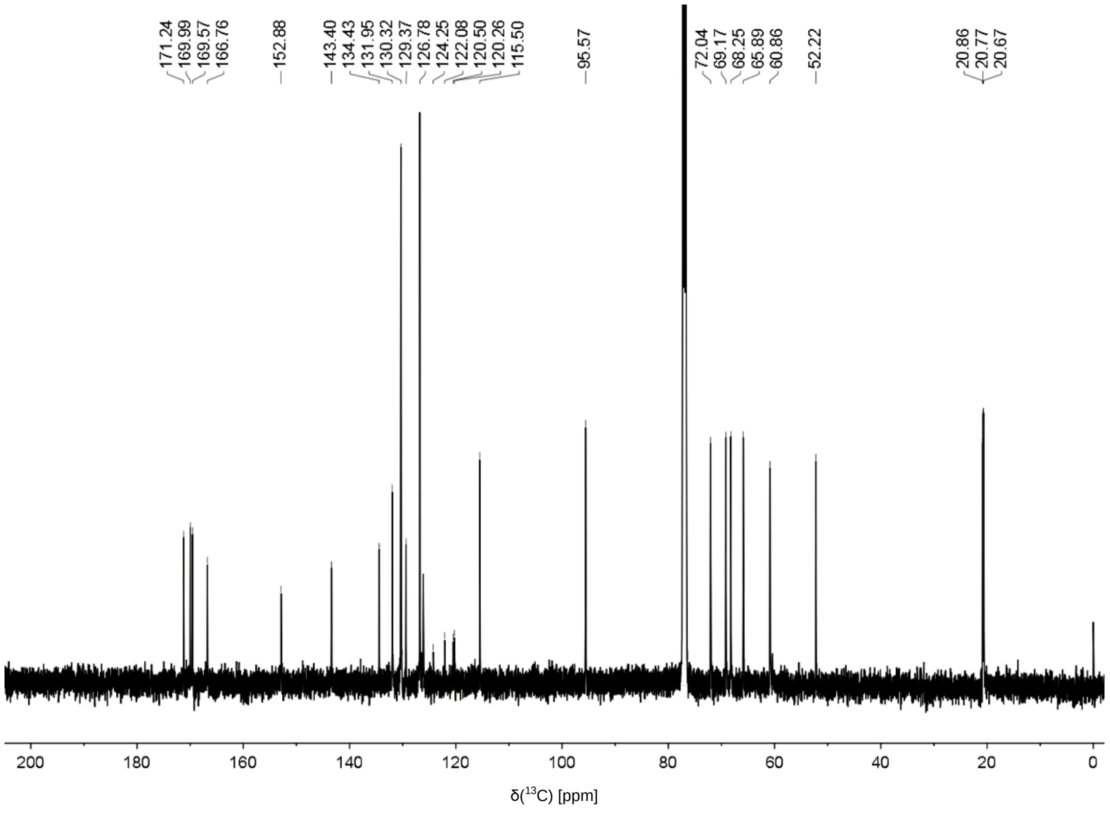

Methyl 4’-(2’’,3’’,4’’-tri-O-acetyl-6’’-O-tosyl-α-D-mannopyranosyloxy)-3’-trifluoromethylbiphenyl-4-carboxylate (36)

^{1}H NMR (500.0 MHz, $CDCl_3$)

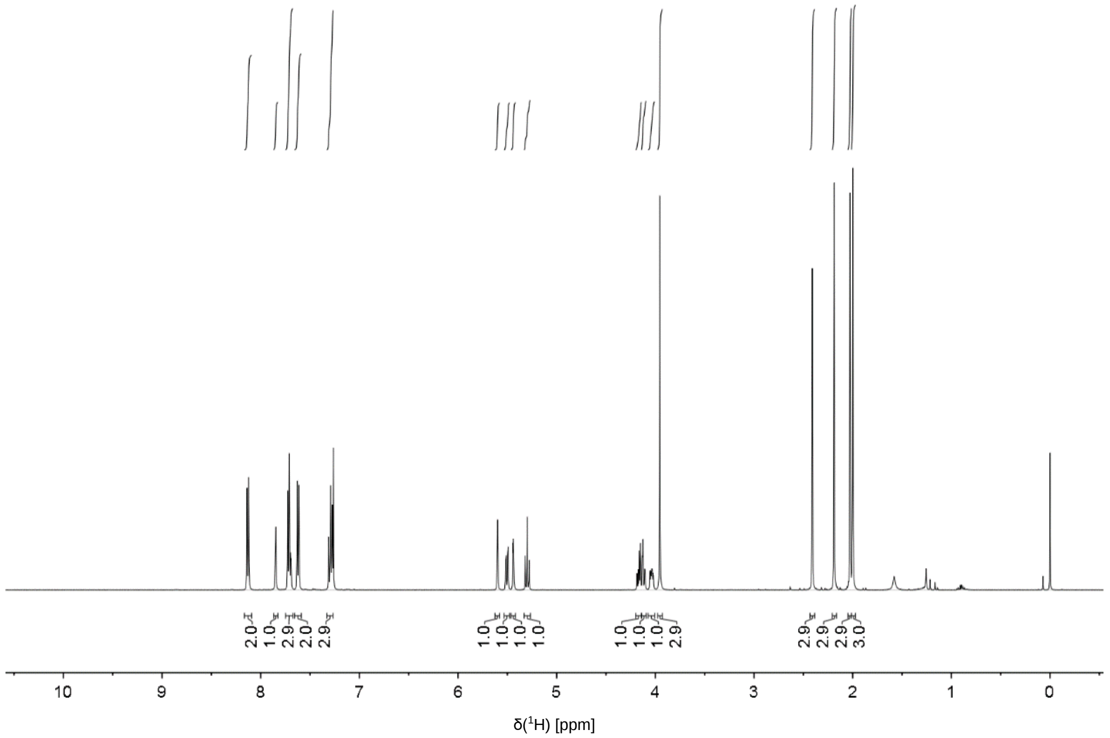

^{13}C NMR (125.8 MHz, $CDCl_3$)

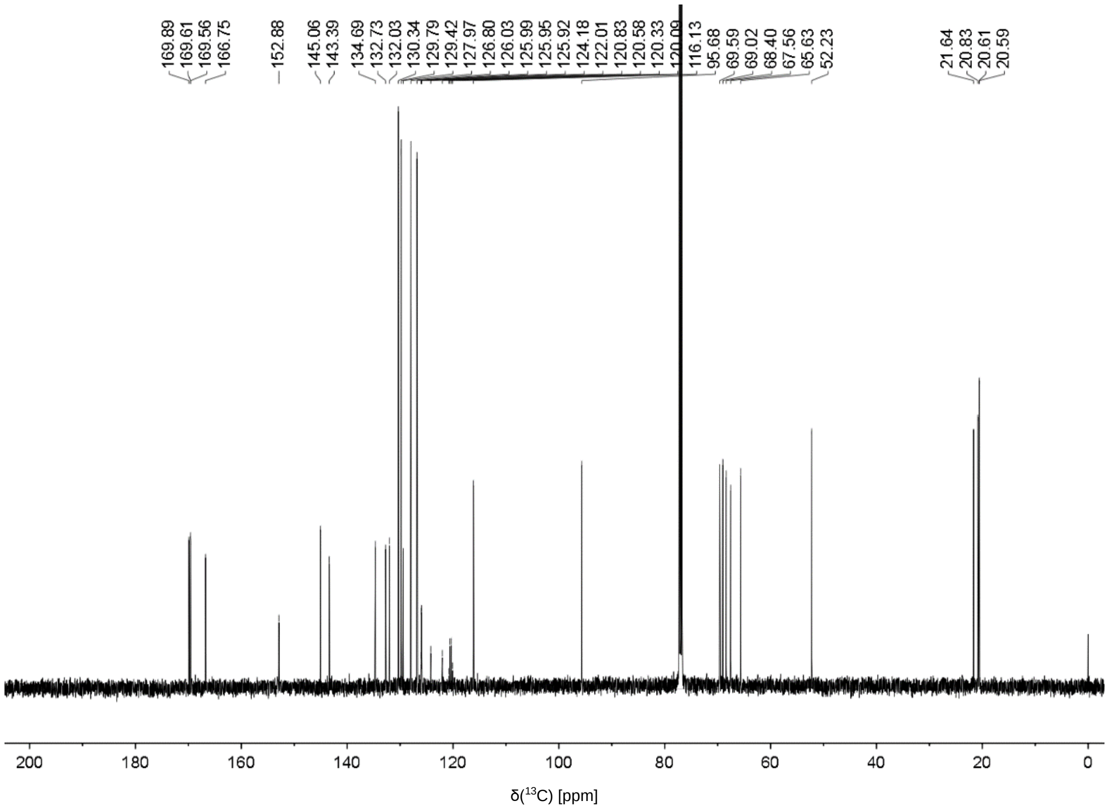

Methyl 4’-(2’’,3’’,4’’-tri-O-acetyl-6’’-deoxy-6’’-azido-α-D-mannopyranosyloxy)-3’-trifluoromethylbiphenyl-4-carboxylate (37)

^{1}H NMR (500.0 MHz, $CDCl_3$)

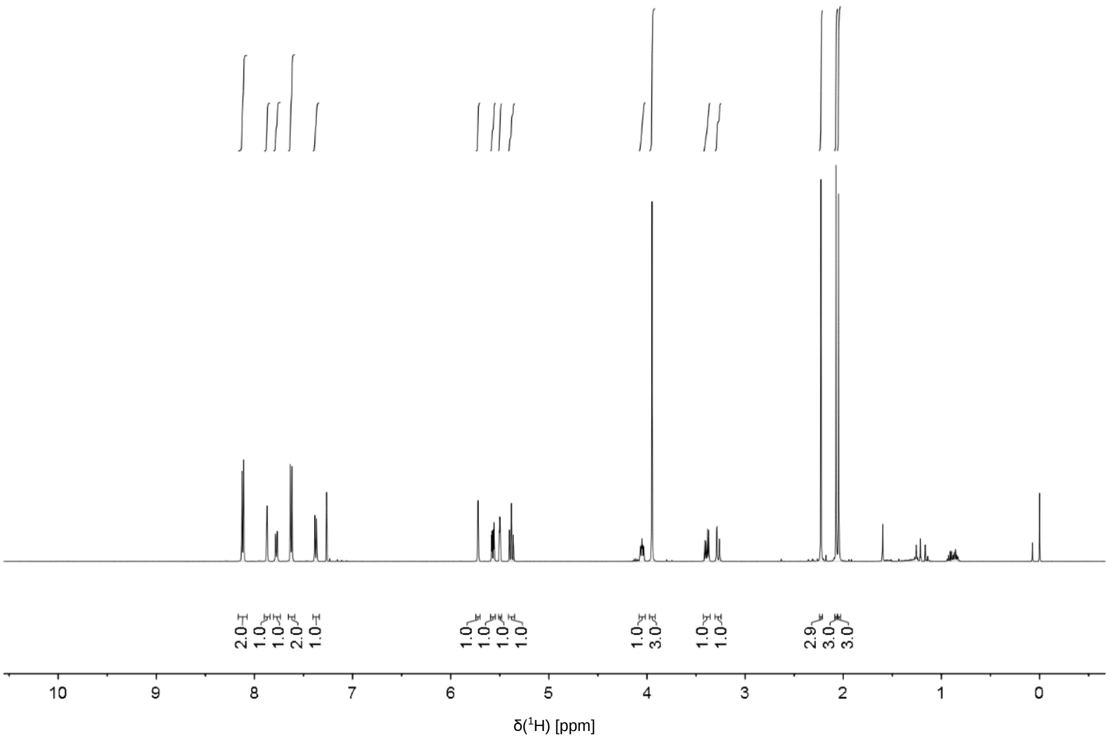

^{13}C NMR (125.8 MHz, $CDCl_3$)

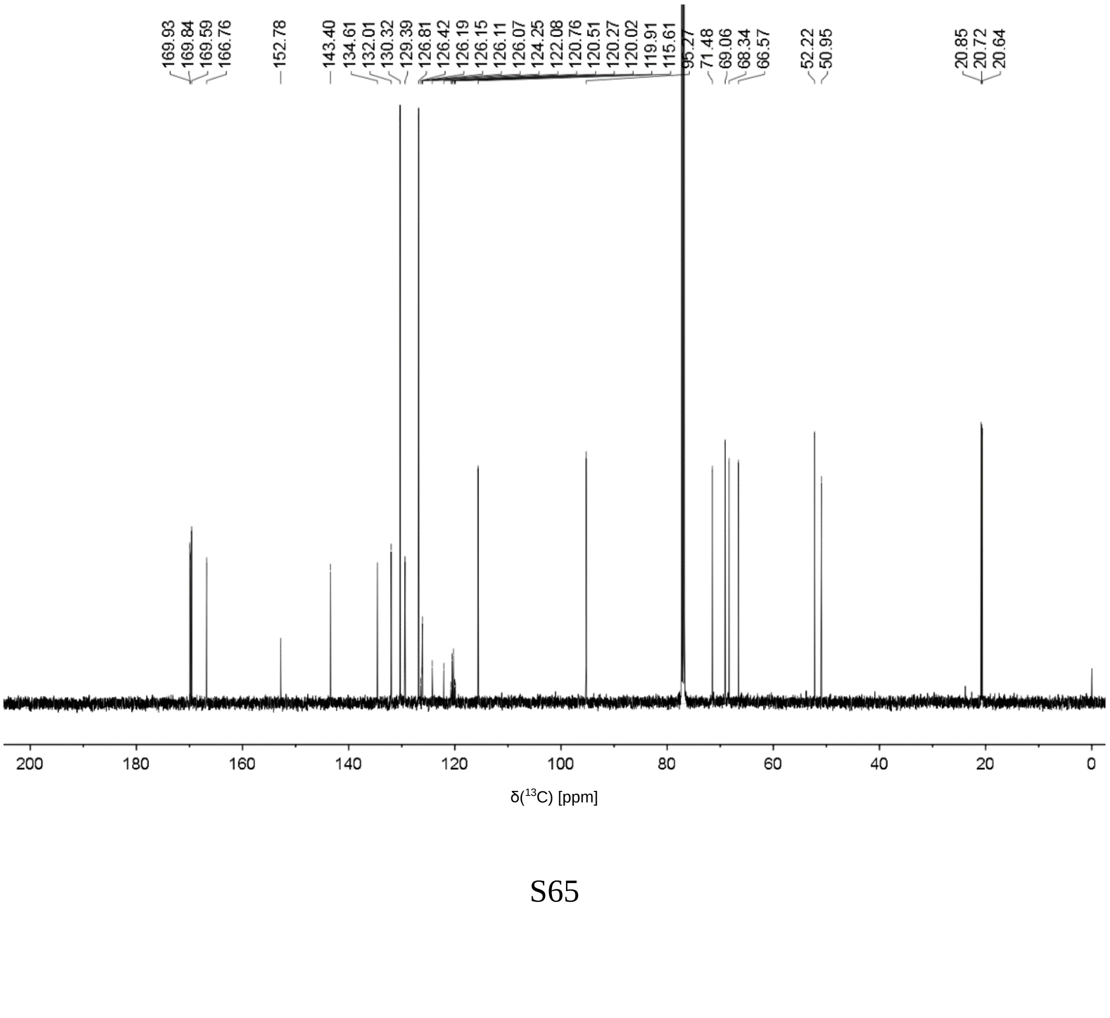

Methyl 4’-(2’’,3’’,4’’-tri-O-acetyl-6’’-deoxy-6’’-(2’’’-phthalimidoethylsulfonamido)-α-D-mannopyranosyloxy)-3’-trifluoromethylbiphenyl-4-carboxylate (40)

^{1}H NMR (500.0 MHz, MeOD)

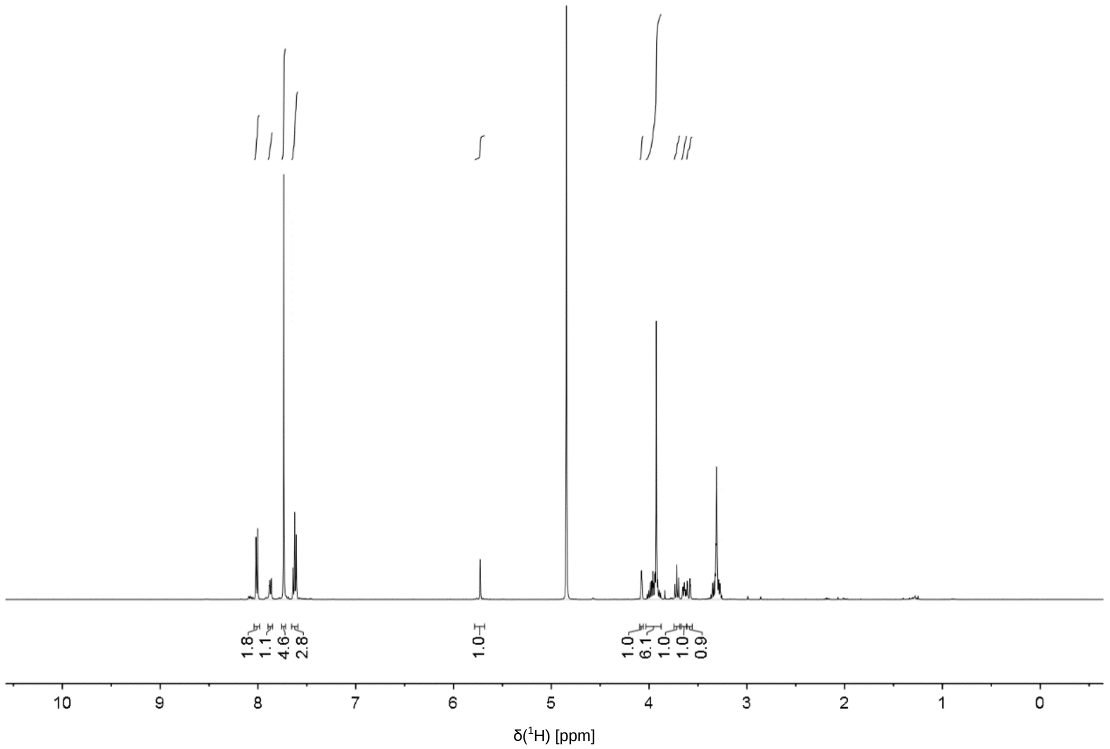

^{13}C NMR (125.8 MHz, MeOD)

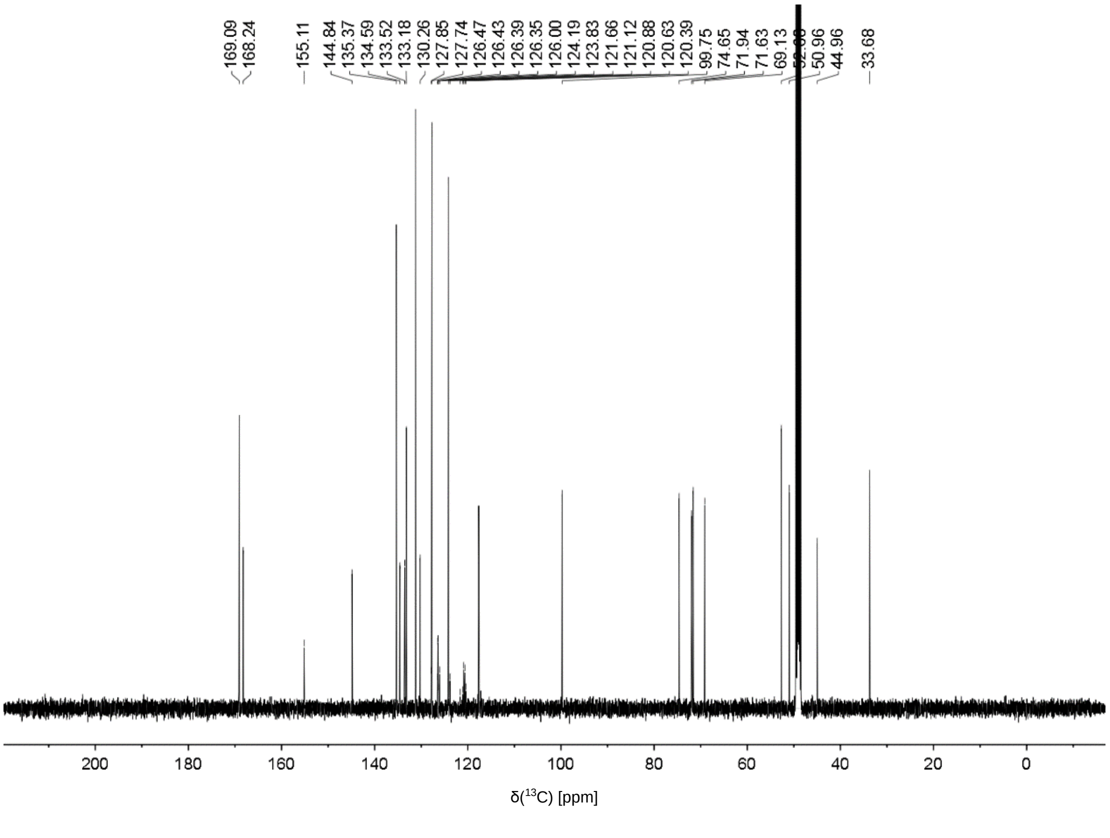

4’-(6’’-(2’’’-Aminoethylsulfonamido)-6’’-deoxy-α-D-mannopyranosyloxy)-3’-trifluoromethylbiphenyl-4-carboxylic acid (42)

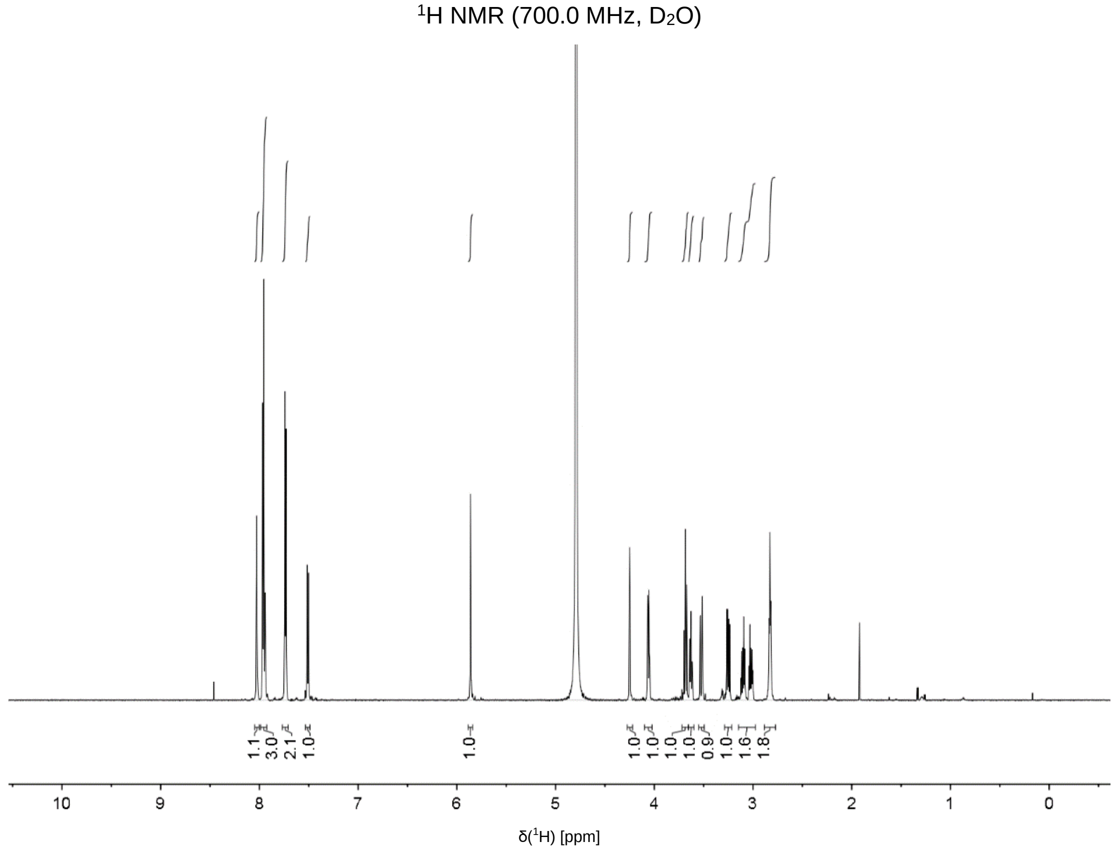

^{13}C NMR (176.0 MHz, D_2O)

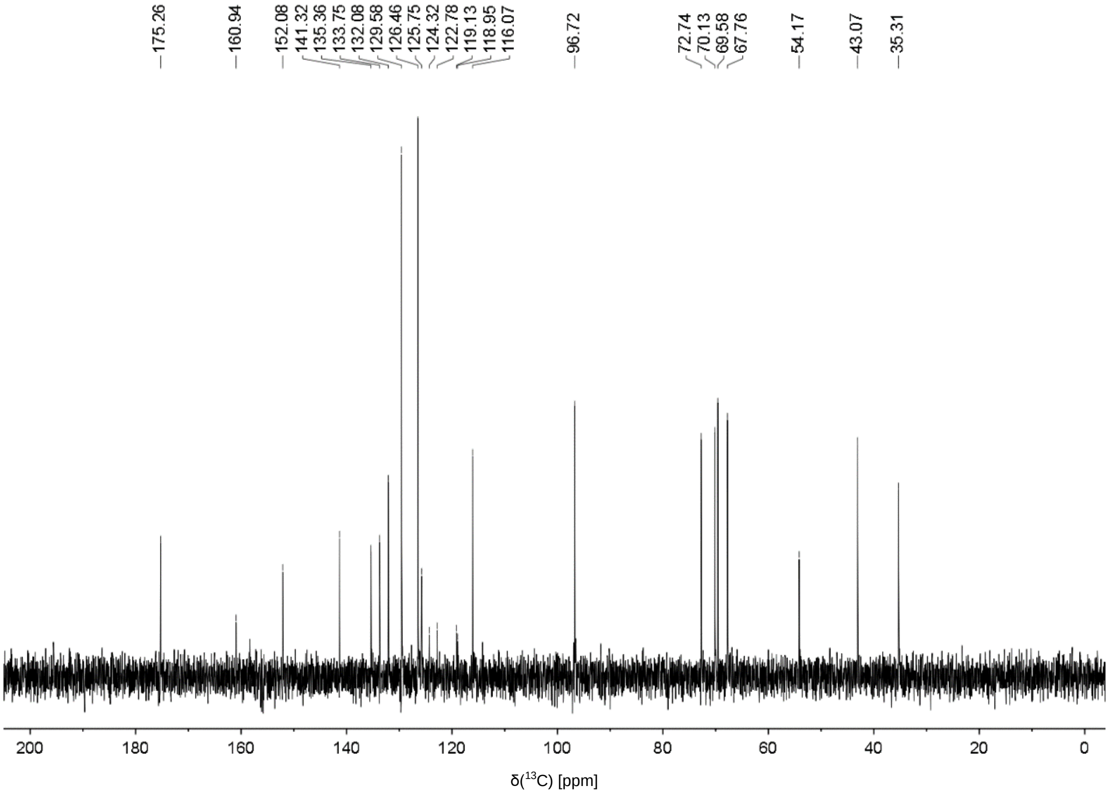

4’-(6’’-(2’’’-Acetamidoethylsulfonamido)-6’’-deoxy-α-D-mannopyranosyloxy)-3’-trifluoromethylbiphenyl-4-carboxylic acid (43)

^{1}H NMR (500.0 MHz, D_2O)

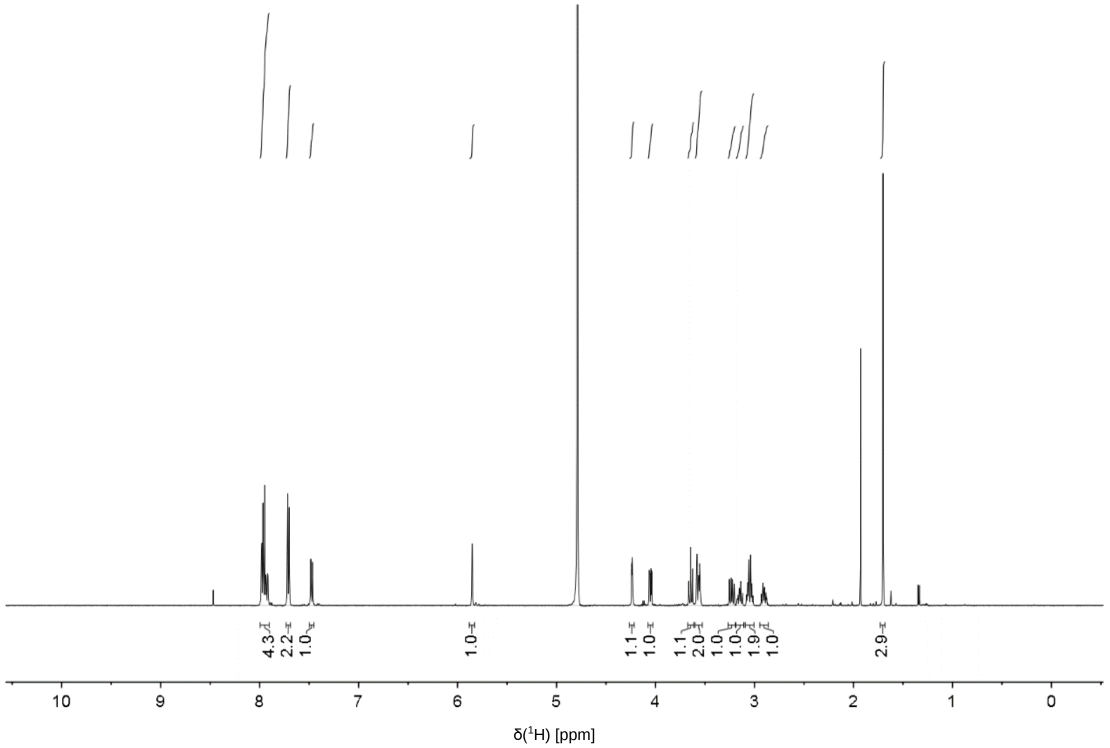

^{13}C NMR (125.8 MHz, D_2O)

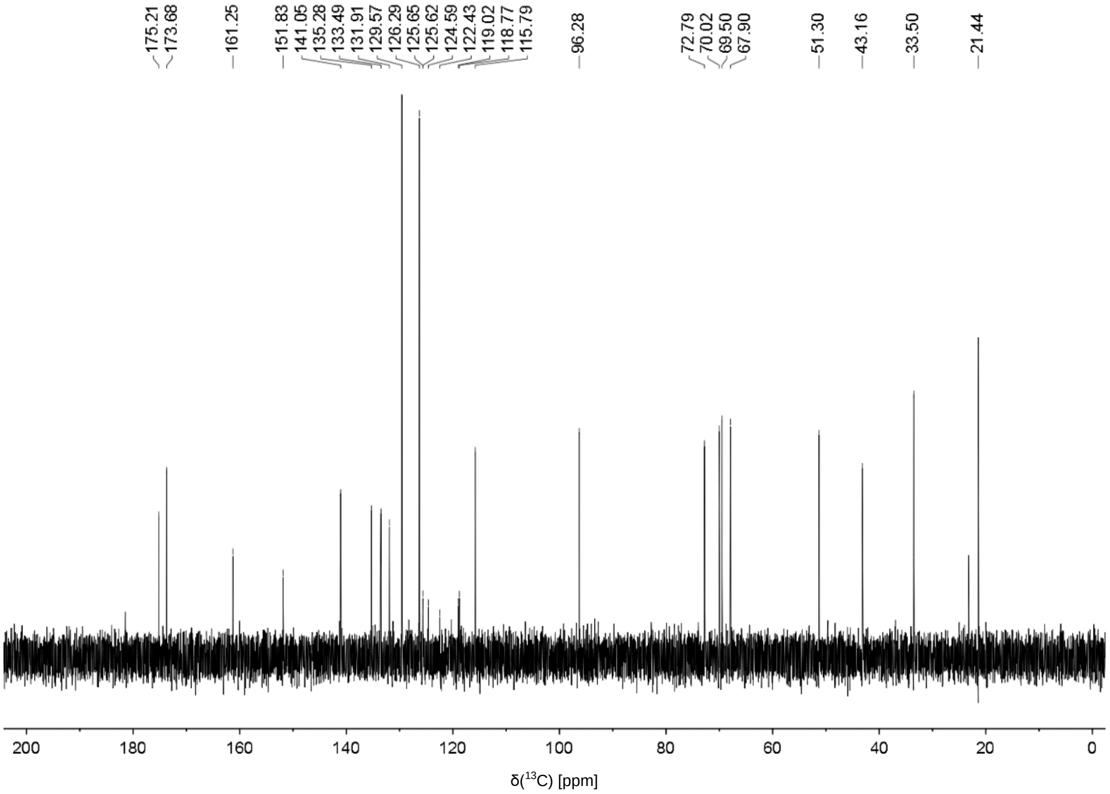

4'-(6''-Amino-6''-deoxy-α-D-mannopyranosyloxy)-3'-trifluoromethylbiphenyl-4-carboxylic acid (48)

^{1}H NMR (700.0 MHz, D_2O)

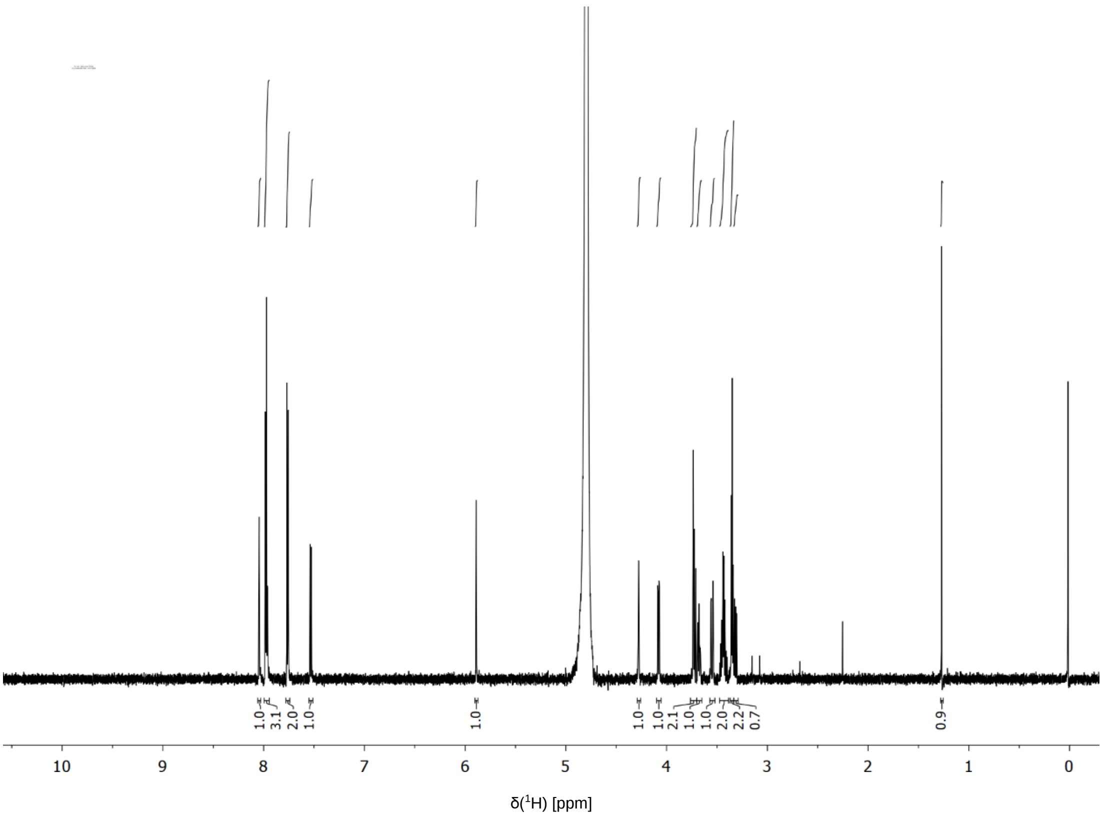

^{13}C NMR (125.8 MHz, DMSO)

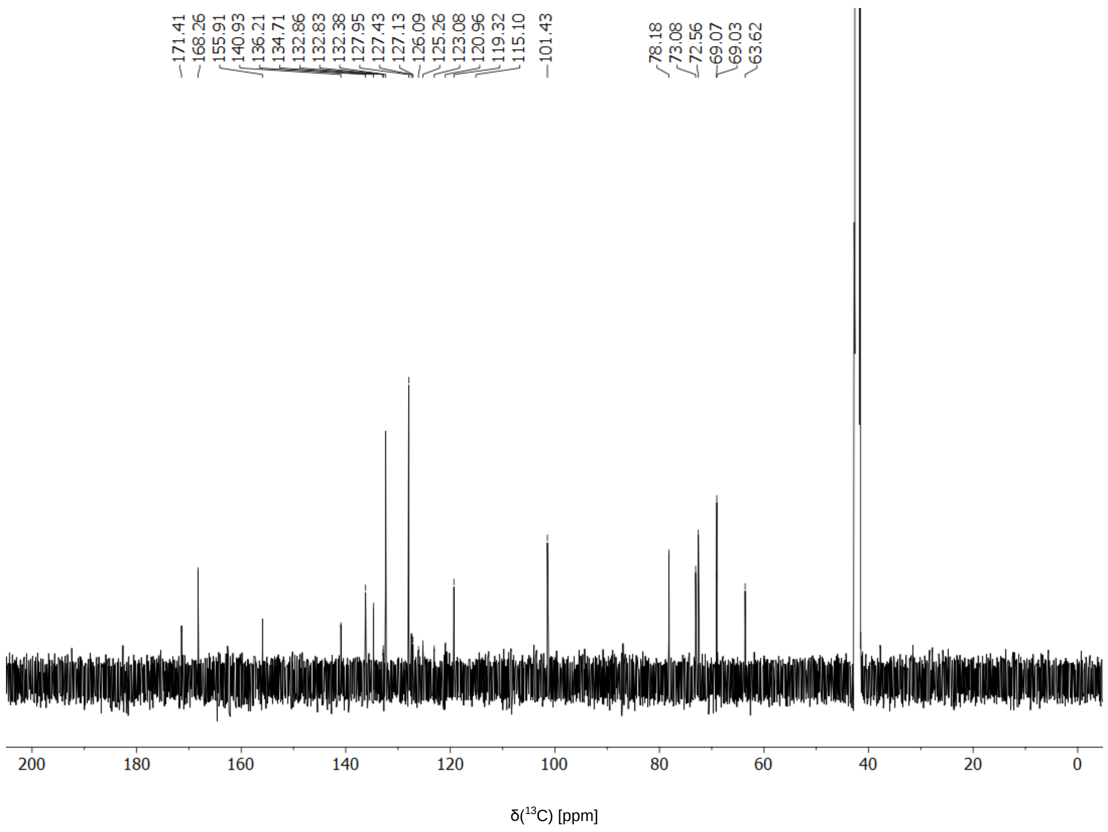

Liposome preparation

PEGylated liposomes were prepared *via* thin film hydration and subsequent pore extrusion as previously published.[26] Liposomes used in this study were comprised of DSPC (57 mol%), cholesterol (38 mol%) and DSPE-PEG$_{2kDa}$ (5 mol% total), with the latter also containing the glycolipids for targeting and Alexa 647-lipids (0.25 mol%). Notably, since efficiencies of the coupling to DSPE-PEG$_{2kDa}$-NHS differed between ligands, effective mol ratios deviated from calculated quantities. Effective mol ratios are given in the results section. Alexa 647-lipids were prepared as previously described.[4] Briefly, The DSPE-PEG components were dissolved in dimethyl sulfoxide, added to test tube and lyophilized. Next, DSPC (NOF Europe) and cholesterol (Sigma Aldrich) were dissolved in chloroform, added to the test tube and the solvents removed initially using an N_2 gas stream and subsequently *in vacuo*. The residue was dissolved in PBS (pH = 7.4) and the mixture was vortexed and sonicated repeatedly to obtain a homogeneous suspension. The resulting unilamellar liposomes further treated using a pore extruder (Avanti Polar Lipids) with polycarbonate membranes of 800, 400, 200 and finally 100 nm pore size (Avanti Polar Lipids). Liposomes were stored at 4° C. Characterization by dynamic light scattering (DLS, Malvern Zetasizer Nano ZS) and electrophoresis experiments was conducted to characterize liposome dispersity, size and Z potential (**Scheme S4**).

Cell culture

If not stated otherwise, all media and supplements for cell culture experiments were purchased from Thermo Fisher Scientific. Raji, THP-1 and U937 cell lines (ATCC) were grown in complete growth medium containing RPMI1640 medium, 10% FCS, 100 U mL^{-1} Penicillin-Streptomycin and GlutaMax at 37°C and 5% CO_2. Cells were monitored with a light microscope (IT40 5PH, VWR) and subcultured every 2-3 days to maintain cell densities ranging from of 0.5 - 3 x 10^6 cells per mL.

CLR-expressing cell lines were generated as described before.[4,27] Briefly, DC-SIGN, Langerin, murine Langerin and murine Dectin-1 cDNAs (Sinobiologicals) were cloned into a lentiviral BIC-PGK-Zeo-T2a-mAmetrine:EF1A construct by Gibson assembly (NEB) according to the manufacturer's protocol. HEK293 cells were transfected with the lentiviral vector together with third-generation packaging vectors and viral particles were then used for transduction of Raji cells. In analogy, DC-SIGN and Langerin-expressing THP-1 and U937 cell lines were generated by lentiviral transduction.

Liposome binding assay – flow cytometry

Liposome binding to CLR-expressing cells was assayed as previously described.[27] Briefly, 0.05 x 10^6 cells were plated in transparent conical-bottom 96 well microtiter plates (Nunc) in a volume of 100 µL medium. Plates were centrifuged at 500 x g for 3 minutes, supernatant was aspirated, and cells were resuspended in 100 µL medium, containing 16 µM liposomes. In control experiments, cells were incubated with medium containing 10 mM EDTA or 50 µg mL^{-1} mannan for 15 min at 4 °C, prior to liposome application. After 1 h incubation at 4 °C in the dark, cells were centrifuged at 500 x g for 3 min and the supernatant was discarded. Cells were resuspended in 200 µL ice-cold medium and analyzed by detecting the co-formulated Alexa 647 dye via flow cytometry with a 654 nm laser and a 670/14 nm filter (Attune Nxt, life technologies). Flow cytometry data was processed using FlowJo (BD Bioscience). Mean fluorescence intensity (MFI) was further analyzed and plotted using GraphPad Prism (GraphPad Software).

To monitor allosteric activation of DC-SIGN by glycomimetic **48**, the described protocol was adjusted as follows. DC-SIGN$^+$ Raji cells were plated in transparent conical-bottom 96 well microtiter plates (Nunc) in a volume of 100 µL medium. Plates were centrifuged at 500 x g for 3 minutes, supernatant was aspirated, and cell were resuspended in 25 µL medium supplemented with varying concentrations of monovalent carbohydrates or 0.5 mM **48**. Homomultivalent liposomes carrying either natural carbohydrates or **48** were added at a final concentration of 16 µM. Liposome binding was subsequently quantified by measuring Alexa 647 fluorescence in flow cytometry experiments. Statistical significance by means of a student's t-test was assessed based on averaged normalized MFIs from four biological replicates each conducted as technical duplicates. A p-value < 0.05 was set as significance cut-off.

Receptor expression and purification

General remarks. Codon-optimized genes for the bacterial expression of DC-SIGN ECD and the His-tagged DC-SIGN CRD wild-type as well as the M270F mutant were purchased from GenScript. Unless stated otherwise, all growth media, chemicals and enzymes used for receptor expression and purification were purchased from Sigma Aldrich or Carl Roth.

Langerin extracellular domain (ECD). Expression and purification were conducted as previously published.[28] Briefly, the trimeric Langerin extracellular domain (ECD) was expressed insolubly in *E. coli* BL21* (DE3) (Invitrogen). Following enzymatic cell lysis, inclusion bodies (IBs) were harvested and subsequently solubilized. The sample was centrifuged and the Langerin ECD was refolded overnight via rapid dilution. Next, the sample was dialyzed overnight, centrifuged and purified via mannan-agarose affinity chromatography (Sigma Aldrich). For ^{19}F R_2-filtered NMR experiments, the buffer was exchanged to 25 mM Tris with 150 mM NaCl and 5 mM $CaCl_2$ at pH 7.8 using 7 kDa size-exclusion desalting columns (Thermo Scientific). For STD NMR experiments, Langerin ECD samples were dialyzed five times for at least 8 h against H_2O. Subsequently, the H_2O was removed via lyophilization and the residue was stored at -80° C. Prior to STD NMR experiments, the Langerin ECD was dissolved in 25 mM Tris-d_{11} (Eurisotope) with 100% D_2O, 150 mM NaCl and 5 mM $CaCl_2$ at pH 7. The concentration of Langerin ECD was determined via UV spectroscopy ($A_{280,\,0.1\%}$ = 2.45). Purity and monodispersity of Langerin ECD samples were analyzed via SDS PAGE and DLS.

Langerin carbohydrate recognition domain (CRD). Expression and purification were conducted as previously published.[28] Briefly, the monomeric ^{15}N-labeled Langerin carbohydrate recognition domains (CRDs) were expressed insolubly in *E. coli* BL21* (DE3) (Invitrogen). Following enzymatic cell lysis, IBs were harvested and subsequently solubilized. The sample was centrifuged and the Langerin CRD was refolded overnight via rapid dilution. Next, the sample was dialyzed overnight, centrifuged and purified via StrepTactin affinity chromatography (Iba). After an additional dialysis step overnight, the sample was centrifuged and the buffer was exchanged to 25 mM HEPES with 150 mM NaCl at pH 7.0 using 7 kDa size-exclusion desalting columns (Thermo Fisher Scientific) for ^{15}N HSQC NMR experiments. The concentration of Langerin was determined via UV spectroscopy ($A_{280,\,0.1\%}$ = 3.19). Sample purity and monodispersity were analyzed via SDS PAGE and DLS.

DC-SIGN extracellular domain (ECD). DC-SIGN ECD was expressed and purified from inclusion bodies (IBs) as described before, with minor changes.[28] Briefly, *E.coli* BL21(DE3) carrying the DC-SIGN ECD-encoding pET30b plasmid were grown in Luria-Bertani (LB) medium supplemented with 35 mg L^{-1} kanamycin at 37°C. Receptor expression was induced with 1 mM isopropyl-β-D-thiogalactopyranoside (IPTG) at

OD_{600} ~ 0.9 for 4 h at 37°C. Bacteria were harvested by centrifugation (4.000 x g, 30 min, 4°C), resuspended in lysis buffer (50 mM Tris-HCl, 10 mM $MgCl_2$, 0.1% Triton X-100, 4 mg lysozyme, 500 U DNAseI per g cell pellet, pH 7.8) and lysed by sonication on ice. IBs were harvested by centrifugation (15.000 x g, 90 min, 4°C) and washed three times with lysis buffer and H_2O to remove soluble proteins. Washed IBs were solubilized in 20 mL denaturation buffer (6 M guanidine hydrochloride, 100 mM Tris-HCl, 1 mM DTT, pH 8.0) for 1 h at 37°C. After centrifugation (15.000 x g, 90 min, 4°C), solubilized IBs where rapidly diluted into 180 mL refolding buffer (50 mM Tris-HCl, 0.8 M L-arginine, 20 mM NaCl, 2.5 mM reduced glutathione, 0.5 mM oxidized glutathione, pH 7.8) and stirred overnight at 4°C. The protein solution was then dialyzed overnight at 4°C against 5 L of mannan-chromatography binding buffer (MCBB) (25 mM Tris-HCl, 150 mM NaCl, 25 mM $CaCl_2$, pH 7.8). After another dialysis step against 5 L MCBB, precipitated protein was removed by centrifugation (15.000 x g, 15 min, 4°C) and the DC-SIGN ECD was purified using a mannan agarose affinity chromatography as described previously.[29] Purified receptor was dialyzed against 5 L HBS (20 mM HEPES, 150 mM NaCl, pH 7.4) supplemented with 2 mM $CaCl_2$ overnight at 4°C. DC-SIGN ECD samples were concentrated using centrifugal filtration and concentration was quantified *via* UV spectroscopy (with A_{280}, 0.1% = 1.812). Sample purity was analyzed via SDS PAGE. The protein solution was aliquoted, snap frozen in liquid N_2 and stored at -80°C until further usage.

DC-SIGN carbohydrate recognition domain (CRD). His-tagged DC-SIGN CRD wildtype and the M270F mutant-encoding pET28a plasmids were expressed and purified from inclusion bodies as described previously, with minor changes.[28] Briefly, transformed *E.coli* BL21(DE3) were grown in M9 minimal medium containing ^{15}N-labeled NH_4Cl (Silantes), supplemented with 35 mg L^{-1} ampicillin at 37°C. Protein expression was induced with 1 mM IPTG at OD_{600} ~ 0.9 for 4 h at 37°C. Bacteria were harvested by centrifugation (4.000 x g, 30 min, 4°C), resuspended in lysis buffer and lysed by sonication on ice. IBs were harvested by centrifugation (15.000 x g, 90 min, 4°C) and washed thrice with lysis buffer and ultrapure water to remove soluble proteins. Washed IBs were solubilized in 20 mL denaturation buffer for 1 h at 37°C. After centrifugation (15.000 x g, 90 min, 4°C), solubilized IBs where rapidly diluted into 180 mL refolding buffer and stirred overnight at 4°C. The protein solution was then dialyzed overnight at 4°C against 5 L TBS (100 mM Tris-HCl,150 mM NaCl, pH 7.8). After another dialysis step against 5 L TBS, precipitated protein was removed by centrifugation (15.000 x g, 15 min, 4°C) and the DC-SIGN CRD was purified using Ni^{2+}-NTA affinity chromatography according to manufacturer's instructions (Qiagen). Purified receptor was dialyzed against 5 L MES low salt buffer (20 mM MES, 40 mM NaCl, pH 6.0) supplemented with 5 mM $CaCl_2$ overnight at 4°C. DC-SIGN CRD samples were concentrated using centrifugal filtration and concentration was quantified *via* UV spectroscopy (with A_{280}, 0.1% = 2.966). Sample purity was analyzed

via SDS PAGE. The protein solution was aliquoted, snap frozen in liquid N_2 and stored at -80°C until further usage.

NMR binding experiments – Langerin

^{19}F R_2-filtered NMR

General remarks. ^{19}F R_2-filtered NMR experiments were conducted on a PremiumCompact 600 MHz spectrometer (Agilent). Spectra were processed in MestreNova (Mestrelab Research) and data analysis was performed with OriginPro (OriginLab). Experiments with the Langerin ECD were performed at a receptor concentration of 50 µM in 25 mM Tris with 10% D_2O, 150 mM NaCl and 5 mM $CaCl_2$ at pH 7.8 and 25°C. Experiments with the DC-SIGN CRD were performed at a receptor concentration of 50 µM in 25 mM HEPES with 10% D_2O, 150 mM NaCl and 5 mM $CaCl_2$ at pH 7.0 and 25°C. TFA served as an internal reference at a concentration of 50 µM. Apparent transverse relaxation rates $R_{2,obs}$ for the reporter ligand were determined using the CPMG pulse sequence as previously published.[6,30,31]

Screening of mannoside library. Estimated affinities $K_{I,est}$ were determined in competitive binding experiments at 0.1 mM of reporter ligand **49** at 0.1 mM using the Langerin ECD at a single competitor concentration as previously described.[6] Here, receptor concentrations $[P]_T$ values were directly calculated from $R_{2,max}$, the data point at 0.1 mM **49** in absence of competitor. Subsequently, K_I values were estimated in a one parameter fit. The screening was conducted in presence of 0% to 10% DMSO at mannoside concentrations between 0.1 and 10 mM. Overall, 27 glycomimetics were screened and their solubility was monitored either by visually or in selected cases via ^{1}H NMR experiments using TSP-d_6 as an internal reference at 0.1 mM.

K_I determination. K_I values were determined as previously published for Langerin.[6] Briefly, titration experiments were conducted at a concentration of 0.1 mM of reporter ligand **49** at five competitor concentrations $[I]_T$ (**Table S5**).

STD NMR

General remarks. STD NMR experiments were conducted on a PremiumCompact 600 MHz spectrometer (Agilent).[32] Spectra were processed in MestreNova (MestreLab Research) and data analysis was performed with OriginPro (OriginLab). Experiments with the Langerin ECD were conducted at a receptor concentration of 50 µM in 25 mM Tris-d_{11} (Eurisotope) with 100% D_2O, 150 mM NaCl and 5 mM $CaCl_2$ at pH 7.8 and 25°C in presence of 10% DMSO. Experiments were repeated in absence of receptor to exclude STD effects due to direct saturation of ligands. Residual H_2O or TSP-d_6 at 0.1 mM served as an internal reference. Spectra were recorded in 5 mm sample tubes (Norrell) at sample volumes of 500 µl. Saturation was implemented via a train of 50 ms Gauss pulses at varying saturation times t_{sat}. The on-resonance irradiation frequency ν_{sat} was set to 0.0 ppm and the off-resonance irradiation frequency ν_{ref} was set to 80.0

ppm. The acquisition time t_{acq} was set to 2.0 s and the DPFGSE pulse sequence was utilized for solvent suppression.[33] Receptor resonances were suppressed using a $T_{1,rho}$ filter at a relaxation time τ of 35 ms.

Epitope mapping. The binding epitope for **43** was determined at a concentration of 500 µM. For each spectrum 512 scans were recorded. The relaxation delay d_1 was set to 6 s and spectra were recorded at 5 different saturation times t_{sat} varying from 0.25 to 6.00 s. **Equation 1** served to derive the STD effect STD for each analyzed resonance from the corresponding on- and off-resonance spectra.[34] I_0 represents the integral of a resonance in the off-resonance spectrum and I_{sat} represents the integral of a resonance in the on-resonance spectrum.

$$STD = \frac{I_0 - I_{sat}}{I_0}$$

Equation 1

The apparent saturation rate k_{sat} and the maximal STD effect STD_{max} were derived from **Equation 2** in a two-parameter fit.[35] Standard errors were derived directly from the fitting procedures. These parameters were used to calculate the initial slope of the STD build-up curves STD'_0 via **Equation 3**. STD'_0 values were normalized and mapped on the corresponding ligand structure. Only resonances for which at least part of a multiplet was isolated were considered for the epitope mapping.

$$STD = STD_{max}(1 - e^{k_{sat}t_{sat}})$$

Equation 2

$$STD'_0 = STD_{max}k_{sat}$$

Equation 3

^{15}N HSQC NMR

General remarks. ^{15}N HSQC NMR experiments were conducted on an Ascend 700 MHz spectrometer (Bruker).[36] Spectra were processed in NMRPipe.[37] Data analysis was performed using CCPN Analysis, MatLab (MathWorks) and OriginPro.[38] Experiments with the Langerin CRD were performed at a receptor concentration of 100 µM in 25 mM HEPES with 10% D_2O, 150 mM NaCl and 5 mM $CaCl_2$ at pH 7.8 and 25° C. DSS-d_6 served as an internal reference at a concentration of 100 µM. Spectra were referenced via the internal spectrometer reference. Spectra were acquired with 128 increments and 32 scans per increments for 150 µl samples in 3 mm sample tubes (Norrell). The relaxation delay d_1 was set to 1.4 s and the acquisition time t_{acq} was set to 100 ms. The W5 Watergate pulse sequence was used for solvent suppression.[39] The used resonance assignment for the Langerin CRD has been published previously.[7] Titration experiments with **9**, **25**, **43** and **46** were conducted in presence

of 10% DMSO. Here, assignments were transferred from a reference spectrum in absence of DMSO to the nearest neighbor in the reference spectrum in presence of DMSO. In case this approach was ambiguous, the corresponding resonances were flagged during data processing and analysis.

K_D determination. K_D values were determined in titration experiments at six ligand concentrations $[L]_T$. Samples were prepared via serial dilution. Chemical shift perturbations CSPs for Langerin CRD resonances in the fast or fast-to-intermediate exchange regime observed upon titration with ligand were calculated as previously described via **Equation 4**.[40]

$$CSP = \sqrt{\frac{\delta(1H) + \left(0.15\delta(15N)\right)^2}{2}}$$

Equation 4

A standard deviation σ of 0.02 ppm was previously determined for the measurement of chemical shifts in ^{15}N HSQC NMR experiments with the Langerin CRD.[7] Accordingly, only assigned resonances that displayed CSP values higher than a threshold of σ at the highest ligand concentration were selected for the determination of K_D values via **Equation 5** in a global two-parameter fit.[38] Standard errors were derived directly from the fitting procedures. Additionally, resonances that displayed line broadening $\Delta\nu_{0.5}$ larger than 10 Hz upon titration in either the ^{1}H or the ^{15}N dimension were not considered for the determination of K_D values. CSP_{max} represents the CSP value observed upon saturation of the CBS.

$$CSP = CSP_{max} p_b$$

with

$$p_b = \frac{[P]_T + [L]_T + K_D - \sqrt{([P]_T + [L]_T + K_D)^2 - 4[P]_T[L]_T}}{2[P]_T}$$

Equation 5

Binding mode analysis. Based on the resonance assignment, CSP values observed at maximal ligand concentrations $[L]_T$ were mapped on the X-ray structure of the Langerin CRD (PDB code: 3P5F) using Matlab's Bioinformatics Toolbox (MathWorks) via substitution of the B-factor values.[5] The CSP patterns obtained were visualized in MOE using Chain B of the Langerin CRD in complex with **Man**. Model quality was maintained using MOE's Structure Preparation followed by the simulation of protonation states and the hydrogen bond network of the complex with MOE's Protonate 3D. Receptor surfaces were visualized in Connolly representation.[41]

NMR binding experiments – DC-SIGN

General Remarks

All NMR measurements were performed on a 700 MHz Bruker AVANCEIII HD spectrometer (Bruker) equipped with a 5 mm TCI CryoProbe at 25°C. ^{19}F NMR and ^{15}N HSQC NMR experiments were conducted in 3 mm NMR tubes (Norell) at sample volumes of 0.15 mL. ^{1}H STD NMR experiments were conducted in 5 mm NMR tubes (Norell) at sample volumes of 0.5 mL. Unless stated otherwise, chemicals used for buffers were purchased from Sigma Aldrich or Carl Roth. Data processing and analysis was conducted in MestreNova (MestreLab Research) for 1D spectra and in NMRPipe and CCPNMR for 2D spectra.[37,38] Further analysis, plotting and curve fitting was performed in OriginPro (OriginLab). Unless stated otherwise, all error values were directly derived from the fitting procedures.

^{19}F R_2-filtered NMR

For ^{19}F R_2-filtered NMR experiments, samples contained 12.5 µM (K_D determination) or 25 µM (K_I determination) DC-SIGN ECD in HBS at pH 7.4 supplemented with 2 mM $CaCl_2$ in presence of 10% D_2O and varying concentrations of ligand. TFA at a concentration of 0.1 mM served as an internal reference. Apparent transverse relaxation rates $R_{2,obs}$ were obtained using the CPMG sequence with a relaxation delay d_1 of 2.0s, acquisition time t_{acq} of 0.8 s and a CPMG frequency v_{CPMG} of 500 Hz.[30,42] For samples containing more than 0.1 mM ligand, 128 scans were recorded. At lower concentrations, 512 scans were recorded to ensure sufficient signal to noise ratios. $R_{2,obs}$ values were obtained by fitting **Equation 6** to integrals I of the ^{19}F resonance of the reporter ligands **48** or **49** at different relaxation times T with I_0 as integral at T = 0 s.

$$I = I_0 e^{-R_{2,obs}T}$$

Equation 6

Determined $R_{2,obs}$ values at different ligand concentrations $[L]_T$ were used to derive the relaxation rate $R_{2,b}$ of the ligand in its bound state and K_D values at a defined protein concentration $[P]_T$ in a two-parameter fit from **Equation 7** with p_b representing the fraction of bound protein. Three independent titration experiments were conducted. The relaxation rate $R_{2,f}$ of the ligand in the unbound state was determined in three independent measurements at a ligand concentration of 1 mM without protein. Relaxation dispersion experiments with 1 mM **49** in the presence of DC-SIGN ECD served to estimate the chemical exchange contribution $R_{2,ex}$.

$$R_{2,obs} = R_{2,f} + (R_{2,b} - R_{2,f})p_b$$

with

$$p_b = \frac{[P]_T + [L]_T + K_D - \sqrt{([P]_T + [L]_T + K_D)^2 - 4[P]_T[L]\_T}}{2[L]_T}$$

Equation 7

K_I values were determined as previously described for Langerin and the DC-SIGN CRD.[6] Briefly, competitive binding experiments were conducted with **49** at a concentration of 0.1 mM and varying competitor concentrations $[I]_T$. K_I and $[P]_T$ values were derived from **Equation 8** in a two-parameter fit.

$$R_{2,obs} = R_{2,f} + \left(R_{2,b} - R_{2,f}\right)p_b$$

with

$$p_b = \frac{2cos\left(\frac{\theta}{3}\right)\sqrt{a^2 - 3b} - a}{3K_D + 2cos\left(\frac{\theta}{3}\right)\sqrt{a^2 - 3b} - a}$$

and

$$\theta = cos^{-1}\left(\frac{-2a^3 + 9ab - 27c}{2\sqrt{(a^2 - 3b)^3}}\right), a = K_D + K_I + [L]_T + [I]_T - [P]_T$$

$$b = ([I]_T - [P]_T)K_D + ([L]_T - [P]_T)K_D + K_I K_D, c = -K_I K_D [P]_T$$

Equation 8

K_D determination from ^{19}F NMR CSPs

For K_D determination from ^{19}F NMR CSPs, samples and measurements were set up as described for ^{19}F R_2-filtered NMR but at a single relaxation time T = 0 s. $CSP_{19F,\,T}$ were calculated from the chemical shift of the ^{19}F resonance of **48** in the presence of protein δ_T at increasing ligand concentrations and the chemical shift in absence of protein δ_f at a concentration of 1 mM via **Equation 4**.

$$CSP_{19F,T} = \delta_T - \delta_f$$

Equation 9

The K_D value was calculated from $CSP_{19F,\,T}$ values in a two-parameter fit using **Equation 10**, using a fixed protein concentration $[P]_T$ and a CSP_{19F} of 0 ppm as a lower bound. $CSP_{19F,b}$ corresponds to the top asymptote and was kept variable. As in **Equation 9**, p_b represents the fraction of bound protein.

$$CSP_{19F,T} = CSP_{19F,b}\, p_b$$

with

$$p_b = \frac{[P]_T + [L]_T + K_D - \sqrt{([P]_T + [L]_T + K_D)^2 - 4[P]_T[L]_T}}{2[L]_T}$$

Equation 10

STD NMR

Prior to STD NMR experiments, DC-SIGN ECD protein samples were dialyzed four times against 5 L ultrapure water overnight at 4°C. Water was removed by lyophilization and the protein was dissolved in 25 mM Tris-d_{11} (Eurisotope), 150 mM NaCl, 2 mM $CaCl_2$ in 100% D_2O at pH 7.8 and stored at 4°C. For measurements, samples contained 25 µM DC-SIGN ECD and 0.5 mM **48**. If applicable, 4 mM EDTA-d_{12} or 50 mM **Man-d_7** (Omicron) were added. 0.1 mM TSP-d_6 served as internal reference.

Selective saturation of protein resonances was achieved using a train of Gauss-shaped saturation pulses at a resonance irradiation frequency of 80.0 ppm for off-resonance spectra and 0.0 ppm for on-resonance spectra. Acquisition time was set to 2.0 s and solvent suppression was implemented *via* the 3-9-19 WATERGATE pulse sequence.[33] Direct saturation of the ligand was excluded by ^{1}H STD NMR measurements of **48** without protein. For epitope mapping, spectra were recorded at four different saturation times t_{sat} ranging from 0.5 to 6 s with varying number of scans (0.5 s: 2048 scans; 1 s: 1024 scans; 2 s: 512 scans; 6 s: 128 scans) at a relaxation delay d_1 of 6 s. In experiments involving EDTA or **Man** inhibition, 512 scans were recorded, t_{sat} was set to 2 s and d_1 was set to 2 s.

STD effects for each analyzed proton at respective t_{sat} were calculated from **Equation 1** as described above. The maximal STD effects STD_{max} and the observed saturation rates (k_{sat}) where determined from fitting **Equation 2** to obtained STD build-up curves in a two-parameter fit. Initial slopes of the build-up curves (STD'_0) were determined from **Equation 3** and normalized to the highest STD'_0.[34] Normalized STD'_0 values were mapped onto the structure of **48** based on a previous resonance assignment of **42**.[43]

^{15}N HSQC NMR

For ^{15}N HSQC NMR experiments, samples contained 0.2 mM ^{15}N-labeled DC-SIGN CRD WT or M270F in MES with 10 mM $CaCl_2$ in presence of 10% D_2O at pH 6.0 and varying concentrations of ligand. 0.1 mM DSS-d_6 served as internal reference. Prior to titrations probing the Ca^{2+}-independent binding of **48**, DC-SIGN CRD WT or M270F were dialyzed against 1 L of Chelex-filtered, Ca^{2+}-free MES with 1 mM EDTA at pH 6.0 twice.

All spectra were acquired with 128 increments and 12 scans per increment. The relaxation delay d1 was set to 1.4 s, saturation time t_{sat} to 100 ms and solvent suppression was implemented *via* the W5 WATERGATE pulse sequence.[39] A previously published resonance assignment of DC-SIGN CRD was transferred to the nearest neighbor in a reference spectrum recorded without ligand.[44] Peaks that were not assigned in the reference spectrum were numbered. Overlapping or disappearing peaks were not assigned.

CSPs for DC-SIGN CRD resonances showing fast and fast-to-intermediate exchange upon ligand titration were calculated using **Equation 4** as described above.[40] Binding-mode analysis was conducted by mapping CSPs corresponding to the highest ligand concentration on the X-ray structure of DC-SIGN CRD (PDB code: 1SL4) using the PyMOL script *data2bfactors* (available from: www.pldserver1.biochem.queensu.ca /~rlc/work/pymol/) by substituting the B-factor values. In case resonances vanished or showed reduced intensity upon titration, CSP values were set to 0.03 ppm for experiments with **48** and to 0.1 ppm for **Man**. To analyze the change of CSPs in ^{15}N HSQC NMR experiments under inhibitory conditions, ΔCSPs were determined by subtracting observed CSPs under noninhibitory conditions from those observed under CBS inhibitory conditions. Resonances in fast exchange showing CSPs > 0.015 ppm at the highest ligand concentration, were used to calculate K_D values in a global two-parameter fit via **Equation 5** as described above.[40]

Molecular docking

Langerin – carbohydrate binding site (CBS). Molecular modelling procedures were performed in MOE (Chemical Computing Group). Deviations from default options and parameters are noted. The AMBER10:EHT force field was selected for the refinement of docking poses and the hydrogen bond network while the MMFF94x force field was utilized for the generation conformers.[45–47] Receptor surfaces were visualized in Connolly representation.[41]

A structural alignment of the Langerin CBS in complex with different **Man**-type oligosaccharides was performed (PDB codes: 3P5D, 3P5E and 3P5F).[48] Based on this visualization, a pharmacophore model was defined with features for O3, O4 and O5 of the Man scaffold. The spatial constraint on the O3 and O4 was defined by a sphere with a radius r of 0.5 Å while the position of O5 was constrained by a sphere with a radius r of 1.0 Å. Chain B of the Langerin CRD in complex with a **Man**-type disaccharide served as the structural basis for the docking of **43** (PDB code: 3P5F). Of the two binding modes included in this model, the orientation for targeting the identified pockets in axial direction of C2 was selected. Additionally, an alternative conformation for K313 observed for the Langerin complex with Gal-6-OS was modeled and included into the analysis.[48] Overall model quality and protein geometry were evaluated in MolProbity and maintained utilized MOE's Structure Preparation.[49] Next, protonation states and

the hydrogen bond network of the complex were simulated with MOE's Protonate 3D followed by the removal of all solvent molecules.

Conformations for **43** were generated utilizing MOE's Conformation Import. A pharmacophore-based placement method was utilized to generate docking poses that we scored using the London ΔG function. Highly scored poses were refined utilizing molecular mechanics simulations, rescored via the GBIV/WSA ΔG function, filtered using the pharmacophore model and written into the output database.[50] Conformational flexibility of the CBS was accounted for by introducing B-factor-derived tethers to side chain atoms. Refined docking poses were ranked according to their the GBIV/WSA ΔG score and evaluated visually in the context of the conducted ^{15}N HSQC and STD NMR experiments.

DC-SIGN – secondary binding pocket. A model of ligand **48** was built and optimized using the VMD Molefacture plugin, and the X-ray structure of the DC-SIGN CRD (PDB code: 1SL4) was used as a receptor structure.[51,52] Only protein residues and Ca^{2+} ions were kept while structural waters were removed. The receptor and ligand structures were prepared following the standard AutoDock protocol.[53] All non-polar hydrogens were merged, and Gasteiger charges and atom types were added. The grid size and position were chosen to include all the amino acids belonging to the secondary binding pocket (Q306, M270, Y268, T261, F302, F269, and I124) and the spacing between grid points was set at 0.375 Å. AutoDock Bias protocol was applied to perform a biased docking experiment, considering the information from NMR experiments (ΔCSPs).[54] Briefly, based on ideal interaction estimated using ideal_interaction_sites.py, a hydrogen bond donor and acceptor restraint were added by modifying their respective energy grids (HD and OA map, respectively) using *prepare_bias.py* script.[55,56] For each system, 100 different docking runs were performed and the results were clustered according to the ligand heavy atom RMSD using a cut-off of 2 Å. The Lamarckian Genetic Algorithm parameters for each conformational search run were kept at their default values (150 for initial population size, $1{\cdot}10^7$ as the maximum number of energy evaluations, and $2.7{\cdot}10^4$ as the maximum number of generations). The docking results for **48** were further analyzed by visual inspection.

MD simulations

The complex between **48** and DC-SIGN was further analyzed using the protocol described by Blanco *et al.* with the modifications described below.[57] Briefly, the system was prepared with the leap module from the AMBER package using ff14SB and TIP3P force field for amino acid and water molecules.[58] The BMP parameter was obtained using the Antechamber module from the AMBER package using the GAFF force field. The system was first optimized using a conjugate gradient algorithm for 5000 steps, followed by 150 ps. Long constant volume MD equilibration, in which the first 100 ps were used to gradually raise the temperature of the system from 0 to 300 K (integration

step = 0.0005 ps per step). The heating was followed by a 300 ps long constant temperature and constant pressure MD simulation to equilibrate the system density (integration step = 0.001 ps per step). During these temperature and density equilibration processes, the protein α-carbon atoms were constrained by 5 $kcal \cdot mol^{-1} \cdot Å^{-1}$ force constant using a harmonic potential centered at each atom starting position. Next, a second equilibration MD of 5 ns was performed, in which the integration step was increased to 2 fs using the SHAKE algorithm, and the force constant for restrained α-carbons was decreased to 2 $kcal \cdot mol^{-1} \cdot Å^{-1}$ followed by 5 ns long MD simulation with no constraints. Finally, 20 ns long production MD simulations were carried out using the 'Hydrogen Mass Repartition' method, which allows an integration step of 4 fs.[59] The trajectory processing and RMSD analysis were performed with the CPPTRAJ module of the AMBER package.[60]

Webserver-based allosteric site prediction

The AllositePro server was used to predict potential allosteric binding sites in the DC-SIGN CRD X-ray structure (PDB code: 1SL4).[10,11] Binding sites were defined as allosteric based on an Allosite score of > 0.5 detected pockets, resulting from the weighted sum of a feature score, describing structural features physicochemical properties of the pocket, and a perturbation score derived from significant changes in normal mode analysis of apo and holo states of the protein.[10]

Supporting References

(1) Scharenberg, M.; Schwardt, O.; Rabbani, S.; Ernst, B. Target Selectivity of FimH Antagonists. *J. Med. Chem.* **2012**, *55* (22), 9810–9816.

(2) Klein, T.; Abgottspon, D.; Wittwer, M.; Rabbani, S.; Herold, J.; Jiang, X.; Kleeb, S.; Lüthi, C.; Scharenberg, M.; Bezençon, J.; Gubler, E.; Pang, L.; Smiesko, M.; Cutting, B.; Schwardt, O.; Ernst, B. FimH Antagonists for the Oral Treatment of Urinary Tract Infections: From Design and Synthesis to in Vitro and in Vivo Evaluation. *J. Med. Chem.* **2010**, *53* (24), 8627–8641.

(3) Navarra, G.; Zihlmann, P.; Jakob, R. P.; Stangier, K.; Preston, R. C.; Rabbani, S.; Smiesko, M.; Wagner, B.; Maier, T.; Ernst, B. Carbohydrate–Lectin Interactions: An Unexpected Contribution to Affinity. *ChemBioChem* **2017**, *18* (6), 539–544.

(4) Wamhoff, E. C.; Schulze, J.; Bellmann, L.; Rentzsch, M.; Bachem, G.; Fuchsberger, F. F.; Rademacher, J.; Hermann, M.; Del Frari, B.; Van Dalen, R.; Hartmann, D.; Van Sorge, N. M.; Seitz, O.; Stoitzner, P.; Rademacher, C. A Specific, Glycomimetic Langerin Ligand for Human Langerhans Cell Targeting. *ACS Cent. Sci.* **2019**, *5* (5), 808–820.

(5) Feinberg, H.; Taylor, M. E.; Razi, N.; McBride, R.; Knirel, Y. A.; Graham, S. A.; Drickamer, K.; Weis, W. I. Structural Basis for Langerin Recognition of Diverse Pathogen and Mammalian Glycans through a Single Binding Site. *J. Mol. Biol.* **2011**, *405* (4), 1027–1039.

(6) Wamhoff, E. C.; Hanske, J.; Schnirch, L.; Aretz, J.; Grube, M.; Varón Silva, D.; Rademacher, C. 19F NMR-Guided Design of Glycomimetic Langerin Ligands. *ACS Chem. Biol.* **2016**, *11* (9), 2407–2413.

(7) Hanske, J.; Aleksić, S.; Ballaschk, M.; Jurk, M.; Shanina, E.; Beerbaum, M.; Schmieder, P.; Keller, B. G.; Rademacher, C. Intradomain Allosteric Network Modulates Calcium Affinity of the C-Type Lectin Receptor Langerin. *J. Am. Chem. Soc.* **2016**, *138* (37), 12176–12186.

(8) Holla, A.; Skerra, A. Comparative Analysis Reveals Selective Recognition of Glycans by the Dendritic Cell Receptors DC-SIGN and Langerin. *Protein Eng. Des. Sel.* **2011**, *24* (9), 659–669.

(9) Feinberg, H.; Castelli, R.; Drickamer, K.; Seeberger, P. H.; Weis, W. I. Multiple Modes of Binding Enhance the Affinity of DC-SIGN for High Mannose N-Linked Glycans Found on Viral Glycoproteins. *J. Biol. Chem.* **2007**, *282* (6), 4202–4209.

(10) Song, K.; Liu, X.; Huang, W.; Lu, S.; Shen, Q.; Zhang, L.; Zhang, J. Improved Method for the Identification and Validation of Allosteric Sites. *J. Chem. Inf. Model.* **2017**, *57* (9), 2358–2363.

(11) Huang, W.; Lu, S.; Huang, Z.; Liu, X.; Mou, L.; Luo, Y.; Zhao, Y.; Liu, Y.; Chen, Z.; Hou, T.; Zhang, J. Allosite: A Method for Predicting Allosteric Sites. *Bioinformatics* **2013**, *29* (18), 2357–2359.

(12) Jiang, X.; Abgottspon, D.; Kleeb, S.; Rabbani, S.; Scharenberg, M.; Wittwer, M.; Haug, M.; Schwardt, O.; Ernst, B. Antiadhesion Therapy for Urinary Tract Infections-A Balanced PK/PD Profile Proved to Be Key for Success. *J. Med. Chem.* **2012**, *55* (10), 4700–4713.

(13) Ernst, B.; Kleeb, S.; Pang, L.; Mayer, K.; Eris, D.; Sigl, A.; Zihlmann, P.; Preston, R. C.; Sharpe, T.; Jakob, R.; Abgottspon, D.; Aline S. Hutter, Meike Scharenberg, Xiaohua Jiang, Giulio Navarra, S. R.; Martin Smiesko, Nathalie

Lüdin, Jacqueline Bezencon, Oliver Schwardt, and T. M. FimH Antagonists: Bioisosteres to Improve the in Vitro and in Vivo PK/PD Profile. *J. Med. Chem* **2015**, *58*, 1–32.
(14) Pang, L.; Kleeb, S.; Lemme, K.; Rabbani, S.; Scharenberg, M.; Zalewski, A.; Schädler, F.; Schwardt, O.; Ernst, B. FimH Antagonists: Structure-Activity and Structure-Property Relationships for Biphenyl α-D-Mannopyranosides. *ChemMedChem* **2012**, *7* (8), 1404–1422.
(15) Schwardt, O.; Rabbani, S.; Hartmann, M.; Abgottspon, D.; Wittwer, M.; Kleeb, S.; Zalewski, A.; Smieško, M.; Cutting, B.; Ernst, B. Design, Synthesis and Biological Evaluation of Mannosyl Triazoles as FimH Antagonists. *Bioorganic Med. Chem.* **2011**, *19* (21), 6454–6473.
(16) Sommer, R.; Wagner, S.; Rox, K.; Varrot, A.; Hauck, D.; Wamhoff, E. C.; Schreiber, J.; Ryckmans, T.; Brunner, T.; Rademacher, C.; Hartmann, R. W.; Brönstrup, M.; Imberty, A.; Titz, A. Glycomimetic, Orally Bioavailable LecB Inhibitors Block Biofilm Formation of Pseudomonas Aeruginosa. *J. Am. Chem. Soc.* **2018**, *140* (7), 2537–2545.
(17) Hauck, D.; Joachim, I.; Frommeyer, B.; Varrot, A.; Philipp, B.; Möller, H. M.; Imberty, A.; Exner, T. E.; Titz, A. Discovery of Two Classes of Potent Glycomimetic Inhibitors of Pseudomonas Aeruginosa LecB with Distinct Binding Modes. *ACS Chem. Biol.* **2013**, *8* (8), 1775–1784.
(18) Delgado, J.; Radusky, L. G.; Cianferoni, D.; Serrano, L.; Valencia, A. FoldX 5.0: Working with RNA, Small Molecules and a New Graphical Interface. *Bioinformatics* **2019**, *35* (20), 4168–4169.
(19) Scott, I. L.; Market, R. V.; DeOrazio, R. J.; Meckler, H.; Kogan, T. P. Stereospecific α-D-Mannosylation. *Carbohydr. Res.* **1999**, *317* (1–4), 210–216.
(20) Wohlschlager, T.; Butschi, A.; Grassi, P.; Sutov, G.; Gauss, R.; Hauck, D.; Schmieder, S. S.; Knobel, M.; Titz, A.; Dell, A.; Haslam, S. M.; Hengartner, M. O.; Aebi, M.; Kuñzler, M. Methylated Glycans as Conserved Targets of Animal and Fungal Innate Defense. *Proc. Natl. Acad. Sci. U. S. A.* **2014**, *111* (27).
(21) Hayes, W.; Osborn, H. M. I.; Osborne, S. D.; Rastall, R. A.; Romagnoli, B. One-Pot Synthesis of Multivalent Arrays of Mannose Mono- and Disaccharides. *Tetrahedron* **2003**, *59* (40), 7983–7996.
(22) Guberman, M.; Bräutigam, M.; Seeberger, P. H. Automated Glycan Assembly of Lewis Type i and II Oligosaccharide Antigens. *Chem. Sci.* **2019**, *10* (21), 5634–5640.
(23) Wang, W.; Jin, C.; Guo, L.; Liu, Y.; Wan, Y.; Wang, X.; Li, L.; Zhao, W.; Wang, P. G. Preparation of Oligosaccharides by Homogenous Enzymatic Synthesis and Solid Phase Extraction. *Chem. Commun.* **2011**, *47* (40), 11240–11242.
(24) Petersen, B. O.; Vinogradov, E.; Kay, W.; Würtz, P.; Nyberg, N. T.; Duus, J.; Sørensen, O. W. H2BC: A New Technique for NMR Analysis of Complex Carbohydrates. *Carbohydr. Res.* **2006**, *341* (4), 550–556.
(25) Sommer, R.; Makshakova, O. N.; Wohlschlager, T.; Hutin, S.; Marsh, M.; Titz, A.; Künzler, M.; Varrot, A. Crystal Structures of Fungal Tectonin in Complex with O-Methylated Glycans Suggest Key Role in Innate Immune Defense. *Structure* **2018**, *26* (3), 391-402.e4.
(26) Chen, W. C.; Completo, G. C.; Sigal, D. S.; Crocker, P. R.; Saven, A.; Paulson, J. C. In Vivo Targeting of B-Cell Lymphoma with Glycan Ligands of CD22. *Blood* **2010**, *115* (23), 4778–4786.

(27) Schulze, J.; Rentzsch, M.; Kim, D.; Bellmann, L.; Stoitzner, P.; Rademacher, C. A Liposomal Platform for Delivery of a Protein Antigen to Langerin-Expressing Cells. *Biochemistry* **2019**, *58* (21), 2576–2580.
(28) Aretz, J.; Wamhoff, E. C.; Hanske, J.; Heymann, D.; Rademacher, C. Computational and Experimental Prediction of Human C-Type Lectin Receptor Druggability. *Front. Immunol.* **2014**, *5*, 323.
(29) Stambach, N. S.; Taylor, M. E. Characterization of Carbohydrate Recognition by Langerin, a C-Type Lectin of Langerhans Cell. *Glycobiology* **2003**, *13* (5), 401–410.
(30) Meiboom, S.; Gill, D. Modified Spin-Echo Method for Measuring Nuclear Relaxation Times. *Rev. Sci. Instrum.* **1958**, *29* (8), 688–691.
(31) Deelchand, D. K.; Henry, P. G.; Marjańska, M. Effect of Carr-Purcell Refocusing Pulse Trains on Transverse Relaxation Times of Metabolites in Rat Brain at 9.4 Tesla. *Magn. Reson. Med.* **2015**, *73* (1), 13–20.
(32) Mayer, M.; Meyer, B. Characterization of Ligand Binding by Saturation Transfer Difference NMR Spectroscopy. *Angew. Chemie - Int. Ed.* **1999**, *38* (12), 1784–1788.
(33) Hwang, T. L.; Shaka, A. J. Water Suppression That Works. Excitation Sculpting Using Arbitrary Wave-Forms and Pulsed-Field Gradients. *J. Magn. Reson. - Ser. A* **1995**, *112* (2), 275–279.
(34) Mayer, M.; Meyer, B. Group Epitope Mapping by Saturation Transfer Difference NMR to Identify Segments of a Ligand in Direct Contact with a Protein Receptor. *J. Am. Chem. Soc.* **2001**, *123* (25), 6108–6117.
(35) Angulo, J.; Nieto, P. M. STD-NMR: Application to Transient Interactions between Biomolecules-a Quantitative Approach. *Eur. Biophys. J.* **2011**, *40* (12), 1357–1369.
(36) Bodenhausen, G.; Ruben, D. J. Natural Abundance Nitrogen-15 NMR by Enhanced Heteronuclear Spectroscopy. *Chem. Phys. Lett.* **1980**, *69* (1), 185–189.
(37) Delaglio, F.; Grzesiek, S.; Vuister, G. W.; Zhu, G.; Pfeifer, J.; Bax, A. NMRPipe: A Multidimensional Spectral Processing System Based on UNIX Pipes. *J. Biomol. NMR* **1995**, *6* (3), 277–293.
(38) Vranken, W. F.; Boucher, W.; Stevens, T. J.; Fogh, R. H.; Pajon, A.; Llinas, M.; Ulrich, E. L.; Markley, J. L.; Ionides, J.; Laue, E. D. The CCPN Data Model for NMR Spectroscopy: Development of a Software Pipeline. *Proteins Struct. Funct. Genet.* **2005**, *59* (4), 687–696.
(39) Liu, M.; Mao, X. A.; Ye, C.; Huang, H.; Nicholson, J. K.; Lindon, J. C. Improved Watergate Pulse Sequences for Solvent Suppression in NMR Spectroscopy. *J. Magn. Reson.* **1998**, *132* (1), 125–129.
(40) Williamson, M. P. Using Chemical Shift Perturbation to Characterise Ligand Binding. *Prog. Nucl. Magn. Reson. Spectrosc.* **2013**, *73*, 1–16.
(41) Connolly, M. L. The Molecular Surface Package. *J. Mol. Graph.* **1993**, *11* (2), 139–141.
(42) Carr, H. Y.; Purcell, E. M. Effects of Diffusion on Free Precession in Nuclear Magnetic Resonance Experiments. *Phys. Rev.* **1954**, *94* (3), 630–638.
(43) Wamhoff, E.-C. Glycomimetic Langerin Ligands for Langerhans Cell Targeting, Freie Universität Berlin, 2018.
(44) Pederson, K.; Mitchell, D. A.; Prestegard, J. H. Structural Characterization of

the DC-SIGN-LewisX Complex. *Biochemistry* **2014**, *53* (35), 5700–5709.
(45) Gerber, P. R.; Müller, K. MAB, a Generally Applicable Molecular Force Field for Structure Modelling in Medicinal Chemistry. *J. Comput. Aided. Mol. Des.* **1995**, *9* (3), 251–268.
(46) Case, D. A.; Darden Thomas Cheatham III Carlos Simmerling Junmei Wang, T. E.; Duke, R. E.; Crowley Ross Walker Wei Zhang Kenneth Merz Bing Wang Seth Hayik Adrian Roitberg Gustavo Seabra István Kolossváry Budapest, M. M.; Shaw Kim Wong, D. F.; Paesani, F.; Vanicek Xiongwu Wu Scott Brozell Thomas Steinbrecher Holger Gohlke Lijiang Yang Chunhu Tan John Mongan Viktor Hornak Guanglei Cui David H Mathews Matthew G Seetin Celeste Sagui Volodymyr Babin Peter A Kollman, J. R.; Pearlman Robert V Stanton Jed Pitera Irina Massova Ailan Cheng James J Vincent Paul Beroza Vickie Tsui Christian Schafmeister Wilson S Ross Randall Radmer George L Seibel James W Caldwell U Chandra Singh Paul Weiner, D. A.; Cieplak Yong Duan Rob Woods Karl Kirschner Sarah Tschampel Alexey Onufriev Christopher Bayly Wendy Cornell Scott Weiner Austin Yongye Matthew Tessier, P. M. *Amber 10 Users' Manual Principal Contributors to the Current Codes: Additional Key Contributors to Earlier Versions: Additional Key People Involved in Force Field Development*; 2008.
(47) Halgren, T. A. Merck Molecular Force Field. I. Basis, Form, Scope, Parameterization, and Performance of MMFF94. *J. Comput. Chem.* **1996**, *17* (5–6), 490–519.
(48) Feinberg, H.; Rowntree, T. J. W.; Tan, S. L. W.; Drickamer, K.; Weis, W. I.; Taylor, M. E. Common Polymorphisms in Human Langerin Change Specificity for Glycan Ligands. *J. Biol. Chem.* **2013**, *288* (52), 36762–36771.
(49) Chen, V. B.; Arendall, W. B.; Headd, J. J.; Keedy, D. A.; Immormino, R. M.; Kapral, G. J.; Murray, L. W.; Richardson, J. S.; Richardson, D. C. MolProbity: All-Atom Structure Validation for Macromolecular Crystallography. *Acta Crystallogr. Sect. D Biol. Crystallogr.* **2010**, *66* (1), 12–21.
(50) Corbeil, C. R.; Williams, C. I.; Labute, P. Variability in Docking Success Rates Due to Dataset Preparation. *Journal of Computer-Aided Molecular Design*. J Comput Aided Mol Des June 2012, pp 775–786.
(51) Guo, Y.; Feinberg, H.; Conroy, E.; Mitchell, D. A.; Alvarez, R.; Blixt, O.; Taylor, M. E.; Weis, W. I.; Drickamer, K. Structural Basis for Distinct Ligand-Binding and Targeting Properties of the Receptors DC-SIGN and DC-SIGNR. *Nat. Struct. Mol. Biol.* **2004**, *11* (7), 591–598.
(52) Humphrey, W.; Dalke, A.; Schulten, K. VMD: Visual Molecular Dynamics. *J. Mol. Graph.* **1996**, *14* (1), 33–38.
(53) Forli, S.; Huey, R.; Pique, M. E.; Sanner, M. F.; Goodsell, D. S.; Olson, A. J. Computational Protein-Ligand Docking and Virtual Drug Screening with the AutoDock Suite. *Nat. Protoc.* **2016**, *11* (5), 905–919.
(54) Arcon, J. P.; Modenutti, C. P.; Avendaño, D.; Lopez, E. D.; Defelipe, L. A.; Ambrosio, F. A.; Turjanski, A. G.; Forli, S.; Marti, M. A. AutoDock Bias: Improving Binding Mode Prediction and Virtual Screening Using Known Protein-Ligand Interactions. *Bioinformatics* **2019**, *35* (19), 3836–3838.
(55) Arcon, J. P.; Defelipe, L. A.; Modenutti, C. P.; López, E. D.; Alvarez-Garcia, D.; Barril, X.; Turjanski, A. G.; Martí, M. A. Molecular Dynamics in Mixed Solvents Reveals Protein-Ligand Interactions, Improves Docking, and Allows Accurate

Binding Free Energy Predictions. *J. Chem. Inf. Model.* **2017**, *57* (4), 846–863.
(56) Arcon, J. P.; Defelipe, L. A.; Lopez, E. D.; Burastero, O.; Modenutti, C. P.; Barril, X.; Marti, M. A.; Turjanski, A. G. Cosolvent-Based Protein Pharmacophore for Ligand Enrichment in Virtual Screening. *J. Chem. Inf. Model.* **2019**, *59* (8), 3572–3583.
(57) Blanco Capurro, J. I.; Di Paola, M.; Gamarra, M. D.; Martí, M. A.; Modenutti, C. P. An Efficient Use of X-Ray Information, Homology Modeling, Molecular Dynamics and Knowledge-Based Docking Techniques to Predict Protein-Monosaccharide Complexes. *Glycobiology* **2018**, *29* (2), 124–136.
(58) Maier, J. A.; Martinez, C.; Kasavajhala, K.; Wickstrom, L.; Hauser, K. E.; Simmerling, C. Ff14SB: Improving the Accuracy of Protein Side Chain and Backbone Parameters from Ff99SB. *J. Chem. Theory Comput.* **2015**, *11* (8), 3696–3713.
(59) Hopkins, C. W.; Le Grand, S.; Walker, R. C.; Roitberg, A. E. Long-Time-Step Molecular Dynamics through Hydrogen Mass Repartitioning. *J. Chem. Theory Comput.* **2015**, *11* (4), 1864–1874.
(60) Roe, D. R.; Cheatham, T. E. PTRAJ and CPPTRAJ: Software for Processing and Analysis of Molecular Dynamics Trajectory Data. *J. Chem. Theory Comput.* **2013**, *9* (7), 3084–3095.
